# Mixed-method tutoring support improves learning outcomes of veterinary students in basic subjects

**DOI:** 10.1186/s12917-018-1330-6

**Published:** 2018-02-01

**Authors:** María J. García-Iglesias, Claudia Pérez-Martínez, César B. Gutiérrez-Martín, Raquel Díez-Laiz, Ana M. Sahagún-Prieto

**Affiliations:** 1 Department of Animal Health, Faculty of Veterinary Science, Institute of Biomedicine (IBIOMED), University of León, Campus de Vegazana, s/n, 24071 León, Spain; 2 Department of Animal Health, Faculty of Veterinary Science, University of León, Campus de Vegazana, s/n, 24071 León, Spain; 3 Department of Biomedical Sciences, Faculty of Veterinary Science, Institute of Biomedicine (IBIOMED), University of León, Campus de Vegazana, s/n, 24071 León, Spain

**Keywords:** Online tutoring support, Large class, Cytology and Histology, Veterinary Pharmacology, Learning strategy, Veterinary Science Degree

## Abstract

**Background:**

Tutoring is a useful tool in the university teaching-learning binomial, although its development is impaired in large classes. Recent improvements in information and communication technologies have made tutoring possible via the Internet. The aim of this study was to evaluate the efficacy of mixed-method academic tutoring in two basic subjects in Veterinary Science studies at the University of León (Spain) to optimize the usefulness of tutoring support in the college environment. This quasi-experimental study was firstly carried out as a pilot study in a small group of tutored students of “Cytology and Histology” (CH) (47/186; 25.3%) and “Veterinary Pharmacology” (VP) (33/141; 23.4%) subjects, and was implemented in a large class of CH the next academic year (150 students) while comparing the results with those obtained in a previous tutorless course (162 students). Tutored students were given access to online questionnaires with electronic feedback on each subject. In addition to traditional tutoring carried out in both tutored and tutorless students, the pilot study included three sessions of face-to-face tutoring in order to monitor the progress of students. Its efficacy was assessed by monitoring students’ examination scores and attendance as well as a satisfaction survey.

**Results:**

Although the examination attendance rate in the pilot study was not significantly different between tutored and tutorless groups in both subjects, an increase for numerical scores in tutored groups was observed, with a significant higher final score in VP (*p* = 0.001) and in the CH practice exams (first term, *p* = 0.009; final, *p* = 0.023). Good and merit scores were also better in tutored students with significant differences in VP (*p* = 0.005). Students felt comfortable with the tutoring service (100% in CH; 91.7% in VP). Implementation of this additional support in CH also resulted in a significant increase of attendance at the final exam in tutored courses (87.3% versus 77.2%; *p* = 0.026), scaled (*p* = 0.001) and numerical scores (final score, *p* = 0.001).

**Conclusions:**

Online tutoring support, together with conventional teaching methods, may be a useful method to incorporate student-centered learning in basic subjects in Veterinary Science.

**Electronic supplementary material:**

The online version of this article (doi: 10.1186/s12917-018-1330-6) contains supplementary material, which is available to authorized users.

## Background

Implementing the policies of the European Higher Education Area (EHEA) has resulted in significant changes in the learning process. Under the EHEA, teachers are not mere suppliers of knowledge but facilitators who help students receive integral training combining knowledge, attitudes and skills. The target is to place students at the core of the learning process [1].

This new approach considers tutoring as a methodological tool with great potential within the EHEA framework. Research suggests that tutoring has a positive effect in different areas of knowledge including Health Science at High School [2]. One of the tutorial areas that must be particularly enhanced is the academic tutoring [3], which should be conceived as a support for academic, professional and personal development of all college students. Its tools should not only facilitate the monitoring of the learning process by teachers but also serve as a corrective feedback mechanism for the students themselves. At Spanish University, where every teacher is by law obliged to dedicate 6 h a week to tutoring activities, time allocated to this practice has long been used to solve students’ queries [4]. It is usually carried out in face-to-face settings in which tutor and students meet at an agreed time and location. However, tutoring should not be simply reduced to “six office hours a week” but rather be seen as a more complex and global process. It is a fact, at least in Spain, that most students do not benefit from tutoring hours as they do not find them helpful and they do not match their needs.

Recent improvements in information and communication technologies (ICTs) have enabled online tutoring [5–7]. Therefore, additional online learning support to students could be a useful tool independent of space and time. Furthermore, it could increase student participation since the use of electronic resources could represent a less pressurized environment than face-to-face communication and taking into account their popularity among students [8]. Moreover, students can identify their strengths and weaknesses, which leads to a better performance and understanding of the subject [7] and promotes life-long learning activities [9].

Published literature in this field relies on subjective interpretation of cases to demonstrate tutorial success; however, there is a lack of objective research demonstrating its usefulness after implementation [10, 11]. There should be more studies on how online tutoring support quantitatively enhances learning outcomes and student motivation to participate in the learning process, mainly in overcrowded classes of Health Science subjects. Monitoring a tutoring program is important for several reasons [12]: a) ongoing evaluation of progress can motivate both the students and the tutor; b) feedback on student performance can be used to highlight effective methods and improve on ineffective ones; and c) evaluation results can also be used as an example to demonstrate the value of the program to interested parties. To our knowledge, only one pilot study providing effective experience on how online tutoring influences the learning process has been reported in veterinary studies in Spain, namely in “Microbiology” [13].

Taking the above into account, the aim of this study was to evaluate the usefulness of mixed-method tutoring (face-to-face and online) support conducted in two basic subjects of Veterinary Science to optimize the efficacy of tutoring support in the college environment.

## Methods

### Context

In Spain, students may register at the Faculty of Veterinary Sciences after graduating from high school and passing a national selective test. Only 120 students are admitted to the faculty each year based on their scores. The veterinary education programme lasts 5 years.

This study was possible thanks to students enrolled in two basic subjects taught at the Faculty of Veterinary Sciences of the University of León (Spain): “Cytology and Histology” (CH) and “Veterinary Pharmacology” (VP). Students can follow CH during the second semester of the Degree, whereas VP is taught in the fourth semester. CH is worth a total of 6 European credits (ECTS) and consists of theory (12 h) and practical training (48 h). This subject focuses on the morphological study of healthy cells (Cytology) and the association among cells to form tissues (Histology), organs and systems (Microscopic Anatomy). Examination methods include the assessment of practical and theoretical knowledge in both first term and final exams. In the first one, Cytology and Histology contents are evaluated by a written theoretical exam with short answers and another written practical exam in which students must identify cells and tissues using overhead digital images. The final exam evaluates microscopic anatomy contents and follows the same method described above together with another practical examination in which the identification and description of organs under light microscope is assessed. This final exam includes also a second-chance exam for those students who failed the first term one.

VP is also worth 6 ECTS and consists of 32 theoretical hours, 10 h focused on working groups and 18 h of practical training. VP focuses on the safe, effective and rational use of veterinary drugs, including general concepts on Pharmacology (Pharmacokinetics and Pharmacodynamics), as well as on the main pharmacological groups used to treat and prevent animal diseases. Exams consist of a multiple choice test and several short questions. As far as practical knowledge is concerned, students are subjected to continuous assessment, and only need to sit an exam in case they have failed this assessment.

### Pilot study

#### Participants

The number of participants (tutored group) was 47 out of 186 (25.3%) students (female/male: 35/12) in CH and 33 out of 141 (23.4%) (female/male: 17/16) in VP. Their age ranged approximately from 18 to 20 years old.

#### Study design and procedures

**Fig. 1:**
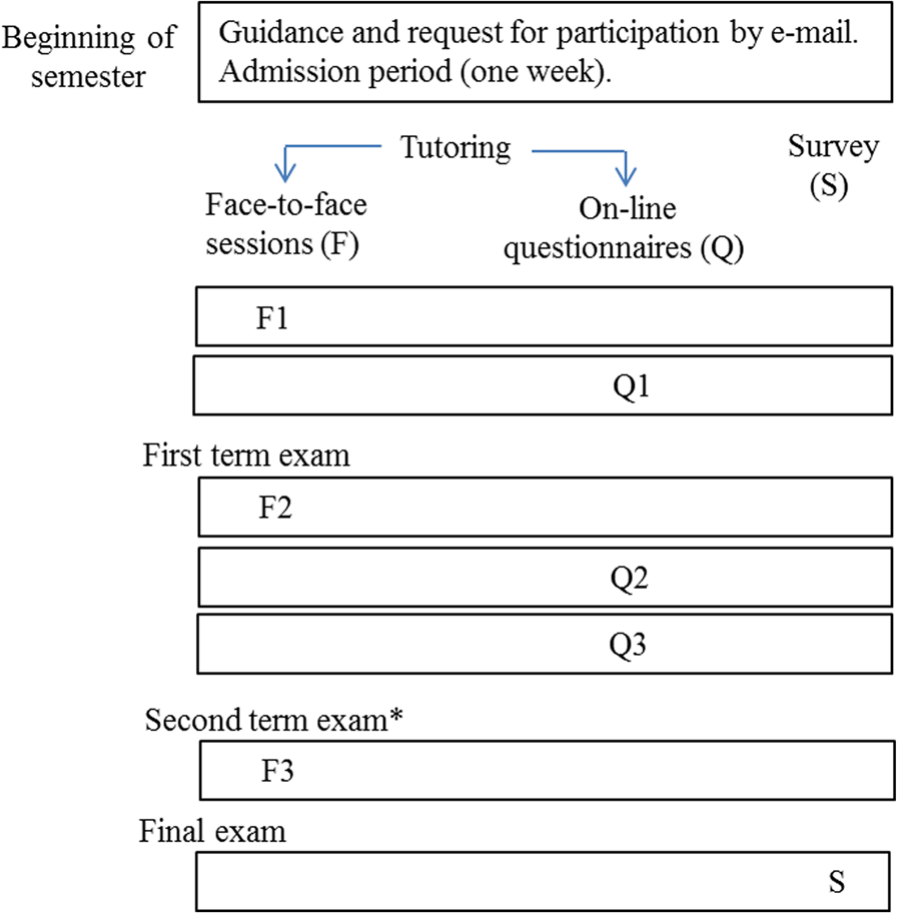
Schedule of the pilot study. CH, Cytology and Histology. VP, Veterinary Pharmacology. * only in Veterinary Pharmacology

A quasi-experimental design including online and face-to-face tutoring was assessed in both subjects during 2012–2013 academic year. All the students enrolled in CH and VP received an electronic mail inviting them to take part in the pilot study described below. Moreover, details about this pilot study were provided in the lecture-rooms at the beginning of the academic year. The students chose to participate on a voluntary basis after being explicitly informed in the information sessions. No incentives were offered for participation. The proposed protocol was first reviewed by peers within the Teaching Innovation Project Office, and its implementation was authorized as pilot study in both basic subjects. Besides, it was approved by each department (Animal Health and Biomedical Sciences) and the Dean of the Faculty of Veterinary Science The research design is shown in Fig. 1.

Both tutorless and tutored students received tutoring in which the tutor answered to spontaneous requests from students by electronic mail and individual face-to-face meetings. In addition, the tutored group received three one-hour sessions of face-to-face tutoring in a small group. The first one was scheduled after admission of students to inform them about the timetable and methodology before the pilot study began. The second face-to-face tutoring session was carried out after the first online questionnaire and the first term exam for progress monitoring purposes, and the third session was held at the end of the semester in order to solve doubts related to online questionnaires, the subject itself and the results of examinations.

**Fig. 2:**
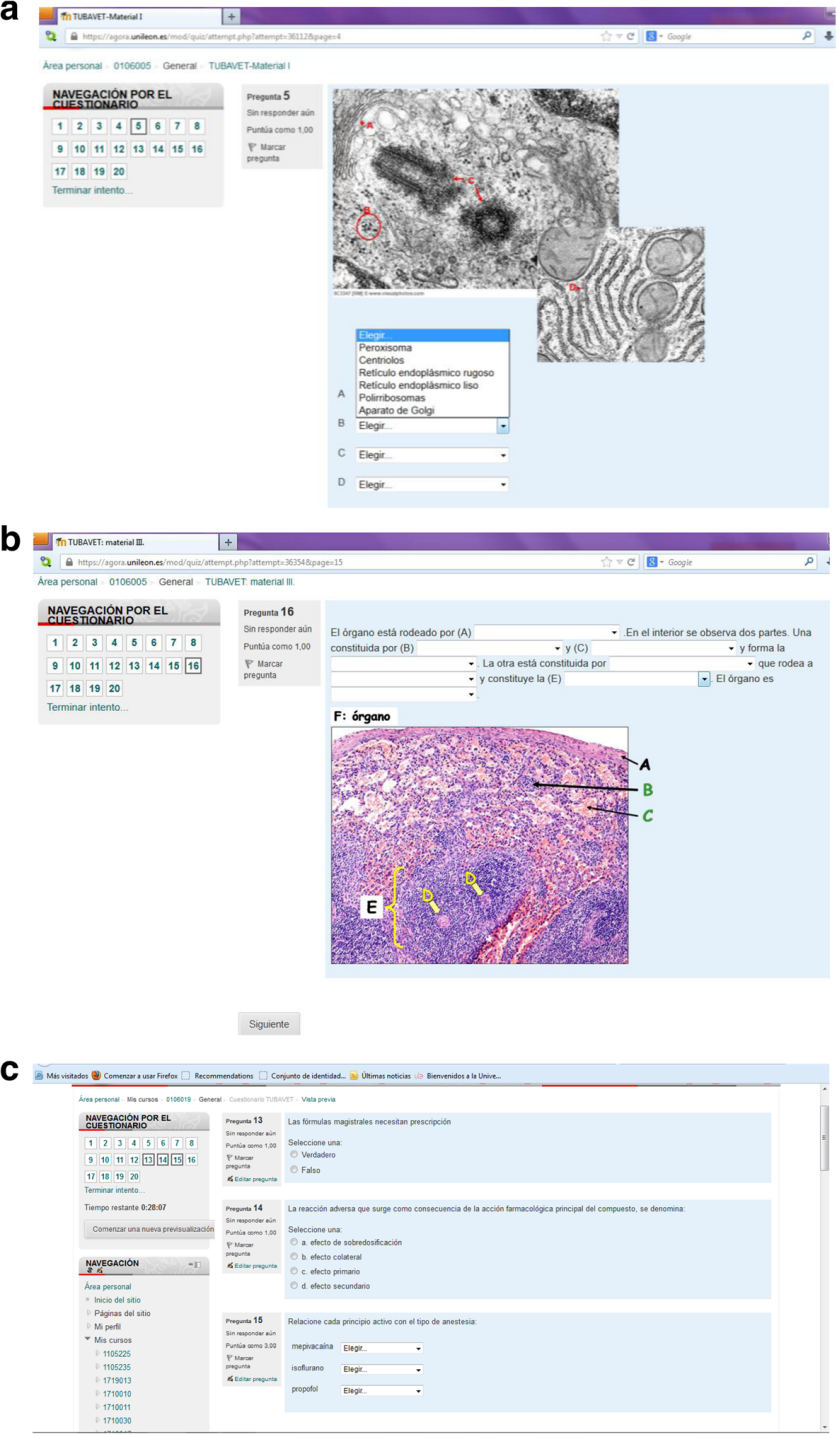
Different types of online questionnaires. **a** Embedded answers (gap fill). **b** matching. **c** true/false, multiple choice and matching tests

The questionnaires consisted of different types of exercises such as embedded (gap fill), true/false, multiple choice and matching answers (Fig. 2a–c and Additional file 1). Only the tutored group had access to the three questionnaires through the online course management system (Moodle). The availability of these questionnaires was limited to a certain period of time, and the students were only able to access the questionnaires once. Students received scores immediately after the first and third questionnaires had been fulfilled, and corrective feedback was provided once the tool was closed. As far as the second questionnaire is concerned, students took part in the correction of the test themselves by reviewing the results of one of their colleagues and giving the score. Further correction was also performed by the teachers at a later stage. After closing the questionnaires, a two-day period was established in order to answer and solve the doubts of the students via electronic mail.

#### Achievement criteria for study evaluation

The utility of the online tutoring support was assessed in three ways:

1. Student attendance to exams
2. Acquisition of content-specific knowledge evaluated through standard written exams in both tutored and unsupervised groups. The efficacy of this tutoring support on the learning process was assessed by monitoring exam scores based on a 10-point scale where 0 stands for the lowest score. Another parameter used for this matter was a scaled score obtained by grouping the 10-point scale into 4 categories as follows: <5 stands for “fail”, 5 to 6.9 stands for “pass”, 7 to 8.9 stands for “good” and ≥9 stands for “merit” [14].
3. An anonymous satisfaction survey including 7 items was designed to evaluate the student perception on the utility of combined tutoring and teacher skills. It was conducted via Moodle at the end of the learning innovation experience (Fig. 1). The items were valued on a four-level scale (“strongly agree”, “agree”, “neutral” and “disagree”). It also included a “free comment” section.

### Implementation study

The use of online tutoring was implemented in CH the next academic year (2013–2014). A similar design to the one described in the pilot study for online tutoring was followed. No face-to-face sessions in small groups were held due to the difficulty in scheduling this type of tutoring in overcrowded classes. Both tutored and tutorless courses had access to tutoring by electronic mail and individual face-to-face support, being online tutoring the main difference between both groups. This online activity was not mandatory in the tutored course, but was included in the evaluation criteria as continuous assessment and represented 4% of the overall score. The usefulness of this online service was assessed by comparing the learning outcomes between 150 (female/male: 102/48) tutored and 162 (female/male: 100/62) tutorless students enrolled in 2013–2014 and 2011–2012 academic courses respectively. Factors such as staff, specific content and timetable did not vary in both academic years.

### Statistical analysis

The qualitative variables (student attendance at exams, pass rates, and scale scores) were compared between tutored and tutorless groups through Chi-square test or Fisher’s exact test when appropriate. Numerical scores of written examinations, expressed as mean ± standard deviation (SD), were analysed through the Kolmogorov-Smirnov test for normal distribution. When data had normal distribution, the unpaired t-test was used. When data follow a non-normal distribution, the non-parametric Mann-Whitney U test was applied. The value of *p* ≤ 0.05 (two-tailed) was considered statistically significant. SPSS software version 21 (SPSS Inc., Chicago, IL, USA) for Windows was used to conduct the statistical analyses of the data. All data were used anonymously and treated confidentially according to the EU Data Protection Directive 95/46/EC.

## Results

### Pilot study

#### Answer to online questionnaires and results

The highest response rates of tutored students were reached in CH for the first and third online questionnaires, reaching 87.2 and 83.0% respectively as opposed to the second one, which was only answered by 53.2% of undergraduates. In VP, all students answered the two first online questionnaires, whereas the response rate for the third one dropped to 81.8% of undergraduates. Table 1 shows the numerical scores achieved for the three questionnaires in both subjects.

**Table 1:** Pilot study. Online questionnaires: percentage of tutored students that completed them and their numerical scores achieved

Online questionnaires	Cytology and Histology		Veterinary Pharmacology	
	Percentage of students	Scores (mean ± SD)	Percentage of students	Scores (mean ± SD)
First	87.2	8.87 ± 1.06	100	9.39 ± 0.74
Second	53.2	8.99 ± 0.96	100	7.36 ± 0.22
Third	83.0	8.72 ± 1.20	81.8	8.99 ± 1.16

#### Attendance at exams

A total of 117 out of 139 tutorless (84.2%) and 43 out of 47 tutored (91.5%) students took the first term exam in CH. The final exam was taken by 118 out of 137 (86.1%) unsupervised students and by 44 out of 46 (95.7%) tutored undergraduates. As a result, a rising trend attendance at exams was observed in the tutored groups in CH (Table 2). Nevertheless, a similar trend was not seen in the attendance at exams in VP in both first (32 out of 33, 97.0% in tutored students versus 107 out of 108, 99.1% in unsupervised group) and second (30 out of 33, 91.0% versus 104 out of 108, 96.3%, respectively) term exams (Table 2).

**Table 2:** Pilot study. Attendance, pass rates and scaled scores of tutored and tutorless students in exams

	Percentage of students		*p* value
	Tutorless group	Tutored group	
Cytology and Histology			
First term exam			
Attendance	84.2	91.5	0.157
Pass rate			
Practice	75.2	90.7	0.044
Theory	56.0	76.7	0.018
Total	50.4	74.4	0.007
Final exam			
Attendance	86.1	95.7	0.061
Pass rate			
Practice	88.0	93.2	0.406
Theory	76.3	77.3	1.000
Total	75.4	75.0	1.000
Scaled score			0.361
Fail	24.6	25.0	
Pass	39.8	27.3	
Good	33.9	43.2	
Merit	1.7	4.5	
Veterinary Pharmacology			
First term exam			
Attendance	99.1	97.0	0.074
Pass rate	78.7	84.8	0.619
Second term exam			
Attendance	96.3	91.0	0.212
Pass rate	45.4	60.6	0.164
Final exam^a^			
Pass rate	81.5	90.1	0.284
Scaled score			0.005
Fail	18.5	9.1	
Pass	54.6	30.3	
Good	22.2	48.5	
Merit	4.6	12.1	

^a^ Final examination only for students who failed previous exams

#### Influence of experimental design tutoring on learning outcomes

Evaluation of the exam pass rates (Table 2) revealed that tutoring tools significantly improved the outcomes in tutored students compared with those who followed a conventional instruction in all examinations conducted in CH for the first term exam. Regarding the exam pass rate in VP, a rising trend has been observed in all the examinations done (Table 2).

In VP, an important and significant improvement associated with the use of the experimental design tutoring system was also observed for scaled scores which, as expected, were better in tutored students than in the unsupervised group for “good” and “merit” scores. A similar trend was observed in CH subject although significant differences were not found.

The comparison of the numerical scores in both subjects for the tutored and tutorless groups is shown in Table 3. The tutored group in CH reached significantly higher scores than the unsupervised one in practical exams. Regarding the first term exam and overall evaluation in VP, scores were significantly higher in the tutored than in the unsupervised groups.

**Table 3:** Pilot study. Numerical scores achieved by tutored and tutorless students in exams

	Scores (Mean ± SD)		*p* value
	Tutorless group	Tutored group	
Cytology and Histology			
First term exam			
Practice	6.50 ± 1.91	7.37 ± 1.62	0.009
Theory	5.38 ± 1.73	5.95 ± 1.67	0.067
Final exam			
Practice	6.76 ± 1.67	7.42 ± 1.48	0.023
Theory	5.72 ± 1.46	6.03 ± 1.49	0.244
Final score	6.32 ± 1.45	6.76 ± 1.43	0.084
Veterinary Pharmacology^a^			
First term exam	5.73 ± 1.88	6.74 ± 1.66	0.038
Second term exam	4.58 ± 1.94	5.63 ± 1.80	0.100
Final exam	6.56 ± 1.14	7.38 ± 1.08	0.001

^a^ Theory exam

#### Student satisfaction survey

The satisfaction survey conducted after the final exam was completed by 25 out of the 47 tutored students (53.2%) in CH and by 12 out of 33 students (36.4%) in VP. Most of them agreed or strongly agreed with the utility of these innovative methodologies to improve the learning of both basic subjects (Table 4). Amongst students’ opinions, special attention should be drawn to the fact that tutors had been heavily engaged in the development of the pilot study and with the use of this tutoring tool as teaching methodology (Table 4). Students also agreed that tutoring sessions proved to be very advantageous to solve doubts about the subjects, prepare the exams and enhance the students’ motivation. In all, it is also noteworthy that almost all participants were satisfied with the use of this tutoring as learning methodology. Amongst the comments received, the greatest utility of online tests to improve the practice training in CH, the positive attitude of tutors and the satisfaction with the high level and quality of the online tutoring tool may be highlighted.

**Table 4:** Pilot study. Online satisfaction survey on the experimental design tutoring support

Questions and subjets	Four-level scale Percentage of students			
	Disagree	Neutral	Agree	Strongly agree
1. The number of face-to-face tutoring sessions has been appropriate				
Cytology and Histology	4.0	16.0	40.0	40.0
Veterinary Pharmacology	0.0	16.7	8.3	75.0
2. The number of online tutoring sessions has been appropriate				
Cytology and Histology	4.0	8.0	36.0	52.0
Veterinary Pharmacology	0.0	0.0	33.3	66.7
3. The degree of involvement of tutors has been high				
Cytology and Histology	0.0	0.0	12.0	88.0
Veterinary Pharmacology	0.0	0.0	33.3	66.7
4. The tutoring sessions help to solve doubts about the matter				
Cytology and Histology	0.0	12.0	48.0	40.0
Veterinary Pharmacology	8.3	8.3	33.3	50.0
5. The tutoring sessions help to prepare for exams				
Cytology and Histology	0.0	12.0	40.0	48.0
Veterinary Pharmacology	0.0	33.3	25.0	41.7
6. The tutoring sessions have improved the motivation-related factors				
Cytology and Histology	0.0	16.0	44.0	40.0
Veterinary Pharmacology	0.0	33.3	58.3	8.3
7. Overall, I am glad with the use of tutoring as a teaching methodology				
Cytology and Histology	0.0	0.0	32.0	68.0
Veterinary Pharmacology	0.0	8.3	41.7	50.0
8. Free comments:				
Cytology and Histology	- Online questionnaires are more useful to improve the practical training than the theory knowledge. - A positive attitude of tutors during online service increases the motivation of students. - Satisfaction with the level and quality of online tutoring.			
Veterinary Pharmacology	- I am really satisfied with the pilot study. It is useful to prepare the exams. - I think it has been an interesting activity.			

### Implementation study

The pilot study revealed positive outcomes after using this learning methodology, justifying thus its implementation in teaching large classes of undergraduates. For this reason, an online tutoring system was developed in CH the following academic year (2013–2014). When comparing the attendance of undergraduates at the first term exam, no significant differences were found between the tutorless (2011–2012) and tutored (2013–2014) courses, while a rising trend was observed in the pass rates (including theoretical exam and overall evaluation) (Table 5). With regard to the final examination, attendance was significantly higher in the tutored than in the tutorless courses and the percentage of students passing both practical and theoretical exams reflected a rising trend when online tutoring was carried out.

**Table 5:** Implementation study in Cytology and Histology. Attendance, pass rates and scaled scores of veterinary students from tutorless (2011-2012) and tutored (2013-14) courses

	Percentage of students		*p* value
	Tutorless group	Tutored group	
First term exam			
Attendance	95.1	93.3	0.629
Pass rate			
Practice	91.5	90.0	0.690
Theory	81.5	85.6	0.429
Total	79.7	85.6	0.218
Final exam			
Attendance	77.2	87.3	0.026
Pass rate			
Practice	87.9	91.6	0.409
Theory	86.4	89.3	0.566
Total	86.2	87.8	0.713
Scaled score			0.001
Fail	15.3	12.2	
Pass	56.5	26.0	
Good	25.8	57.3	
Merit	2.4	4.6	

Tables 5 and 6 show the learning results achieved by the undergraduates enrolled in tutorless (2011–2012) and tutored (2013–2014) courses. Percentage scaled scores significantly differed between both courses, showing higher scores for “good” and “merit” marks in the tutored course. When numerical scores were compared, students of the course in which the tutoring tool was implemented got significantly higher scores than those tutorless enrolled 2 years before.

**Table 6:** Implementation study in Cytology and Histology. Numerical scores achieved by veterinary students from tutorless (2011-2012) and tutored (2013-2014) courses

	Scores (Mean ± SD)		*p* value
	Tutorless group	Tutored group	
First term exam			
Practice	6.69 ± 1.60	7.08 ± 1.75	0.044
Theory	5.59 ± 1.55	6.21 ± 1.67	0.001
Final exam			
Practice	6.47 ± 1.80	7.14 ± 1.61	0.002
Theory	5.82 ± 1.69	6.57 ± 1.57	0.001
Final score	6.33 ± 1.53	7.08 ± 1.42	0.001

## Discussion

Bologna Process has had an impact on methodological tools used in higher education in the last decades. It has changed the teaching scene from a system mainly based on passive teacher’s lectures to one based on self-student learning. In this new framework, students have to assume new responsibilities to become autonomous and self-sufficient [15]. On the other hand, educators, more than being perceived as a source of knowledge, are expected to teach students how to study as well as to be initiative-oriented behavior and take their own decisions [16]. Undergraduates perceive as good teachers those that are close to students and active as motivators of the learning process. Tutoring is a methodological teaching tool with an enormous potential in the EHEA context. However, it is not easy to design new stimulating strategies that can be implemented in large classes of undergraduates. The use of Internet seems a way to solve this problem through online tutoring [17, 18], and Moodle is considered a powerful and flexible tool employed for monitoring and diagnosing the student work [19]. This method may be designed in different ways depending on the type of university degree and subject to be implemented such as: access to an online resource by addressing student questions [18], online problem-based learning [20] and online questionnaires for assessing the skills acquired by veterinary students [21].

The tutoring model presented in this study comprised two environments. One of them was represented by three online content-specific questionnaires proposed at different times during the development of both basic subjects and their feedback, which can be considered as instructional tutoring in which the tutor initiates or intervenes in the students’ learning process [22]. The second component of this study, also known as *reactive tutoring* [23], was represented by face-to-face settings in which the tutor answers to spontaneous requests for help from tutored students. The use of periodical online questionnaires to evaluate the acquisition of new knowledge by undergraduates is currently being carried out in subjects of Health Sciences. However, few research studies on its qualitative and quantitative usefulness have been undertaken to determine its real value at university level. For this reason, the novelty of our research is to have shown the quantitative and qualitative efficacy of a tool that is far from conventional tutoring and applicable to large classes of undergraduates. This has been first demonstrated in a small group pilot study and then confirmed after its implementation in an overcrowded class. To our knowledge, only one systematic investigation had been previously reported in a basic subject of Veterinary Science providing information on the influence of this type of online tutoring on the learning process, although it is to be noted that the study was only developed within a small group of students [16].

The poor opinion that undergraduates have on tutorial support was evidenced by the relatively low engagement to take part in the pilot study. However, those students who participated considered this experimental design tutoring as a very useful methodological tool. The high participation maintained over time is a good success indicator of this learning strategy. Slight decreases in response rates to complete the online content-specific questionnaires could be explained because of the long time passed between the activity itself and the examination of the subjects and/or the heavy workload at the end of the semester.

As mentioned above, several face-to-face tutoring sessions were also developed as a way to guide and help students to overcome any difficulties they could find with the methodological tool or the subject taught. Students considered these extra tutoring sessions as a positive and rewarding experience, in which teachers became learning guiders and motivators, and they thanked and greatly appreciated their implication. The information obtained in these sessions and in the satisfaction surveys was very useful for the implementation of this learning strategy in large classes. In this respect and taking into account the students’ comments in CH subject, the design of online questionnaires was modified to increase the motivation in theoretical training, compared with the pilot study by including more questions on theoretical knowledge. This modification might have contributed to the significant improvement in scaled and numerical scores achieved by the veterinary students during the implementation phase. Our experience supports the importance of students’ surveys for teaching enhancing, as previously cited [24]. However, the scarce participation of students in this sort of surveys suggests an absence of motivation since they consider that their opinions will not be taken into account. In addition, these surveys are usually tedious. For these reasons, higher education needs to make significant progress for undergraduates to perceive that their voices are heard as part of quality assurance [25].

The assessment of several parameters related to practical and theoretical training suggests that this mixed-method tutoring support was beneficial for the learning outcomes of veterinary students in the pilot study. However, it is to be noted that the results were not significant, probably due to the small size of the samples. These positive findings were supported by higher attendance at final exam and better numerical and scaled scores achieved after implementation in CH, even though overall pass rates did not raise. This improvement could be attributed to online questionnaires whose feedback may enhance students’ motivation and confidence to succeed in their examinations. Besides, this online support helped to maintain a daily work done by students on specific contents allowing them to identify shortcomings that could be solved at an early stage. Thus, online quizzes may indirectly induce the use of reactive tutoring in which the tutor answers to spontaneous requests by electronic mail or in individual face-to-face sessions. Hence, the effect of both tutoring systems (online and traditional) could be associated with an improvement in student outcomes and the poor opinion undergraduates have on tutoring support.

## Limitations of the study

Results reported above must be interpreted within the limits of the pilot and implementation studies. First of all, success may be partially attributed to the motivated students who voluntarily participated in this innovative strategy, which may also be related to the positive effects found in the pilot study, as other authors have mentioned [26]. However, statistically significant higher participation in final exam and better scores achieved by veterinary students when this learning tool was implemented in CH supports its positive impact. Secondly, the use of year-to-year comparisons in the implementation study may differ mainly as far as students are concerned, since other factors such as staff, contents and timetable did not vary from one academic year to the other. Finally, another potential influencing factor could be related to prior knowledge of questions and examination formats in tutored groups as opposed to tutorless ones.

## Conclusions

The results of this study reveal that an online tutoring tool in overcrowded classes with specific-content questionnaires, together with conventional teaching methods, results in (a) improved outcomes in terms of numerical and scaled scores, (b) a high satisfaction of undergraduates with the learning process and an increase in their motivation, and (c) a better theoretical and practical acquisition of knowledge. For all these reasons, the future implementation of this methodology in basic subjects in higher education with large numbers of students is strongly advised. It should logically be adapted to the particular idiosyncrasy of each subject.

## Additional file

Additional file 1: Online questionnaires on specific content of Cytology and Histology and Veterinary Pharmacology included in the experimental design tutoring for veterinary Editors-in-Chief. (PDF 10030 kb)

## Abbreviations

CH: Cytology and Histology
ECTS: European credit transfer and accumulation system
EHEA: European Higher Education Area
ICTs: Information and communication technologies
SD: Standard deviation
VP: Veterinary Pharmacology
