# Correction to: Mixed-method tutoring support improves learning outcomes of veterinary students in basic subjects

**DOI:** 10.1186/s12917-018-1380-9

**Published:** 2018-03-07

**Authors:** M. J. García-Iglesias, C. Pérez-Martínez, C. B. Gutiérrez-Martín, R. Díez-Laiz, A. M. Sahagún-Prieto

**Affiliations:** 1 Department of Animal Health, Faculty of Veterinary Science, Institute of Biomedicine (IBIOMED), University of León, Campus de Vegazana, s/n, 24071 León, Spain; 2 Department of Animal Health, Faculty of Veterinary Science, Institute of Biomedicine (IBIOMED), University of León, Campus de Vegazana, s/n, 24071 León, Spain; 3 Department of Animal Health, Faculty of Veterinary Science, University of León, Campus de Vegazana, s/n, 24071 León, Spain; 4 Department of Biomedical Sciences, Faculty of Veterinary Science, Institute of Biomedicine (IBIOMED), University of León, Campus de Vegazana, s/n, 24071 León, Spain

## Erratum

The original article [1] contains an error whereby Fig. 2a and b are mistakenly swapped with each other, and thus do not correspond to their correct respective sub-headings in the caption.

The correct version of Fig. 2a and b with their correct respective captions can be found below.

**Fig. 2:**
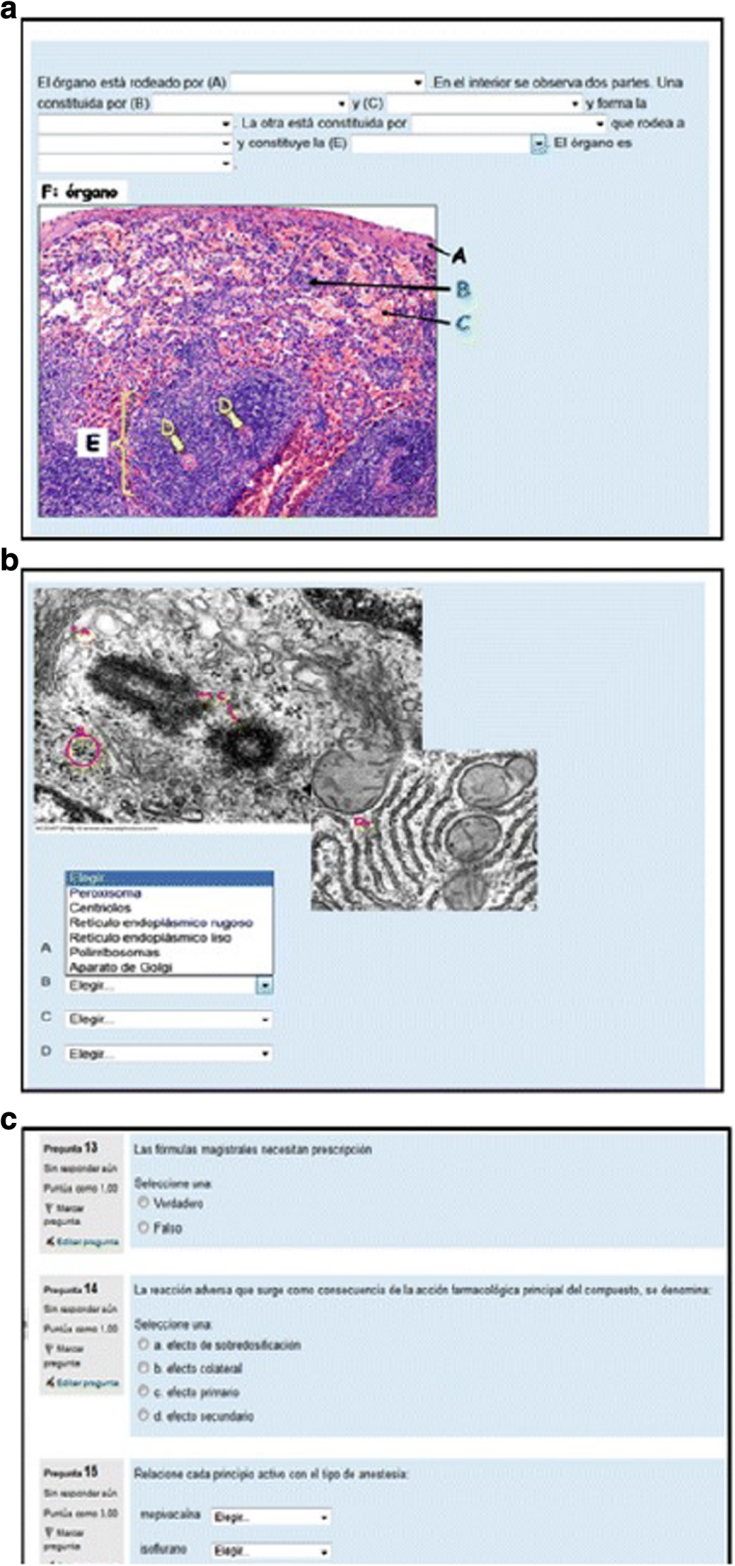
Different types of online questionnaires. **a** Embedded answers (gap fill). **b** matching. **c** true/false, multiple choice and matching tests
