# Supplementary material for: Mixed-method tutoring support improves learning outcomes of veterinary students in basic subjects
Source: BMC Vet Res. 2018 Feb 1;14:35. doi: 10.1186/s12917-018-1330-6 (PMC5796493; doi:10.1186/s12917-018-1330-6)
Supplement: Additional file 1: — Online questionnaires on specific content of Cytology and Histology and Veterinary Pharmacology included in the experimental design tutoring for veterinary Editors-in-Chief. (PDF 10030 kb) [file 12917_2018_1330_MOESM1_ESM.pdf]

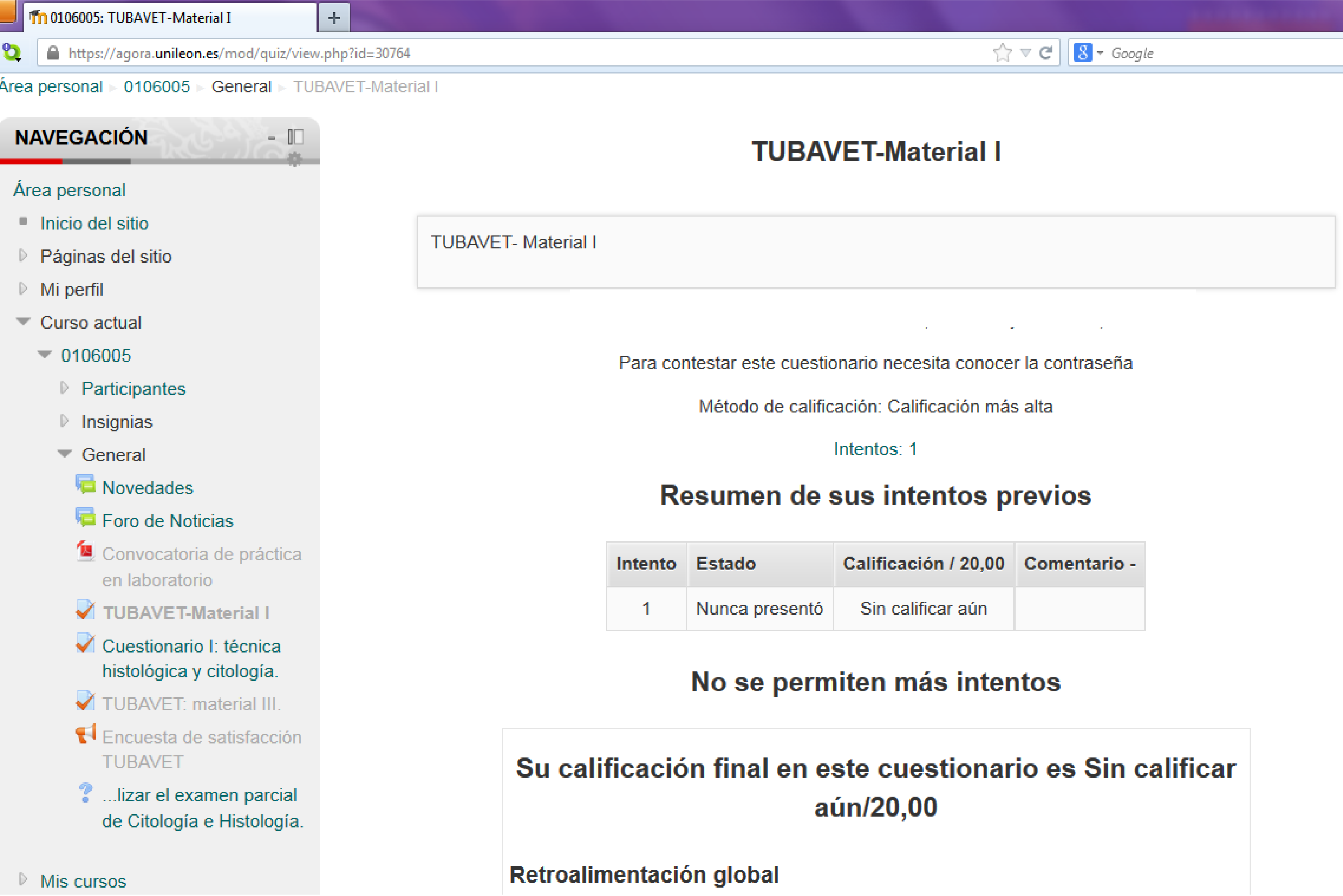

QUESTIONNAIRE I

Citology & Histology

TUBAVET-Material I

https://agora.unileon.es/mod/quiz/attempt.php?attempt=36112

Google

NAVEGACIÓN POR EL CUESTIONARIO

1	2	3	4	5	6	7	8
9	10	11	12	13	14	15	16
17	18	19	20				

Terminar intento...

Pregunta **1**

Sin responder aún

Puntúa como 1,00

Marcar pregunta

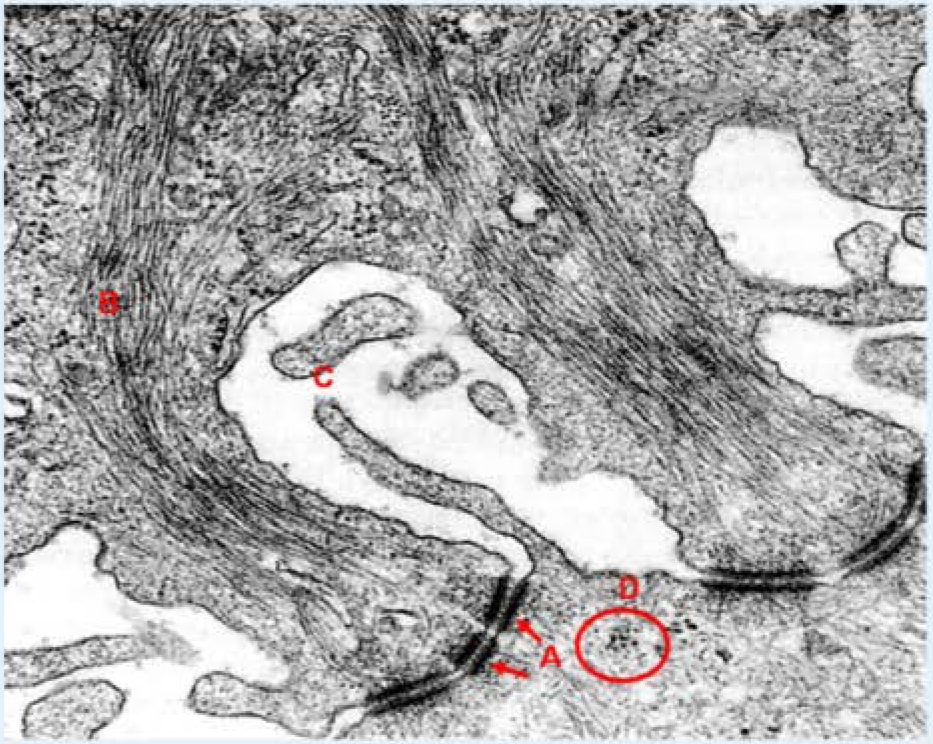

A Elegir...

B Elegir...

C Elegir...

D Elegir...

QUESTIONNAIRE I

Citology & Histology

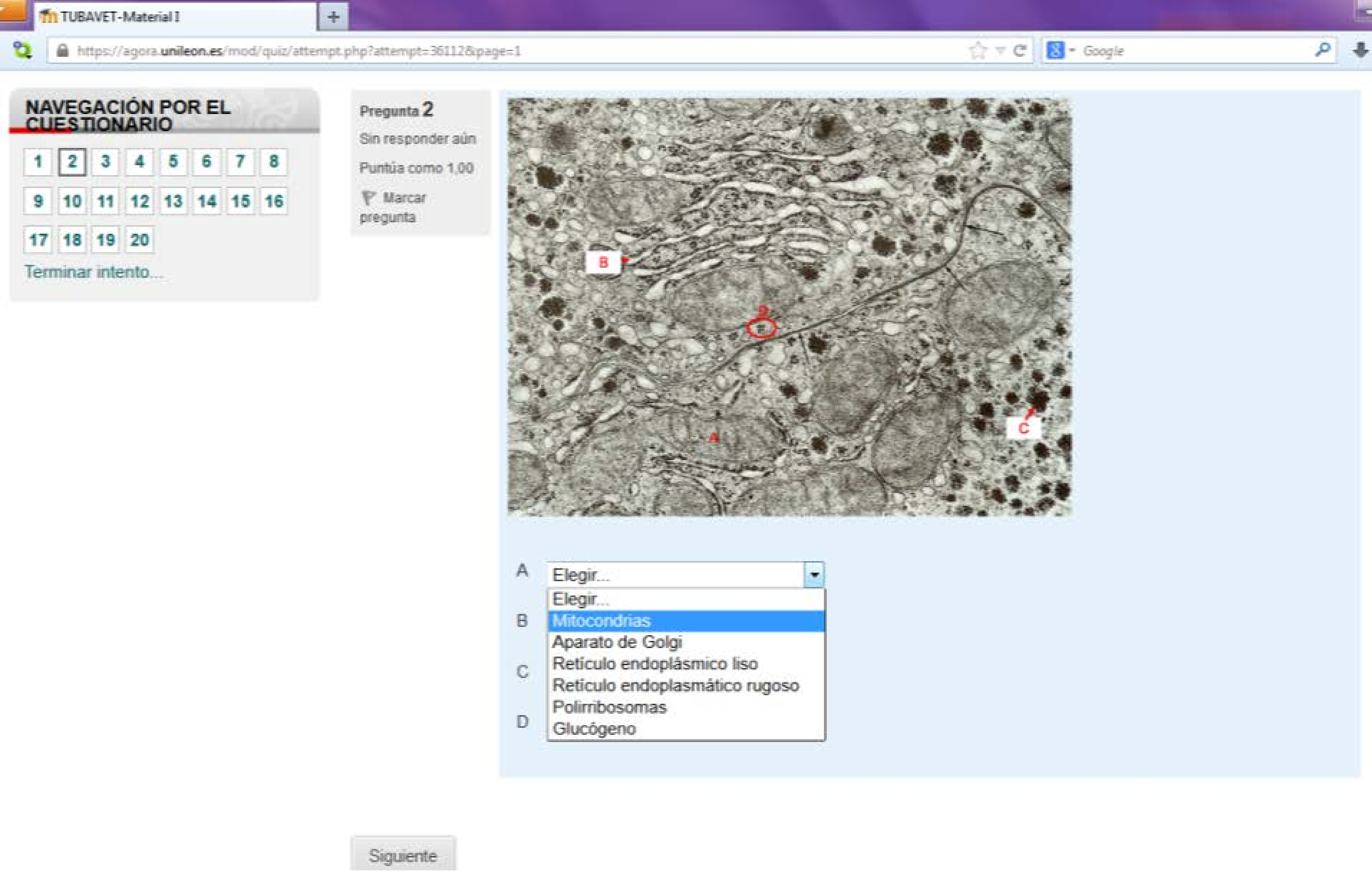

QUESTIONNAIRE I

Citology & Histology

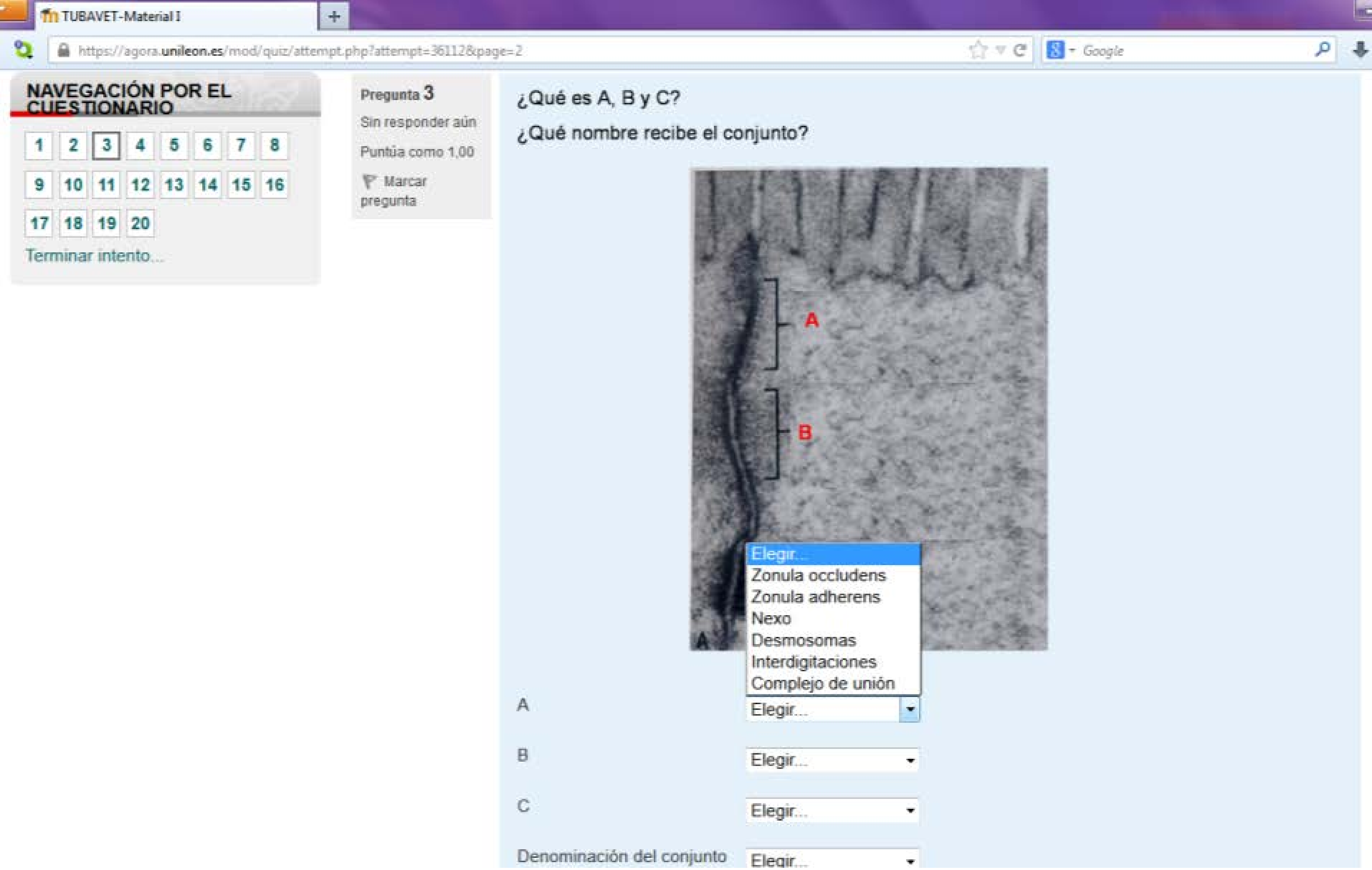

QUESTIONNAIRE I

Citology & Histology

TUBAVET-Material I

https://agora.unileon.es/mod/quiz/attempt.php?attempt=36112&page=3

Área personal ▸ 0106005 ▸ General ▸ TUBAVET-Material I

NAVEGACIÓN POR EL CUESTIONARIO

1 2 3 4 5 6 7 8
9 10 11 12 13 14 15 16
17 18 19 20

Terminar intento...

Pregunta 4

Sin responder aún

Puntúa como 1,00

Marcar pregunta

¿Qué estructura aparece en la imagen?

¿Qué es A, B y C?

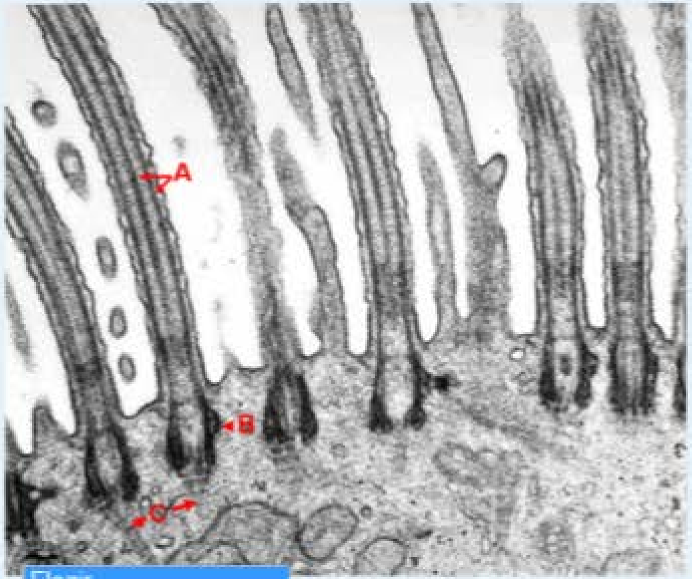

Estructura

- Elegir...
- Filamentos de actina
- Microtúbulos
- Raices ciliares
- Microvellosidades
- Cilios
- Cuerpo basal

A

B Elegir...

C Elegir...

QUESTIONNAIRE I

Citology & Histology

TUBAVET-Material I

https://agora.unileon.es/mod/quiz/attempt.php?attempt=36112&page=4

Área personal · 0106005 · General · TUBAVET-Material I

NAVEGACIÓN POR EL CUESTIONARIO

1 2 3 4 5 6 7 8
9 10 11 12 13 14 15 16
17 18 19 20

Terminar intento...

Pregunta 5

Sin responder aún

Puntúa como 1,00

Marcar pregunta

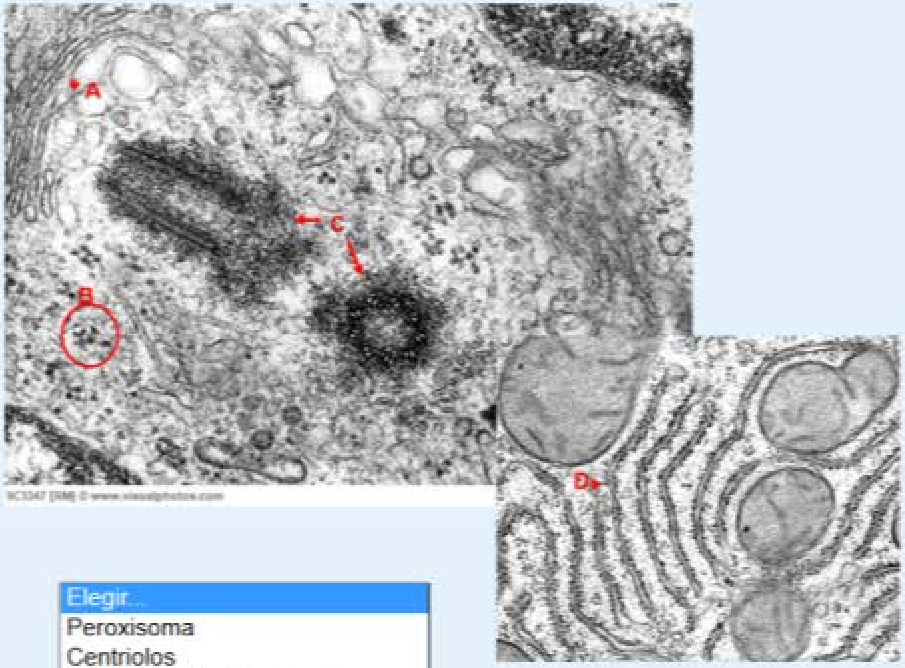

A
- Elegir...
- Peroxisoma
- Centriolos
- Retículo endoplásmico rugoso
- Retículo endoplásmico liso
- Polirribosomas
- Aparato de Golgi

B Elegir...

C Elegir...

D Elegir...

QUESTIONNAIRE I

Citology & Histology

TUBAVET-Material I

https://agora.unileon.es/mod/quiz/attempt.php?attempt=36112&page=5

Área personal ▸ 0106005 ▸ General ▸ TUBAVET-Material I

NAVEGACIÓN POR EL CUESTIONARIO

1 2 3 4 5 6 7 8
9 10 11 12 13 14 15 16
17 18 19 20

Terminar intento...

Pregunta 6

Sin responder aún

Puntúa como 1,00

Marcar pregunta

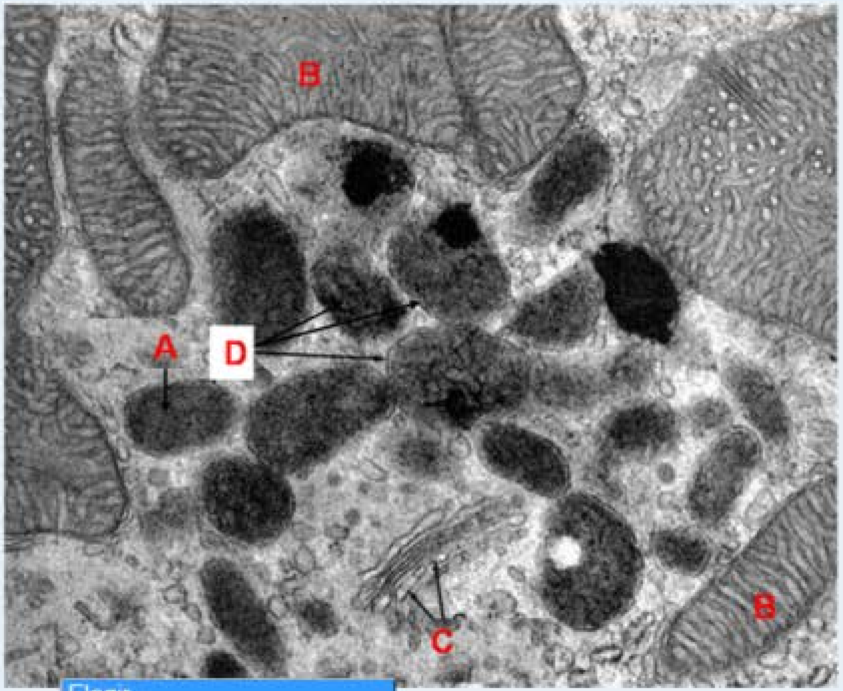

A
- Elegir...
- Aparato Golgi
- Retículo endoplásmico liso
- Mitocondrias
- Peroxisoma
- Lisosoma primario
- Lisosoma secundario

B

C Elegir...

D Elegir...

QUESTIONNAIRE I

Citology & Histology

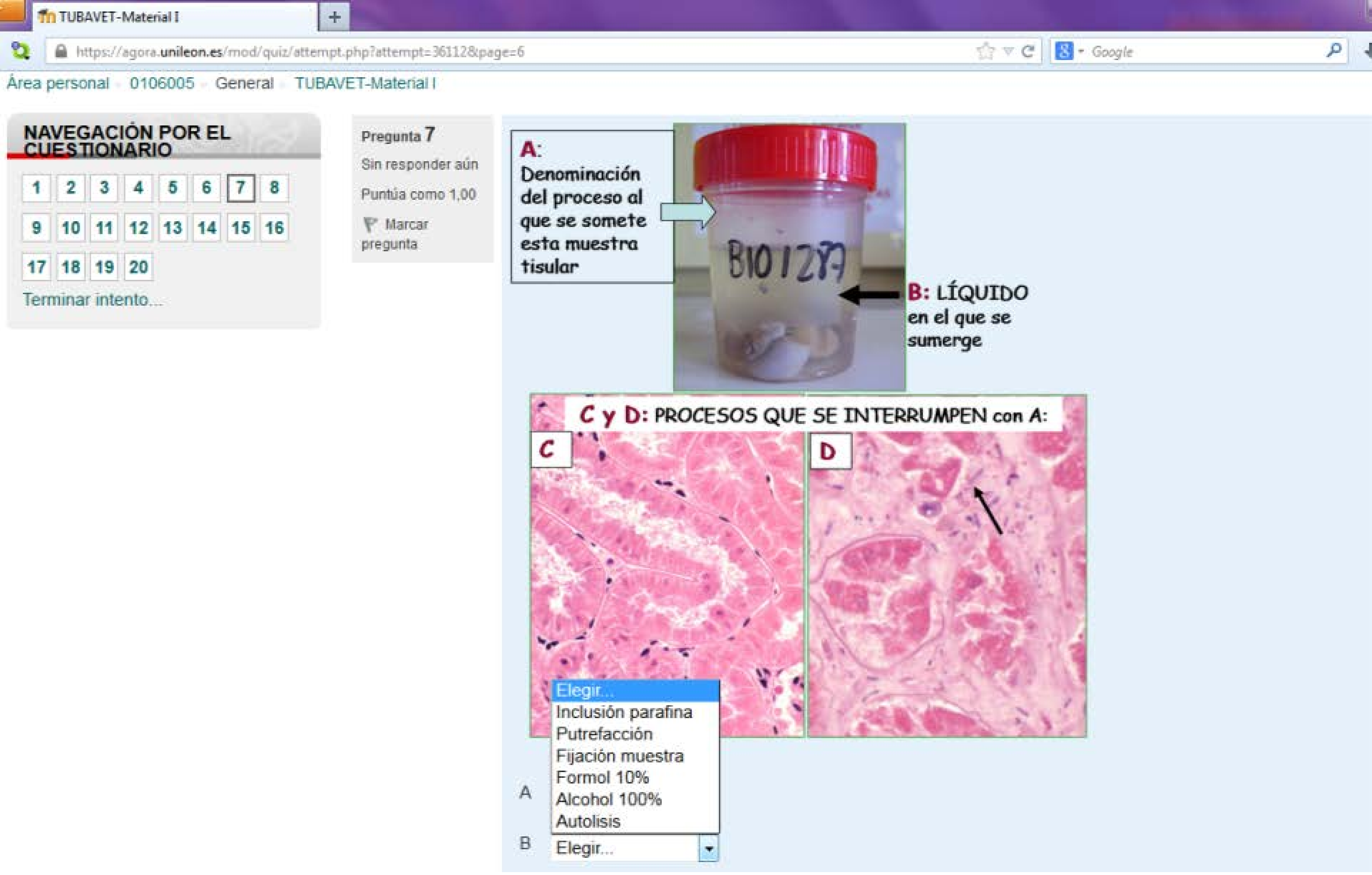

QUESTIONNAIRE I

Citology & Histology

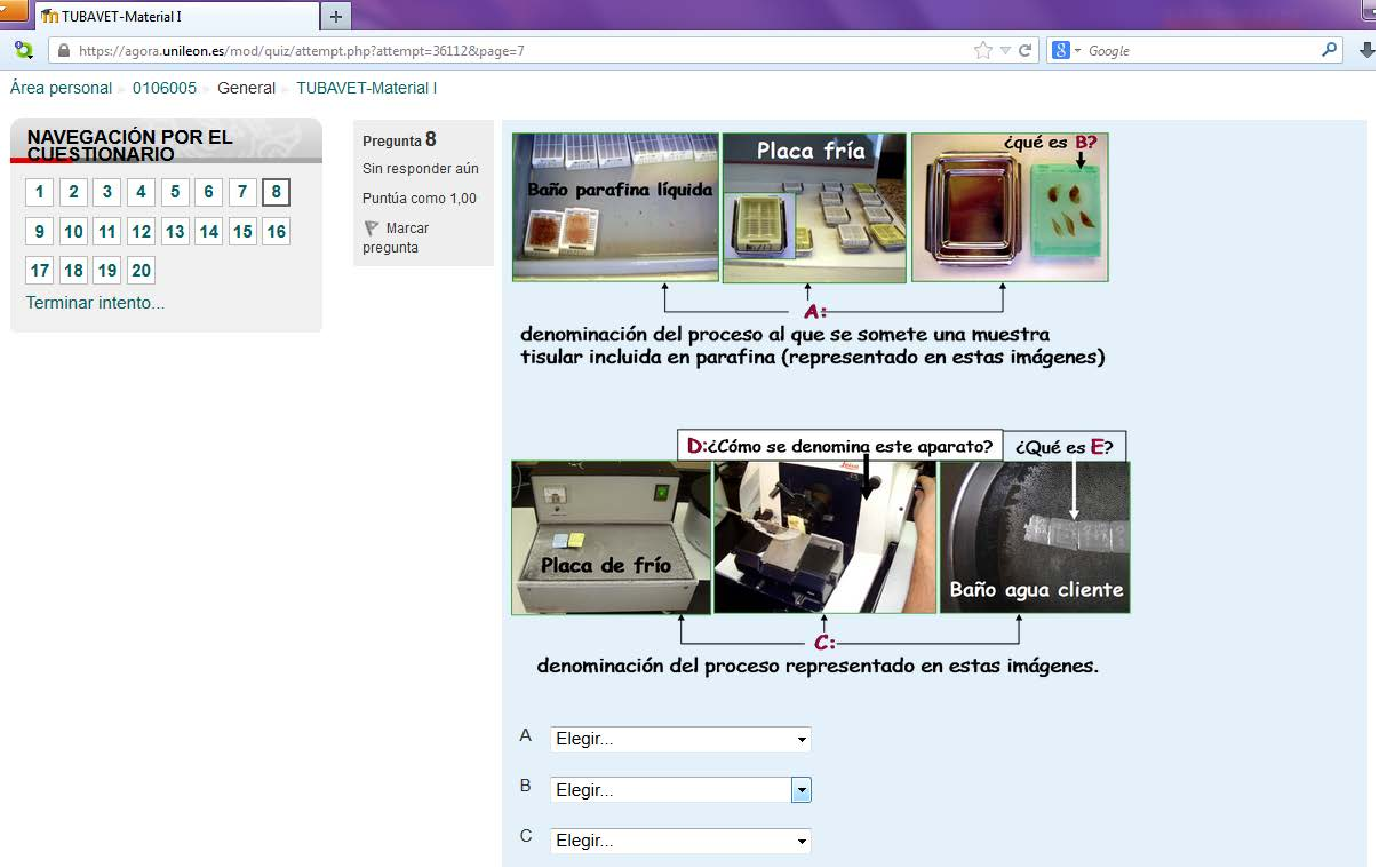

QUESTIONNAIRE I

Citology & Histology

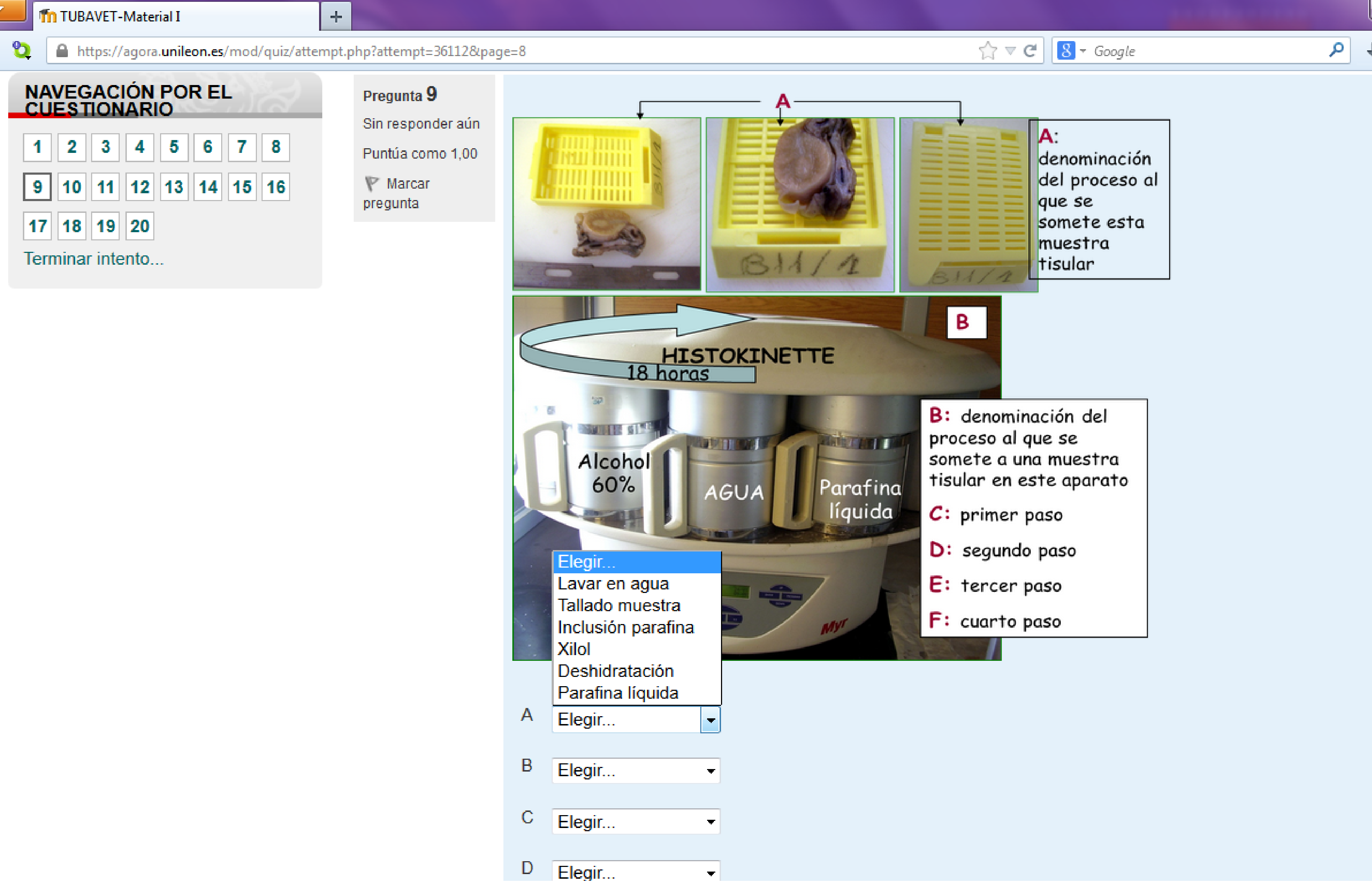

QUESTIONNAIRE I

Citology & Histology

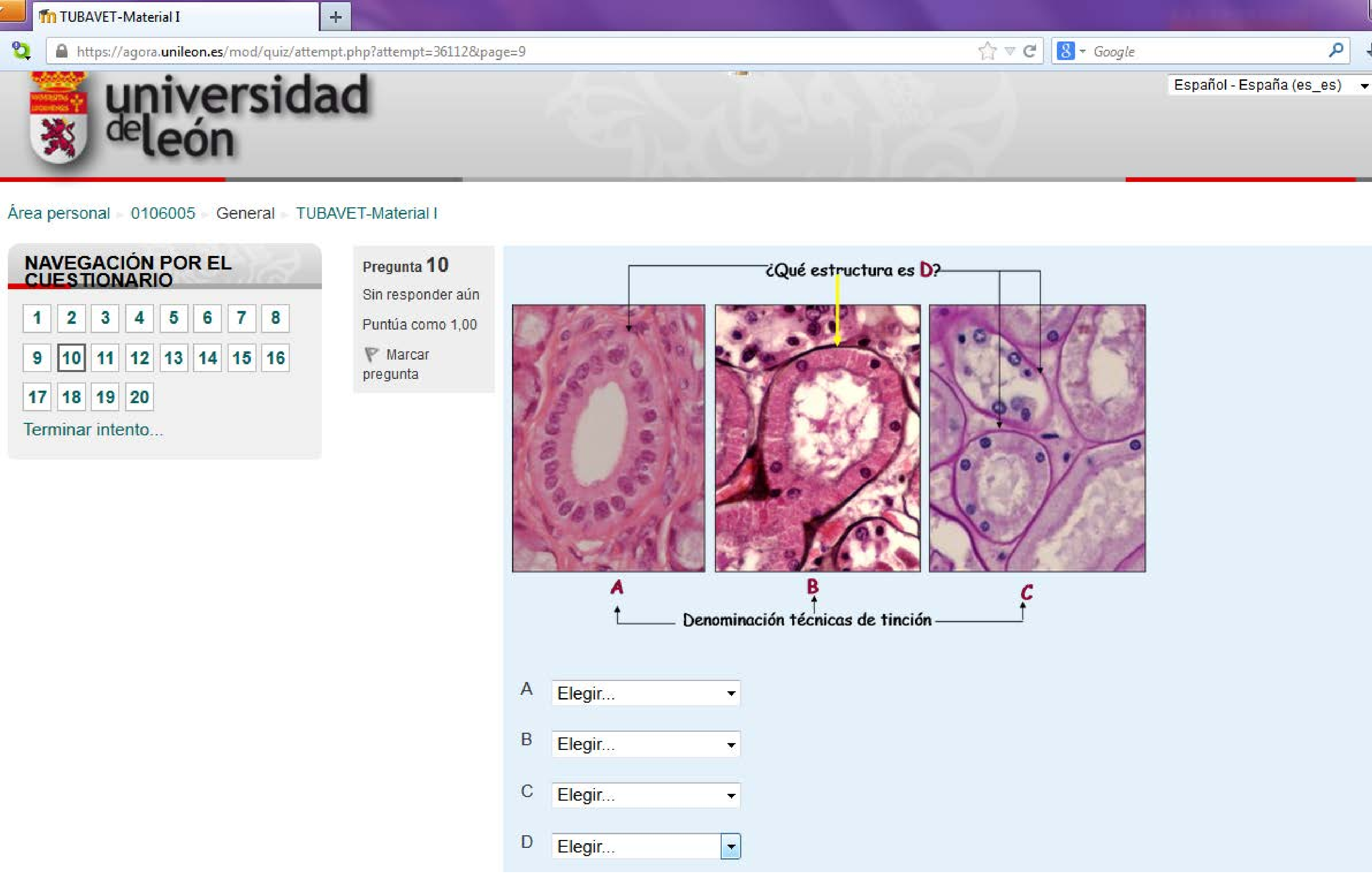

QUESTIONNAIRE I

Citology & Histology

TUBAVET-Material I

https://agora.unileon.es/mod/quiz/attempt.php?attempt=36112&page=10

universidad de león

Español - España (es_es)

Área personal ▸ 0106005 ▸ General ▸ TUBAVET-Material I

NAVEGACIÓN POR EL CUESTIONARIO

1 2 3 4 5 6 7 8
9 10 11 12 13 14 15 16
17 18 19 20

Terminar intento...

Pregunta **11**

Sin responder aún

Puntúa como 1,00

Marcar pregunta

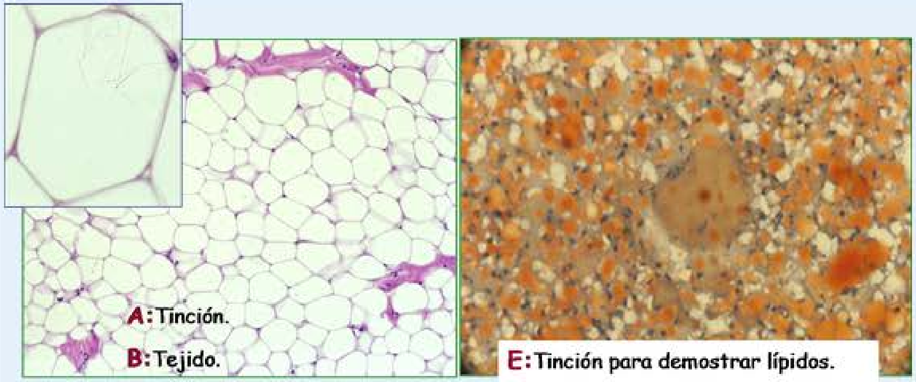

A Elegir...
- Inclusión parafina
- Hematoxilina-eosina
- Cortes tisulares por congelación
- Tejido adiposo
- Sudán
- Unilocular

B

C

D Elegir...

E Elegir...

QUESTIONNAIRE I

Citology & Histology

NAVEGACIÓN POR EL CUESTIONARIO

1 2 3 4 5 6 7 8
9 10 11 12 13 14 15 16
17 18 19 20

Terminar intento...

Pregunta 12

Sin responder aún

Puntúa como 1,00

Marcar pregunta

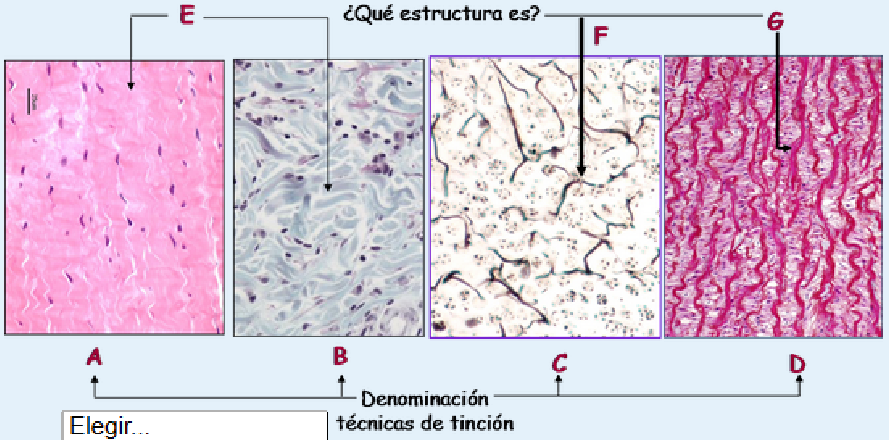

Elegir...
Fibras reticulina
Fibras elásticas
Orceina
Tricrómico Masson
Fibras colágenas
Hematoxilina-eosina
Fibras musculares
Tinción plata
PAS

A
B
C
D Elegir...
E Elegir...
F Elegir...
G Elegir...

QUESTIONNAIRE I

Citology & Histology

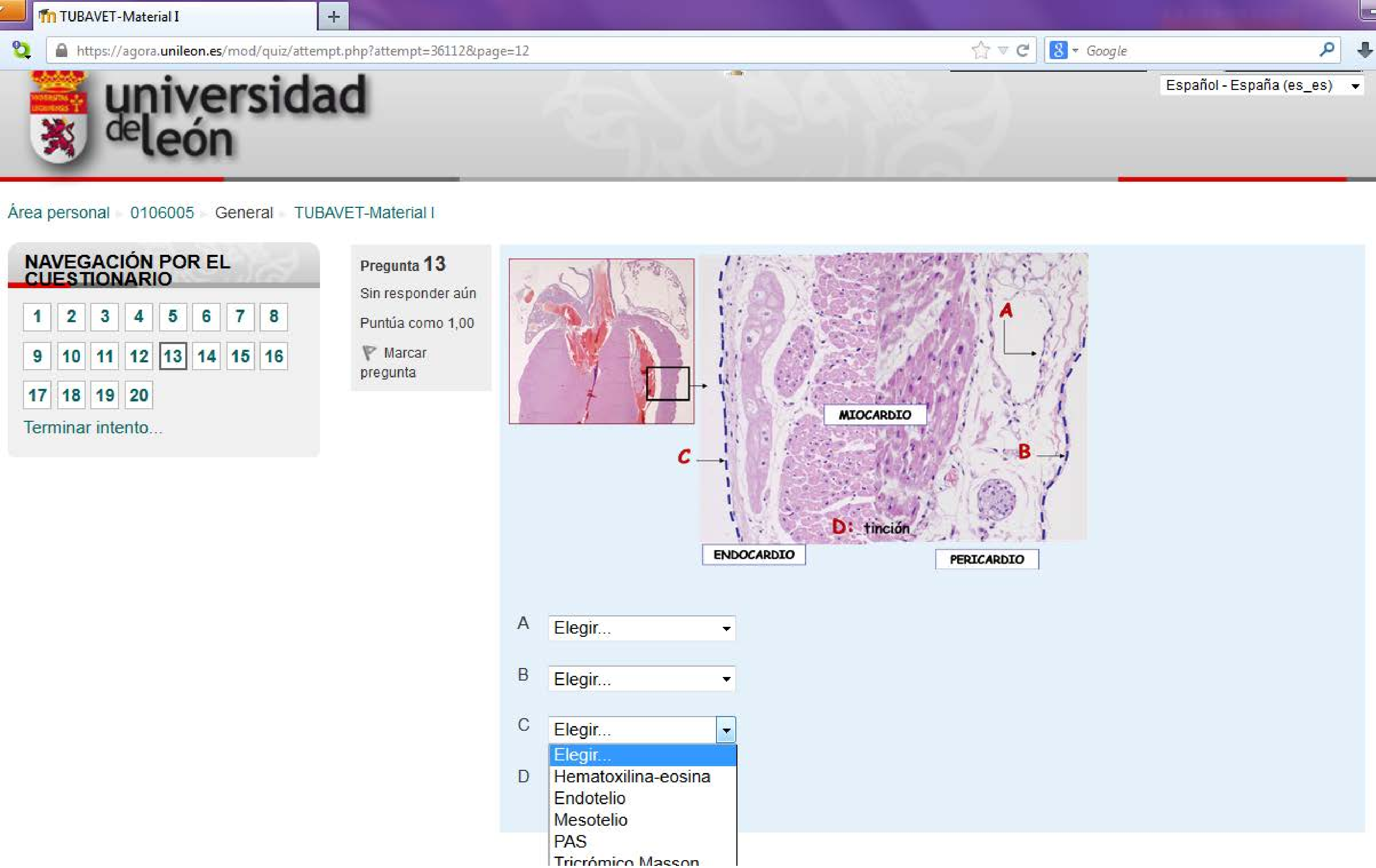

QUESTIONNAIRE I

Citology & Histology

TUBAVET-Material I

https://agora.unileon.es/mod/quiz/attempt.php?attempt=36112&page=13

universidad de león

Español - España (es_es)

Área personal ▸ 0106005 ▸ General ▸ TUBAVET-Material I

NAVEGACIÓN POR EL CUESTIONARIO

1 2 3 4 5 6 7 8
9 10 11 12 13 14 15 16
17 18 19 20

Terminar intento...

Pregunta 14

Sin responder aún

Puntúa como 1,00

Marcar pregunta

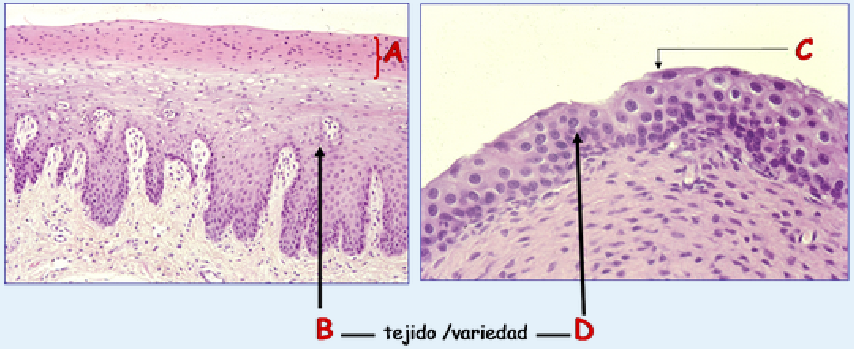

A Elegir...

B Elegir...

C Elegir...

D Elegir...

- Elegir...
- Estrato funcional superficial
- Epitelio seudoestratificado ciliado
- Epitelio plano estratificado mucoso
- Epitelio transición
- Célula superficial

Siguiente

QUESTIONNAIRE I

Citology & Histology

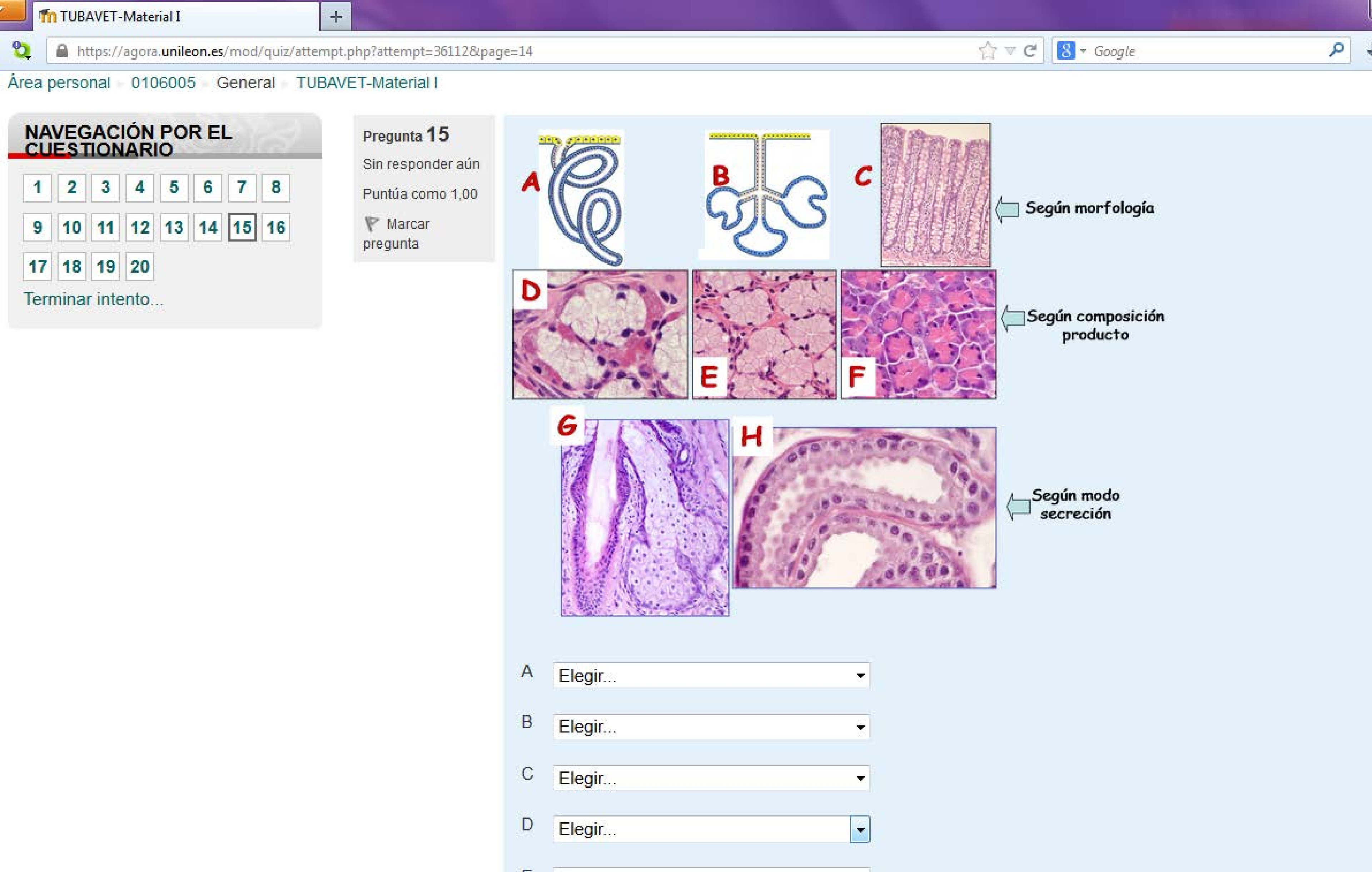

QUESTIONNAIRE I

Citology & Histology

NAVEGACIÓN POR EL CUESTIONARIO
1 2 3 4 5 6 7 8
9 10 11 12 13 14 15 16
17 18 19 20
Terminar intento...

Pregunta 16

Sin responder aún

Puntúa como 1,00

Marcar pregunta

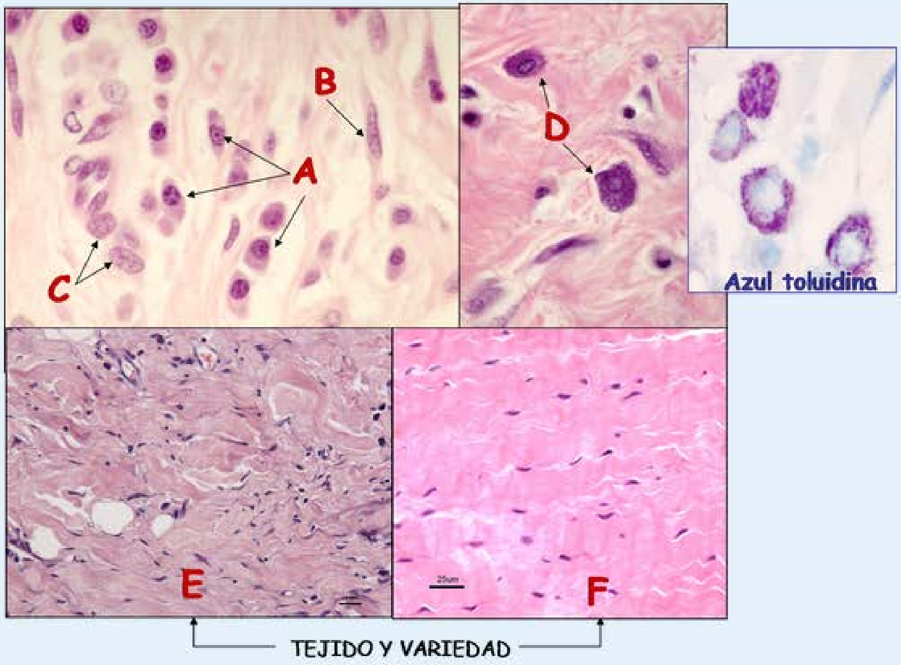

A Elegir...

B Elegir...
- Fibroblasto
- Tejido conjuntivo denso no orientado
- Mastocitos
- Histiocitos
- Células plasmáticas
- Tejido conjuntivo denso orientado

C

D

E Elegir...

QUESTIONNAIRE I

Citology & Histology

TUBAVET-Material I

https://agora.unileon.es/mod/quiz/attempt.php?attempt=36112&page=16

Área personal ▸ 0106005 ▸ General ▸ TUBAVET-Material I

NAVEGACIÓN POR EL CUESTIONARIO

1 2 3 4 5 6 7 8
9 10 11 12 13 14 15 16
17 18 19 20

Terminar intento...

Pregunta 17

Sin responder aún

Puntúa como 1,00

Marcar pregunta

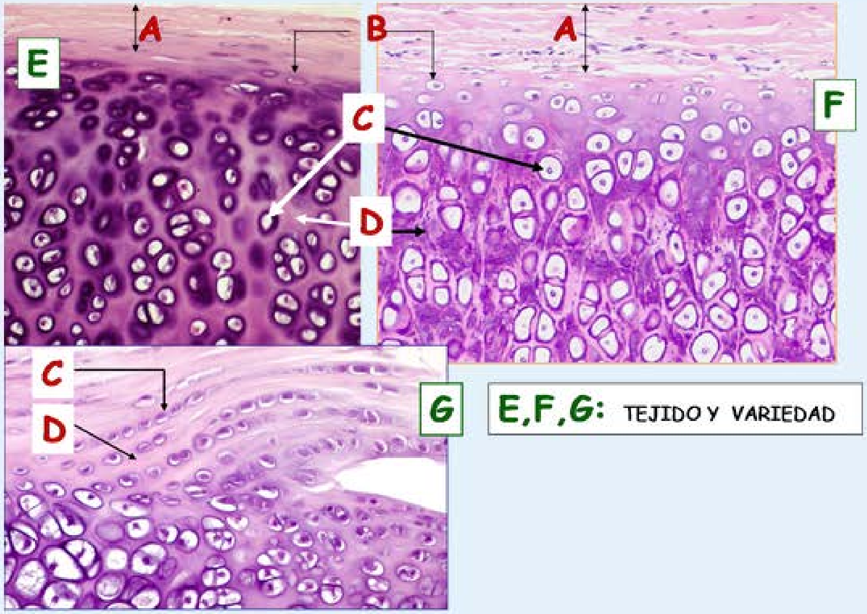

A Elegir...

B Elegir...

C Elegir...

D Elegir...

E Elegir...

QUESTIONNAIRE I

Citology & Histology

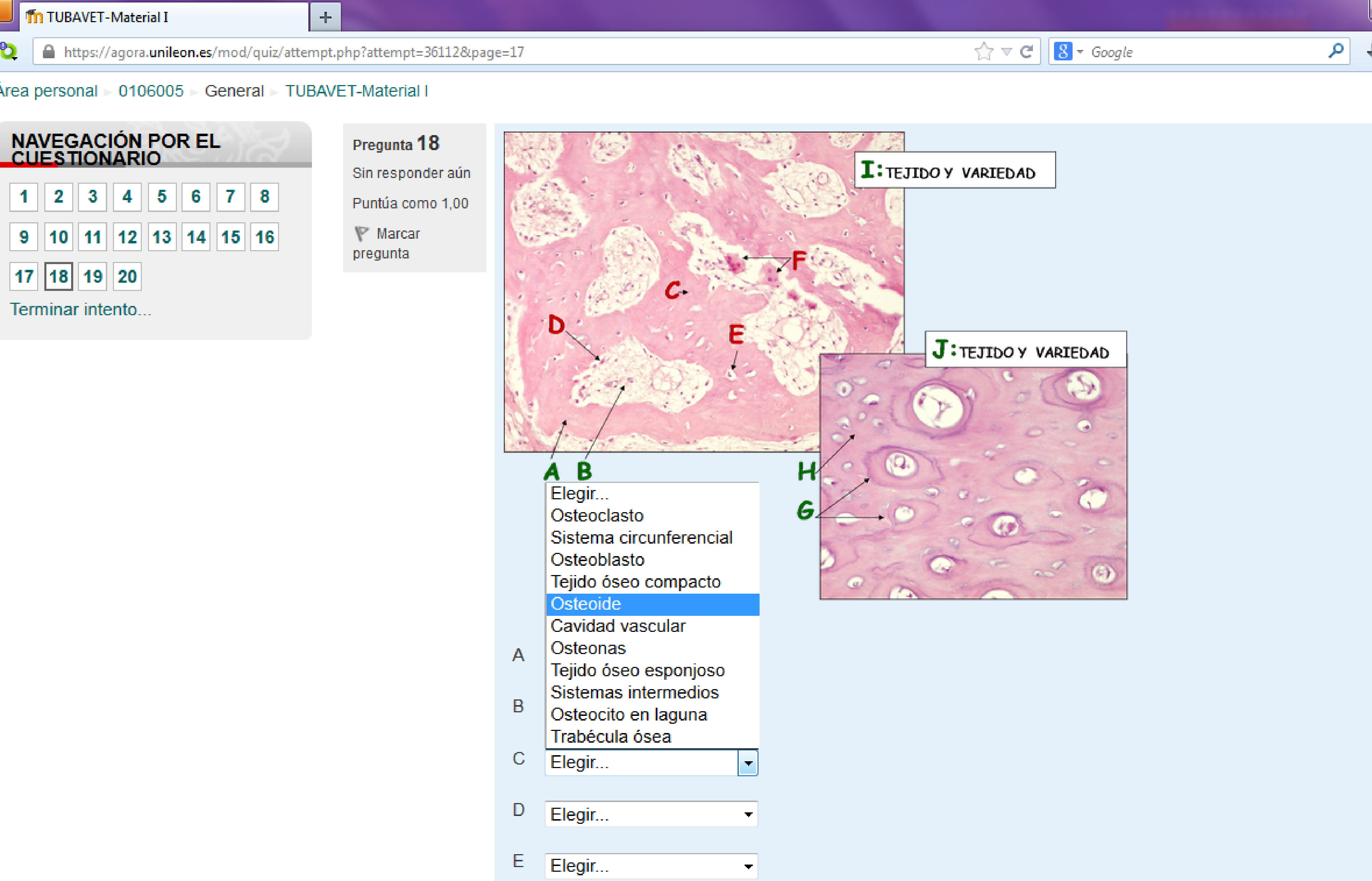

QUESTIONNAIRE I

Citology & Histology

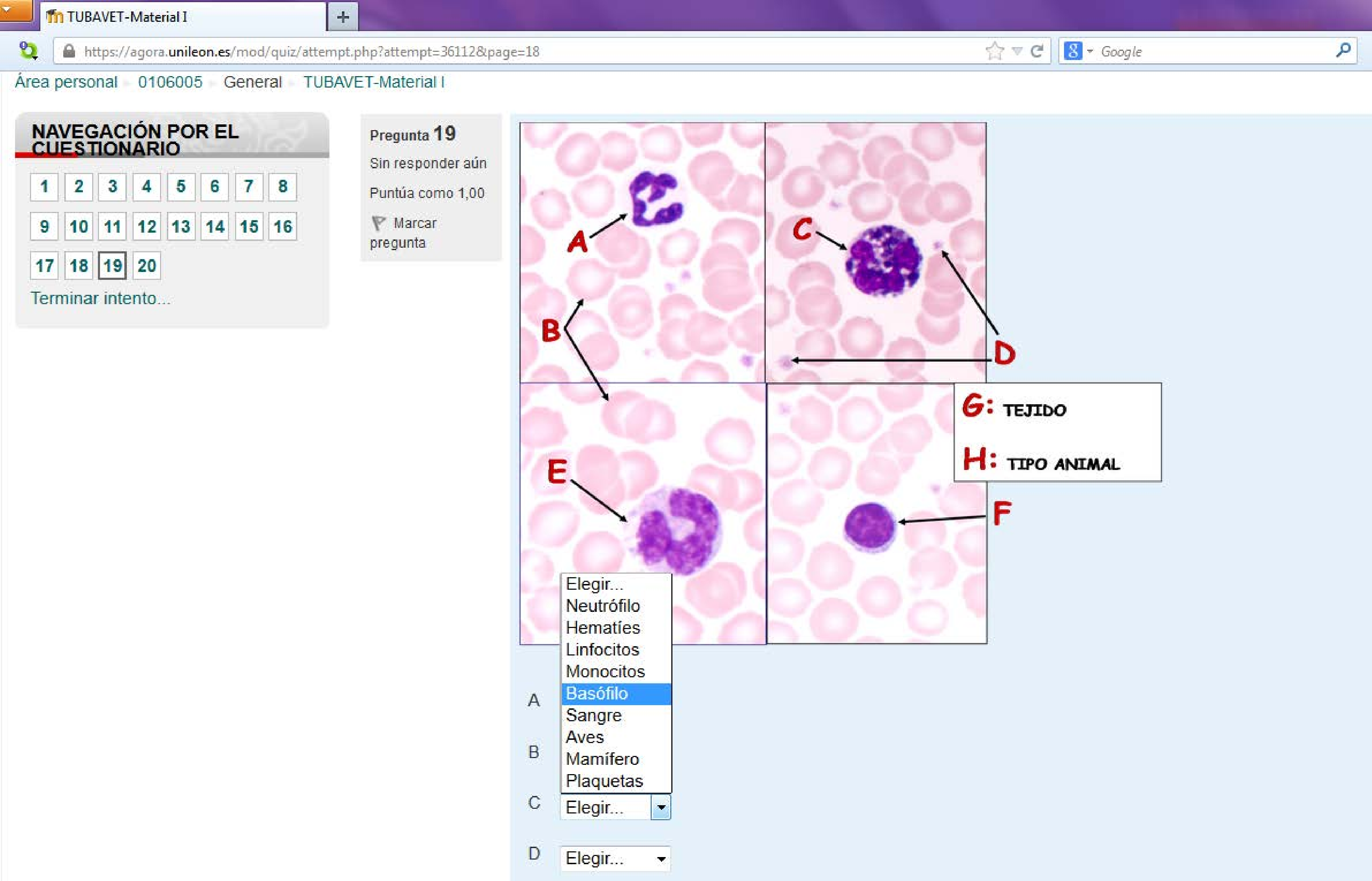

QUESTIONNAIRE I

Citology & Histology

TUBAVET-Material I

https://agora.unileon.es/mod/quiz/attempt.php?attempt=36112&page=19

Área personal ▸ 0106005 ▸ General ▸ TUBAVET-Material I

NAVEGACIÓN POR EL CUESTIONARIO

1 2 3 4 5 6 7 8
9 10 11 12 13 14 15 16
17 18 19 20

Terminar intento...

Pregunta **20**

Sin responder aún

Puntúa como 1,00

Marcar pregunta

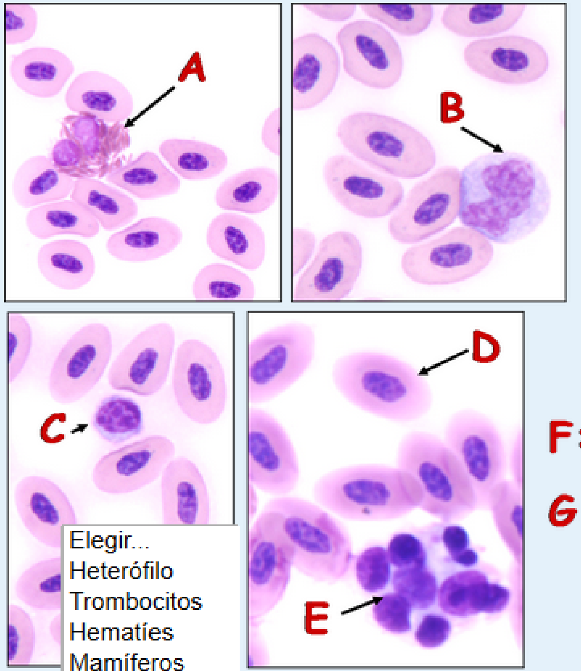

F: TEJIDO

G: TIPO ANIMAL

A

Elegir...
Heterófilo
Trombocitos
Hematíes
Mamíferos
Linfocito
Monocito
Aves
Sangre

B Elegir...

C Elegir...

D Elegir...

E Elegir...

QUESTIONNAIRE I

Citology & Histology

¿A QUÉ PARTE DEL OJO CORRESPONDE LA IMAGEN?

1

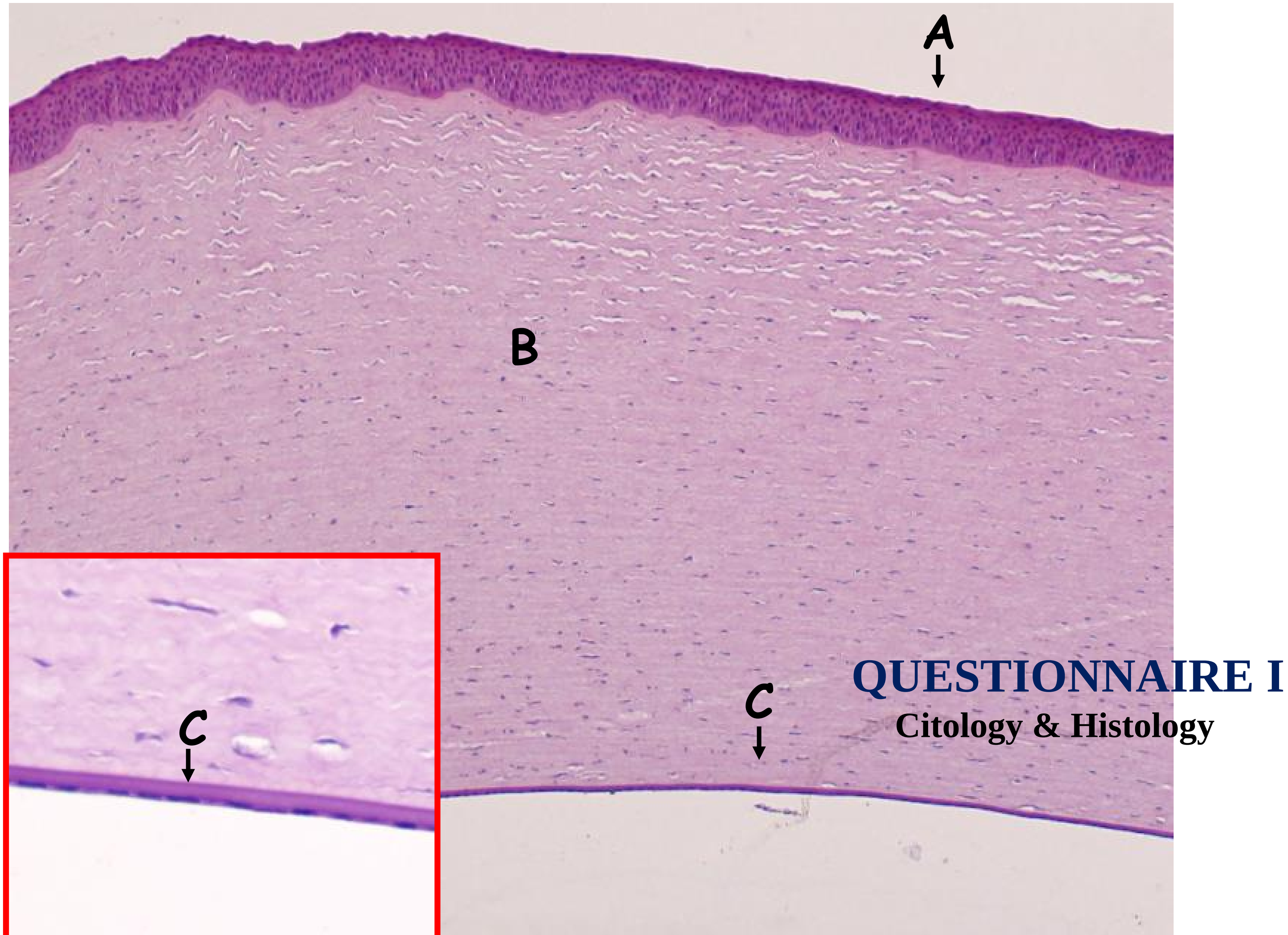

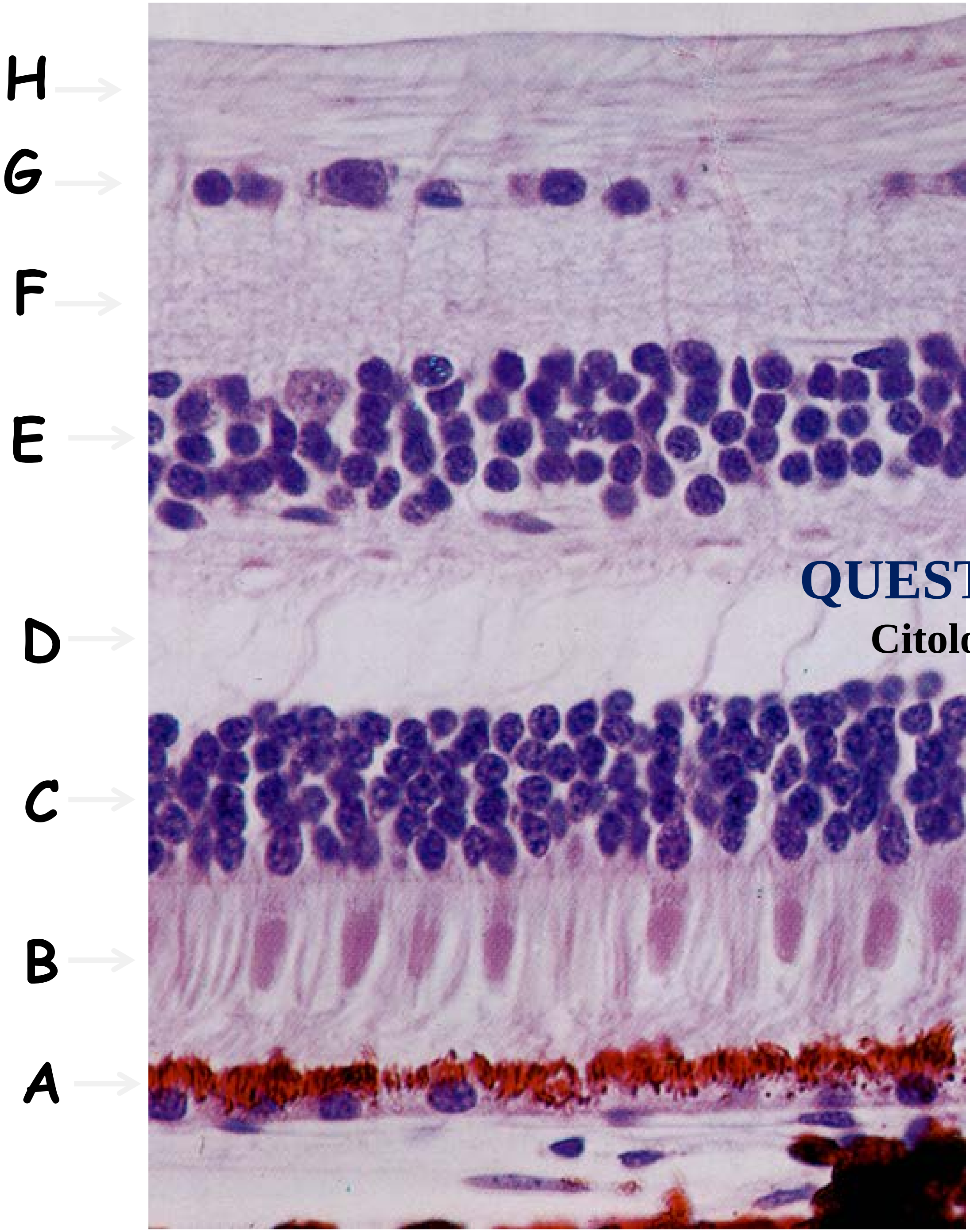

QUESTIONNAIRE II

Citology & Histology

¿QUÉ ÓRGANO APARECE EN LA IMAGEN?

¿QUÉ ES A, B, C?

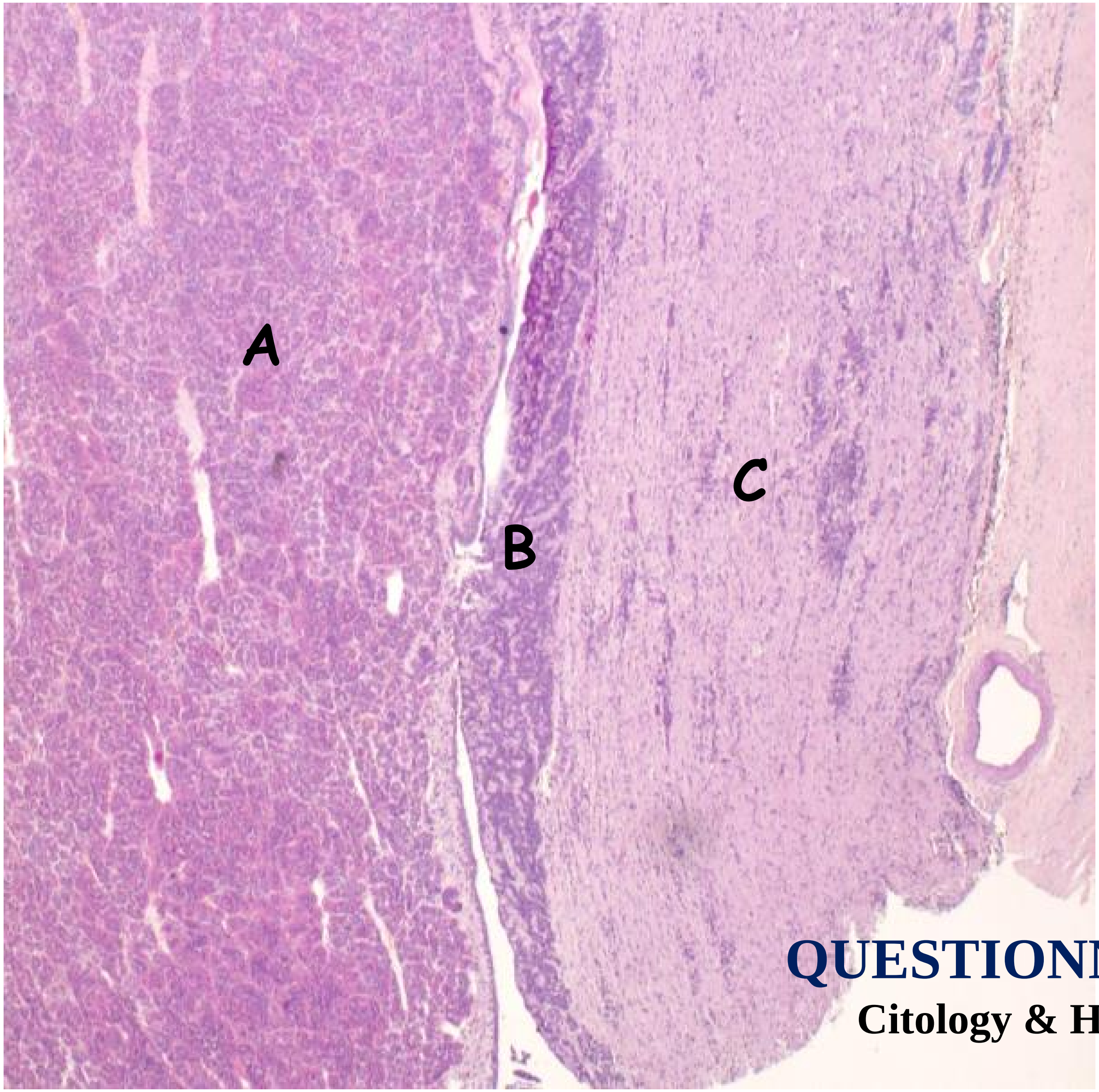

¿QUÉ ÓRGANO APARECE EN LA IMAGEN?

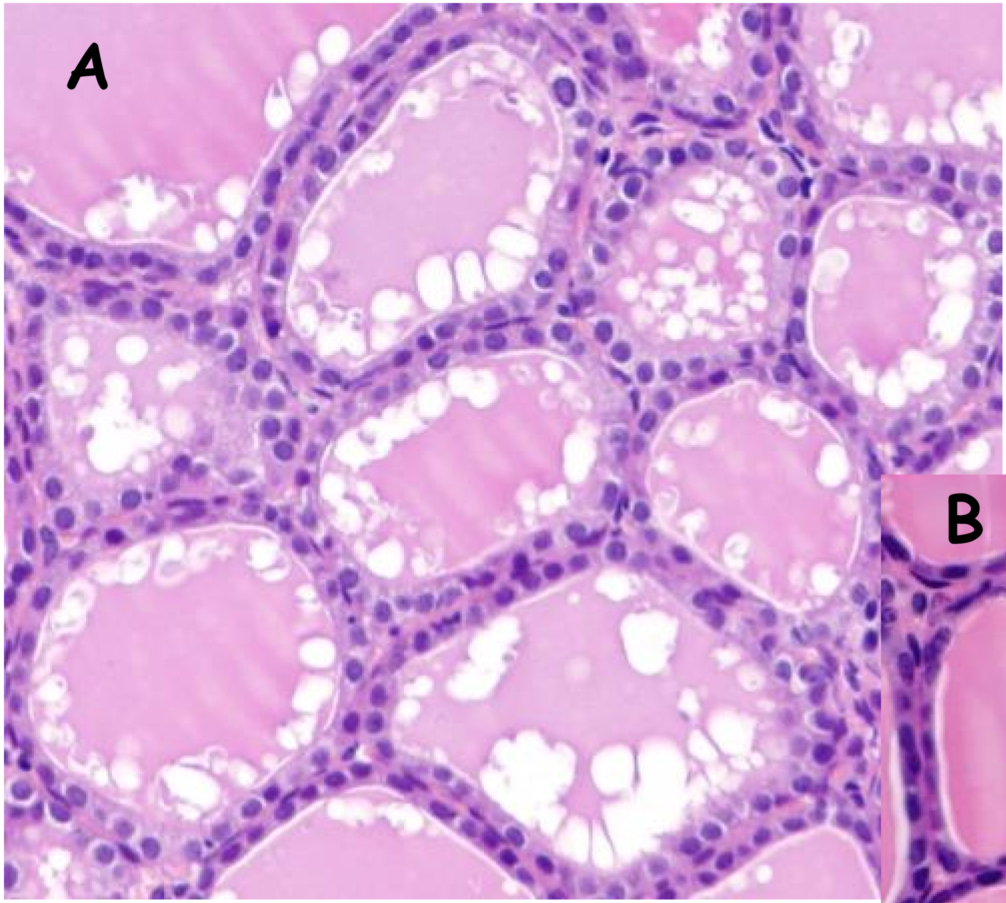

ESTADO FUNCIONAL
EN A y B

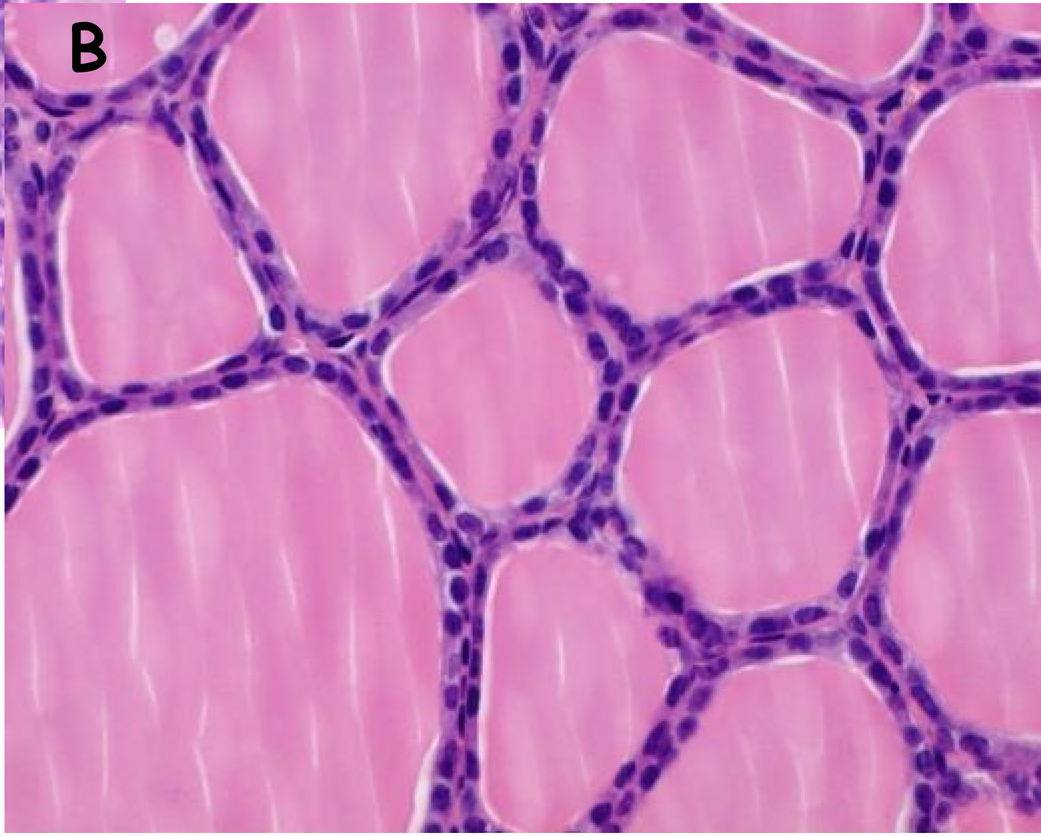

QUESTIONNAIRE II

Citology & Histology

¿QUÉ ÓRGANO APARECE EN LA IMAGEN?

¿QUÉ ES A, B y C?

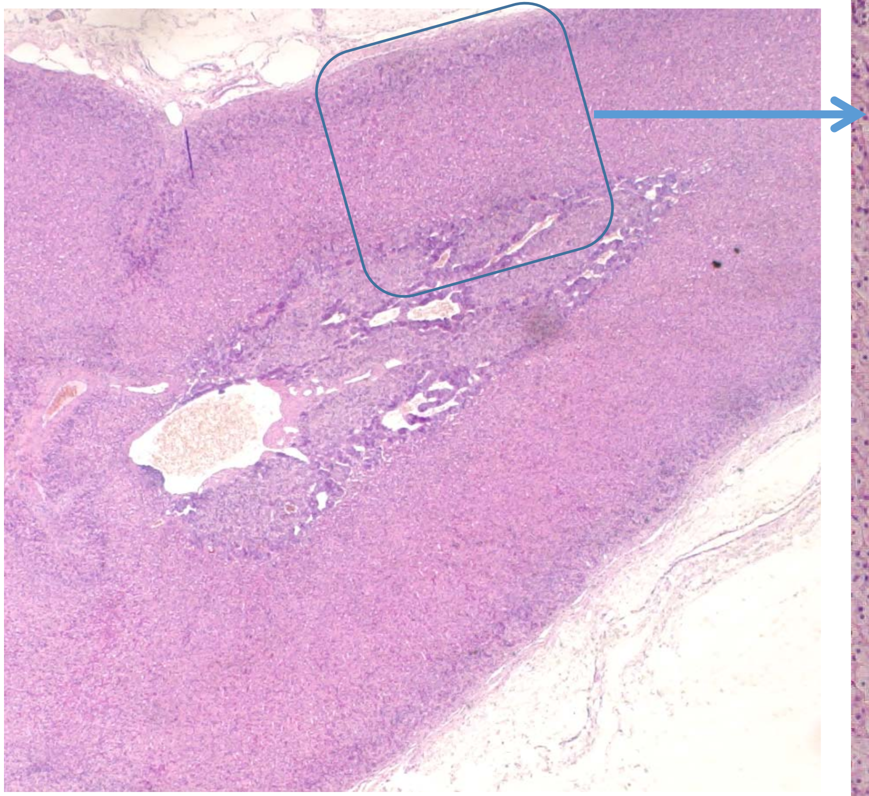

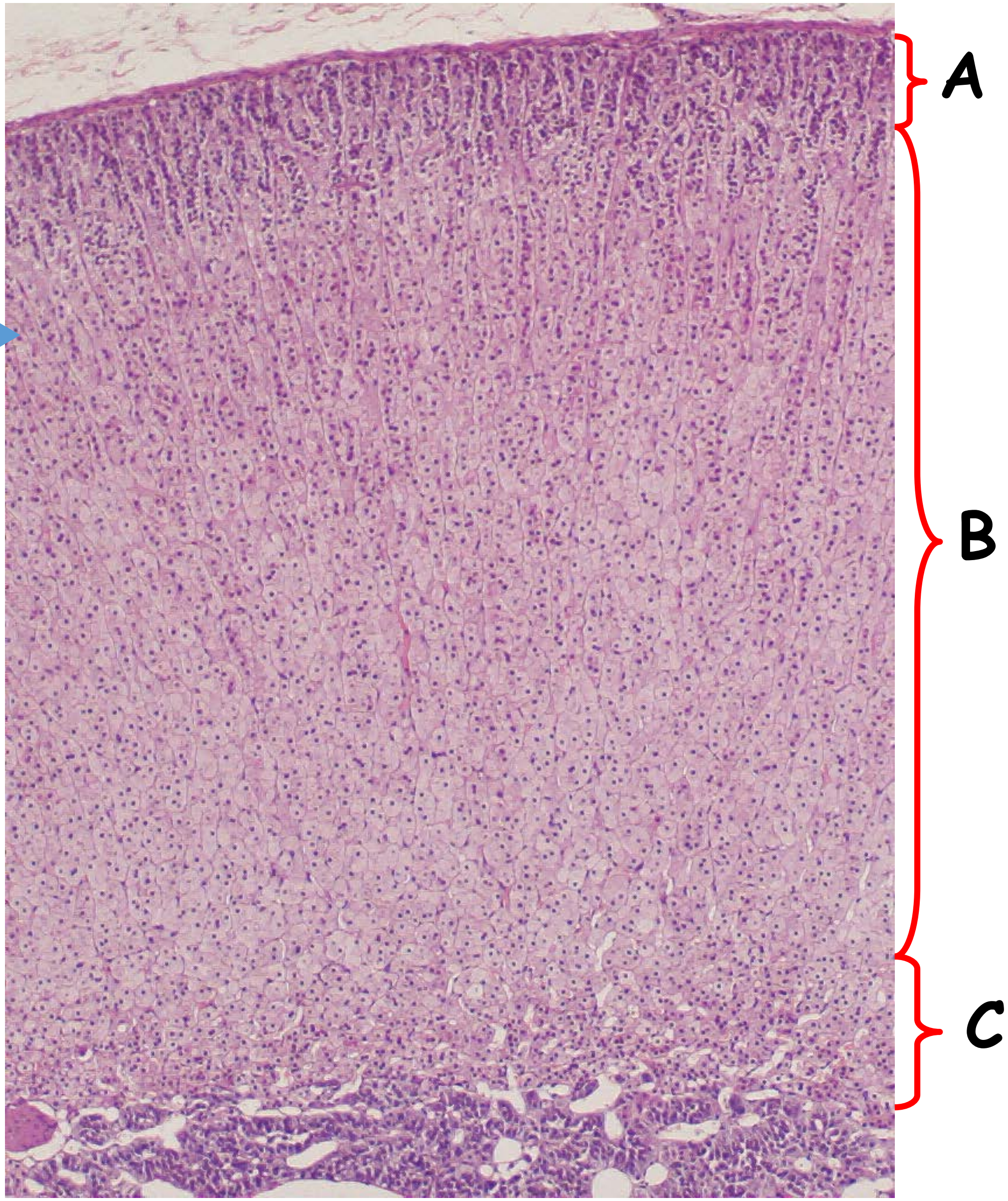

QUESTIONNAIRE II

Citology & Histology

¿QUÉ ÓRGANO APARECE EN LA IMAGEN?

6

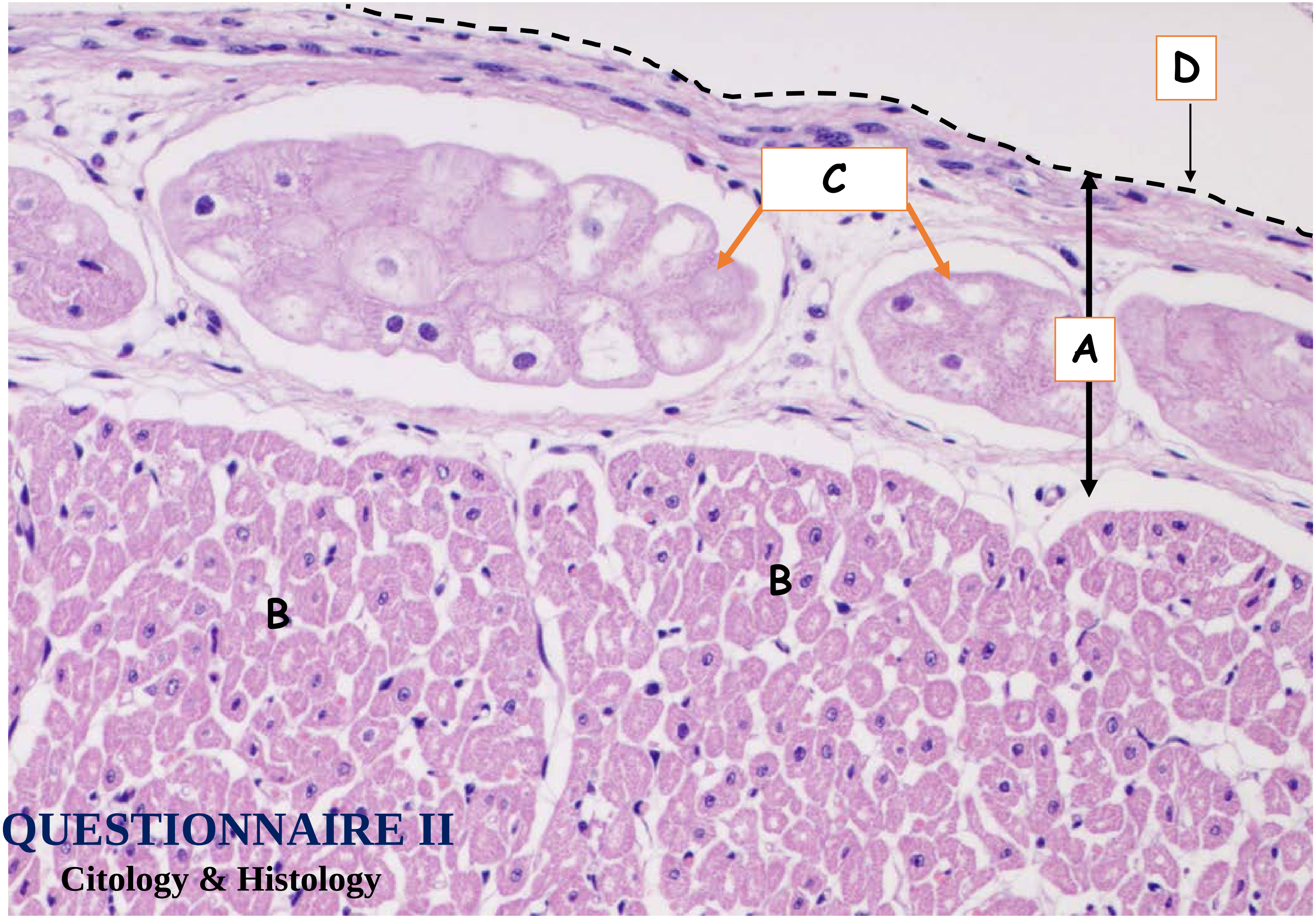

H-E.

ÓRGANO Y VARIEDAD

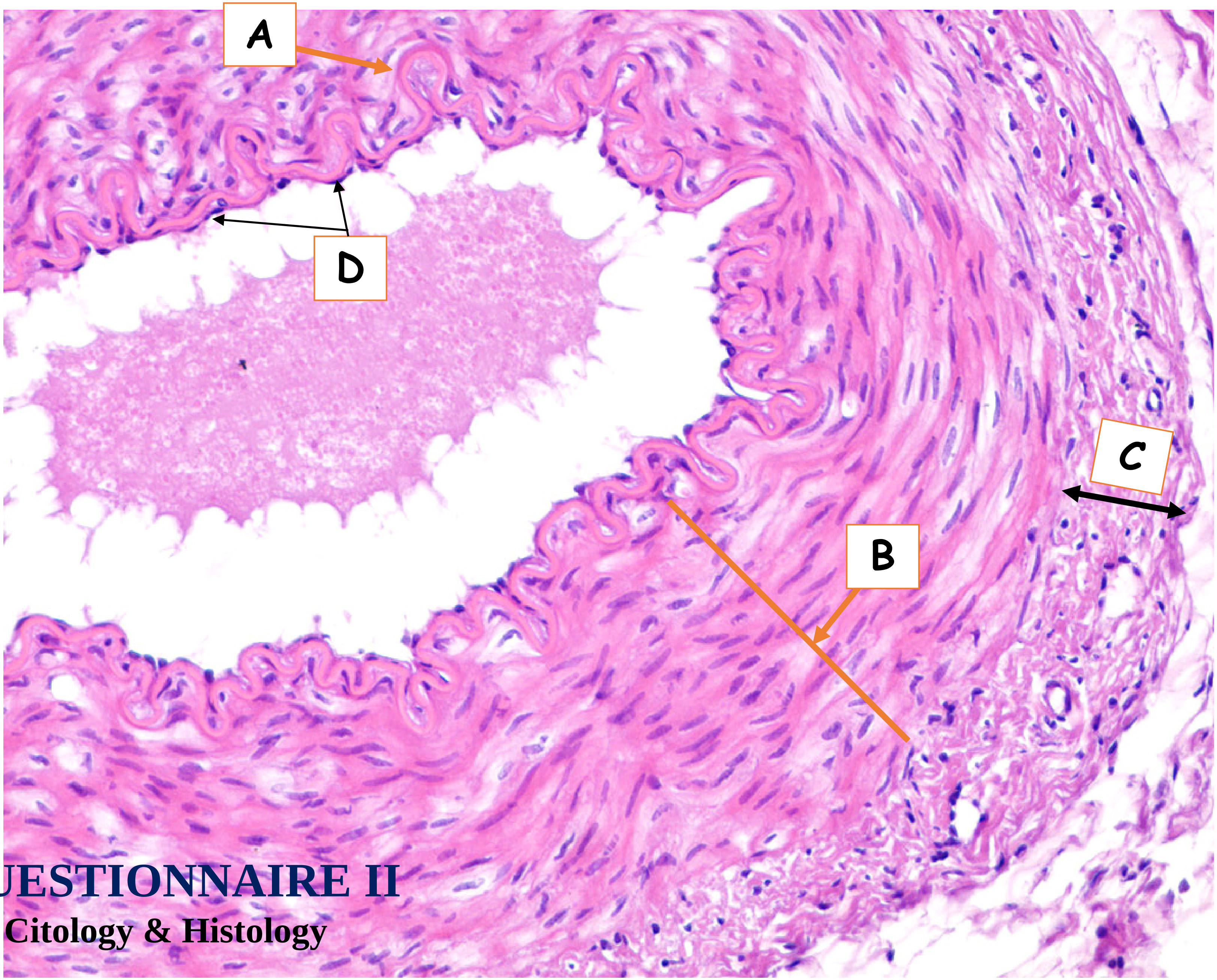

QUESTIONNAIRE II

Citology & Histology

ÓRGANO Y VARIEDAD

QUESTIONNAIRE II

Citology & Histology

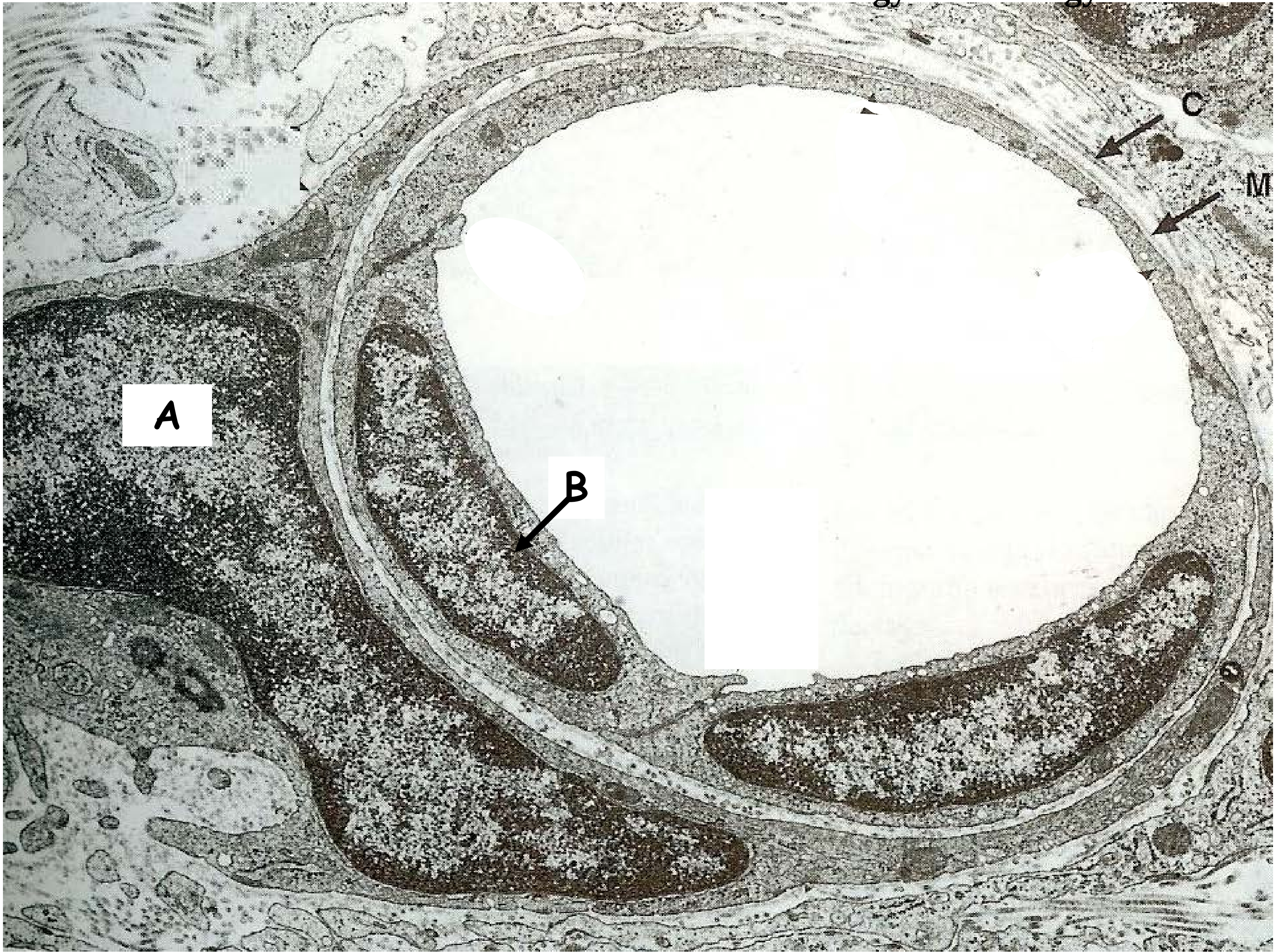

ÓRGANO Y VARIEDAD

QUESTIONNAIRE II

Citology & Histology

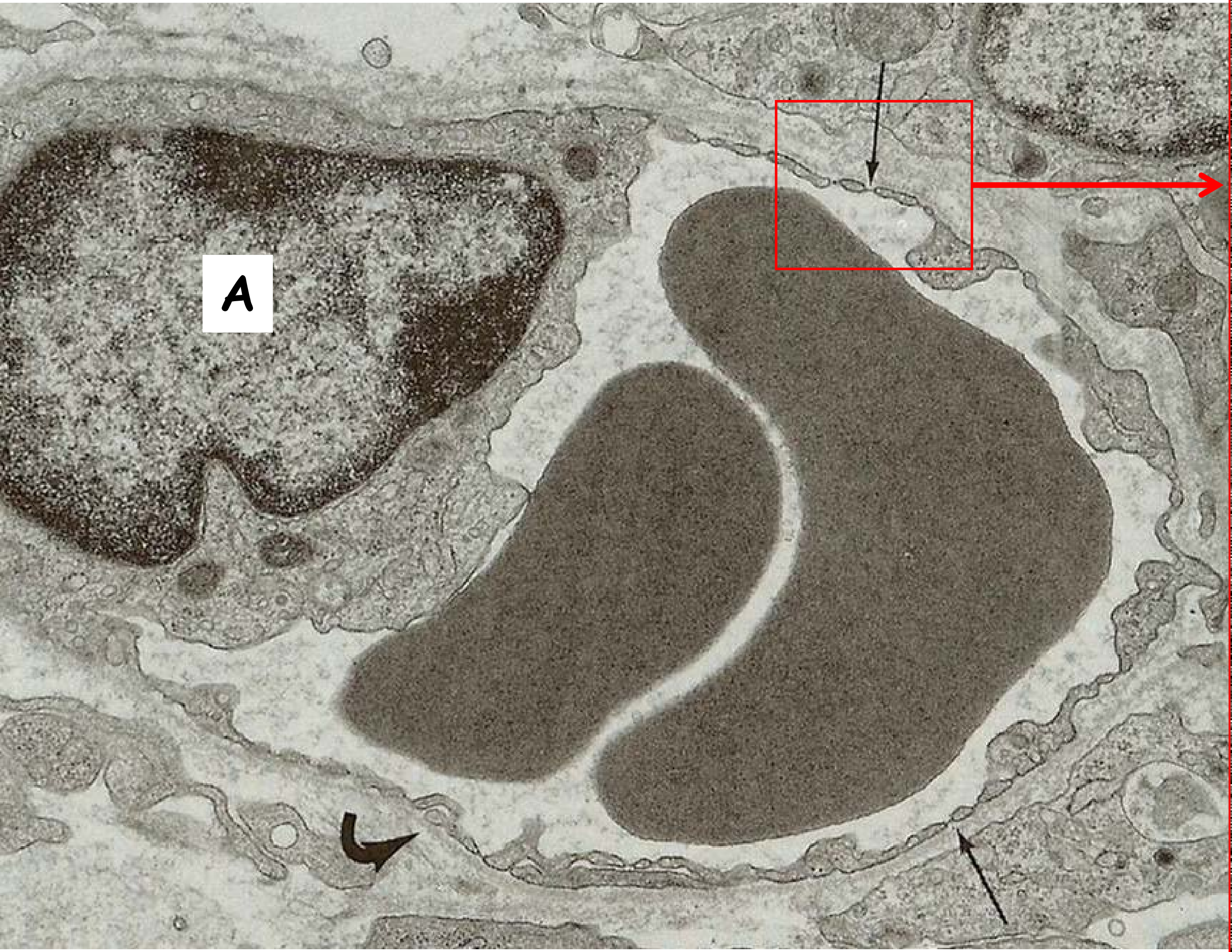

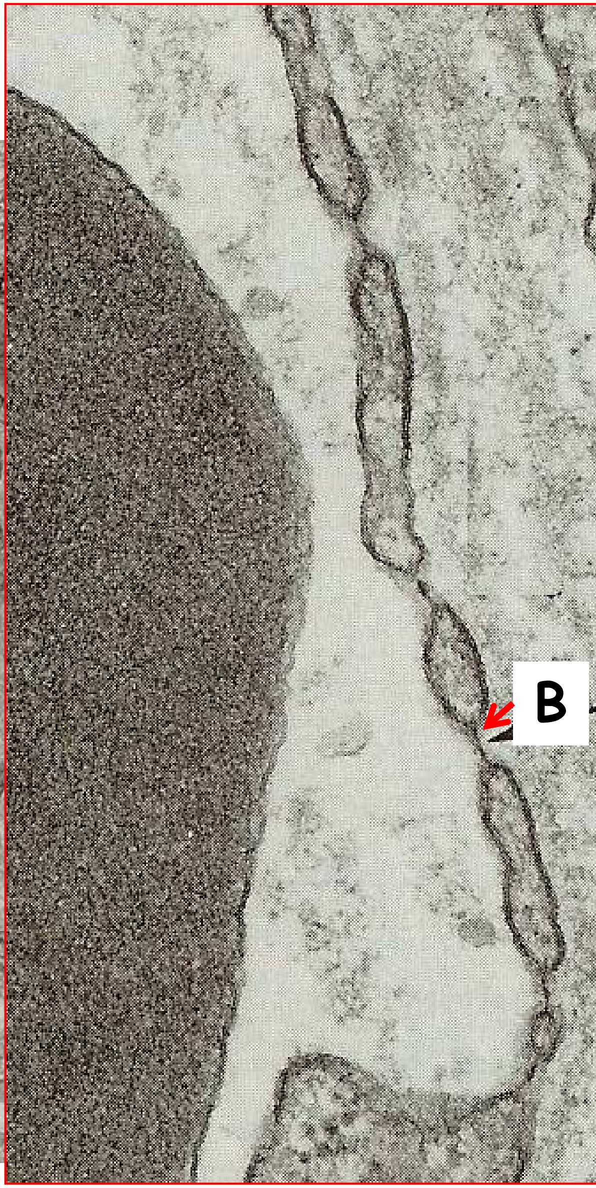

ÓRGANO

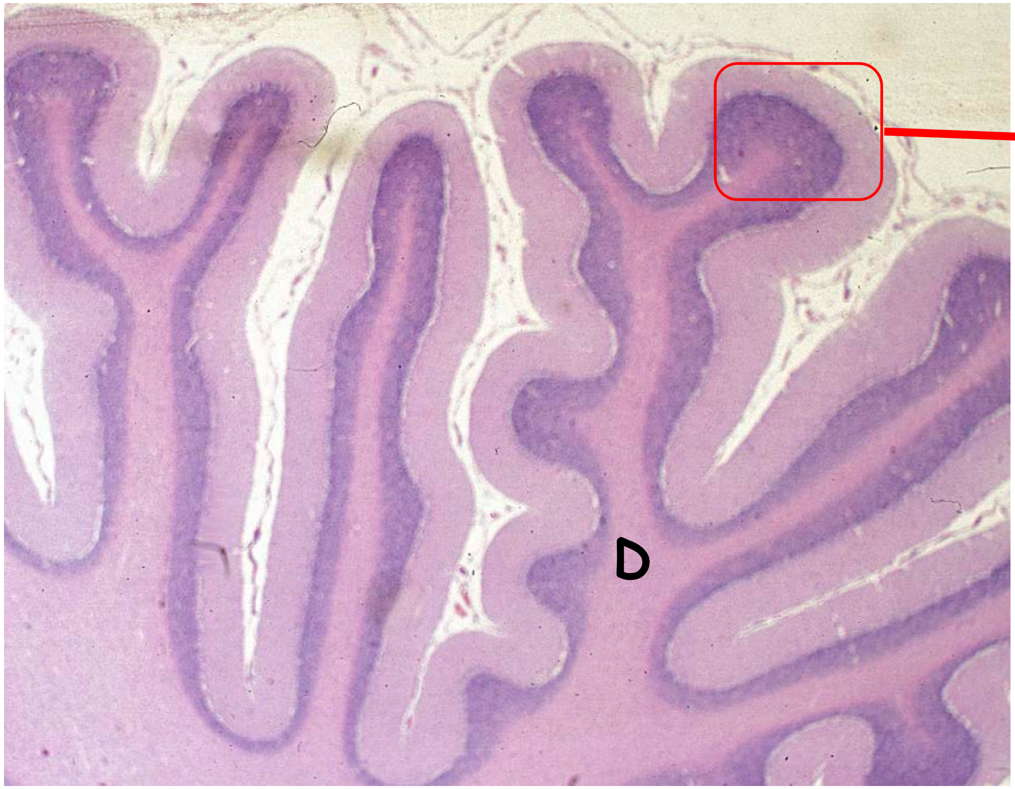

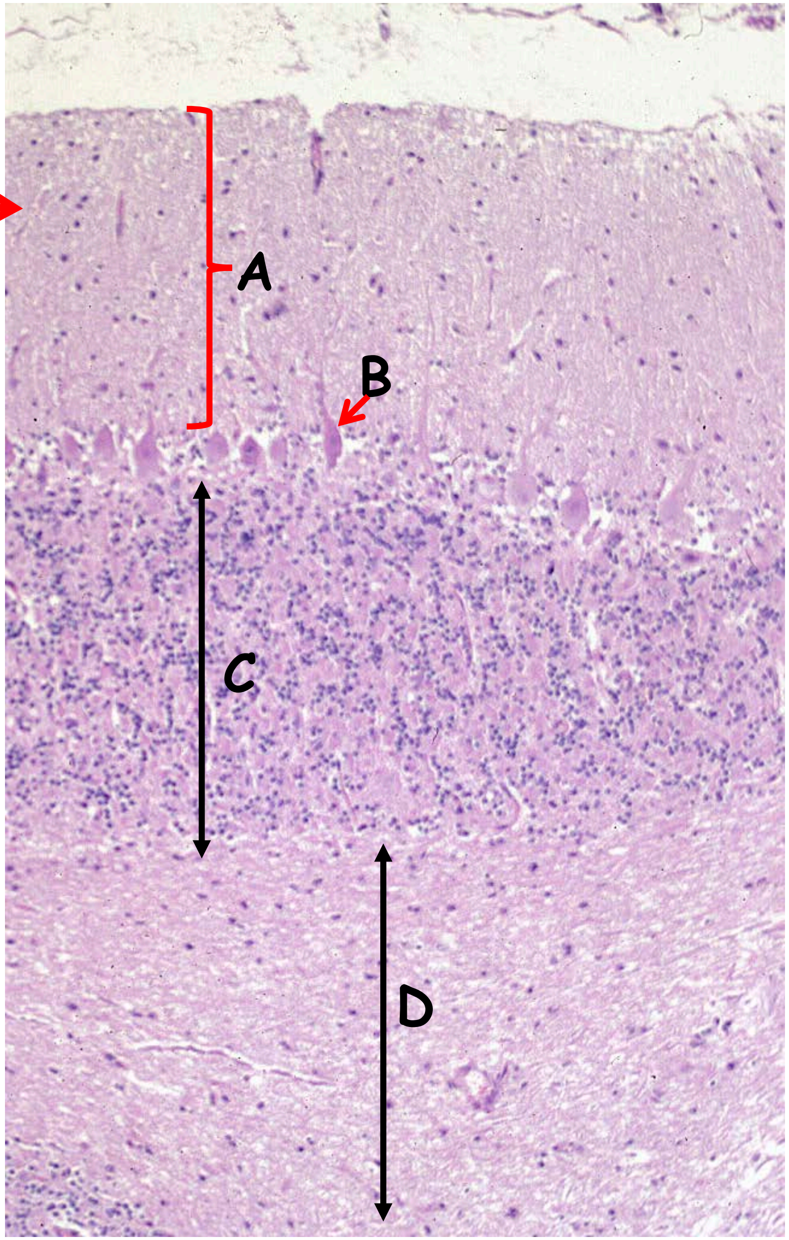

QUESTIONNAIRE II

Citology & Histology

QUESTIONNAIRE II

Citology & Histology

ÓRGANO

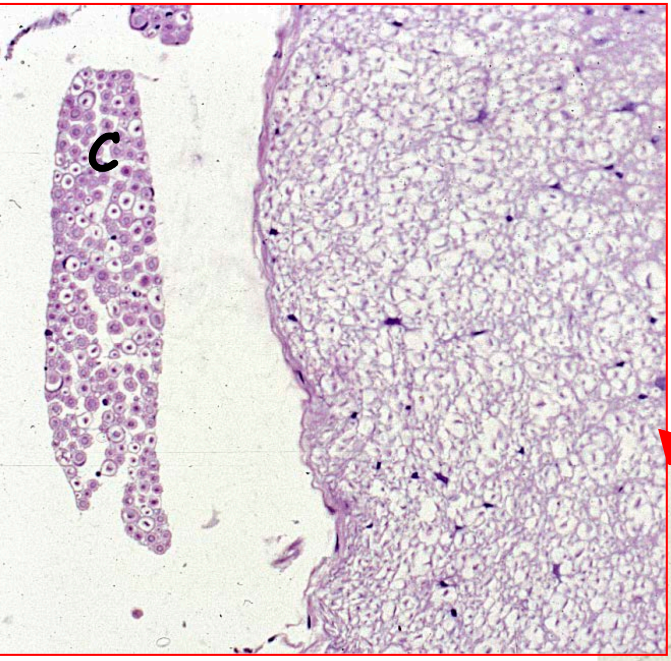

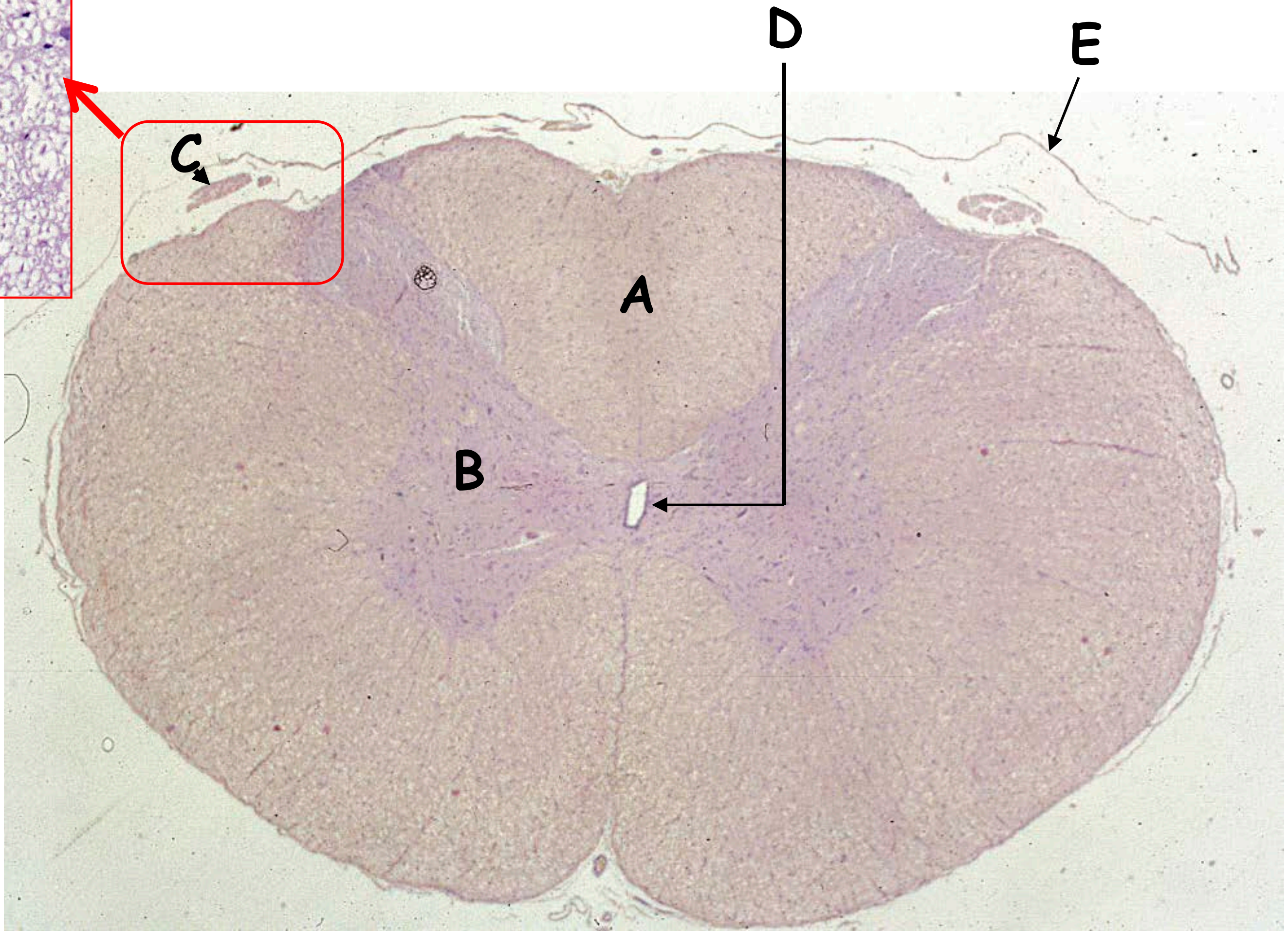

ÓRGANO, ESPECIE

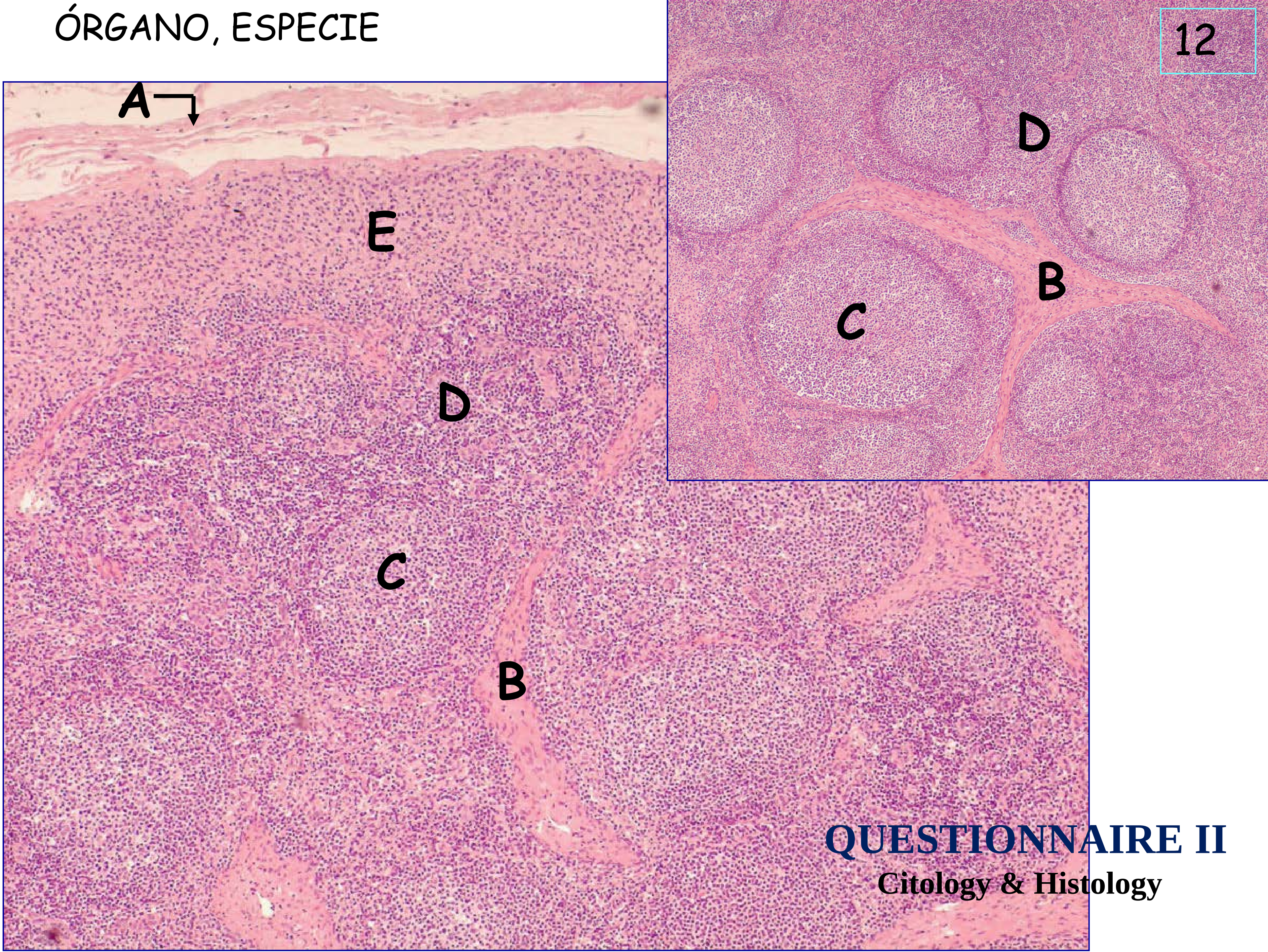

ÓRGANO

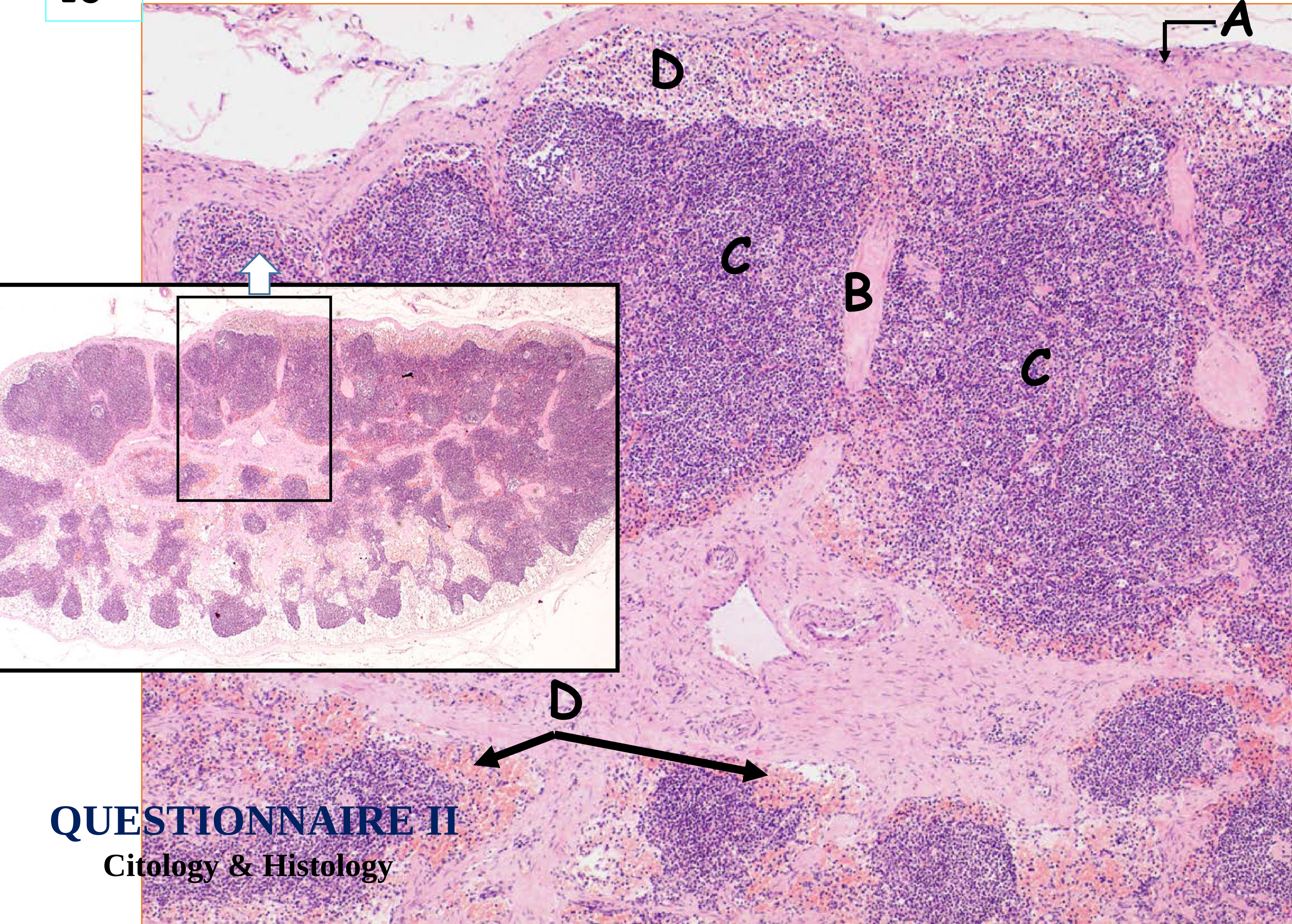

QUESTIONNAIRE II

Citology & Histology

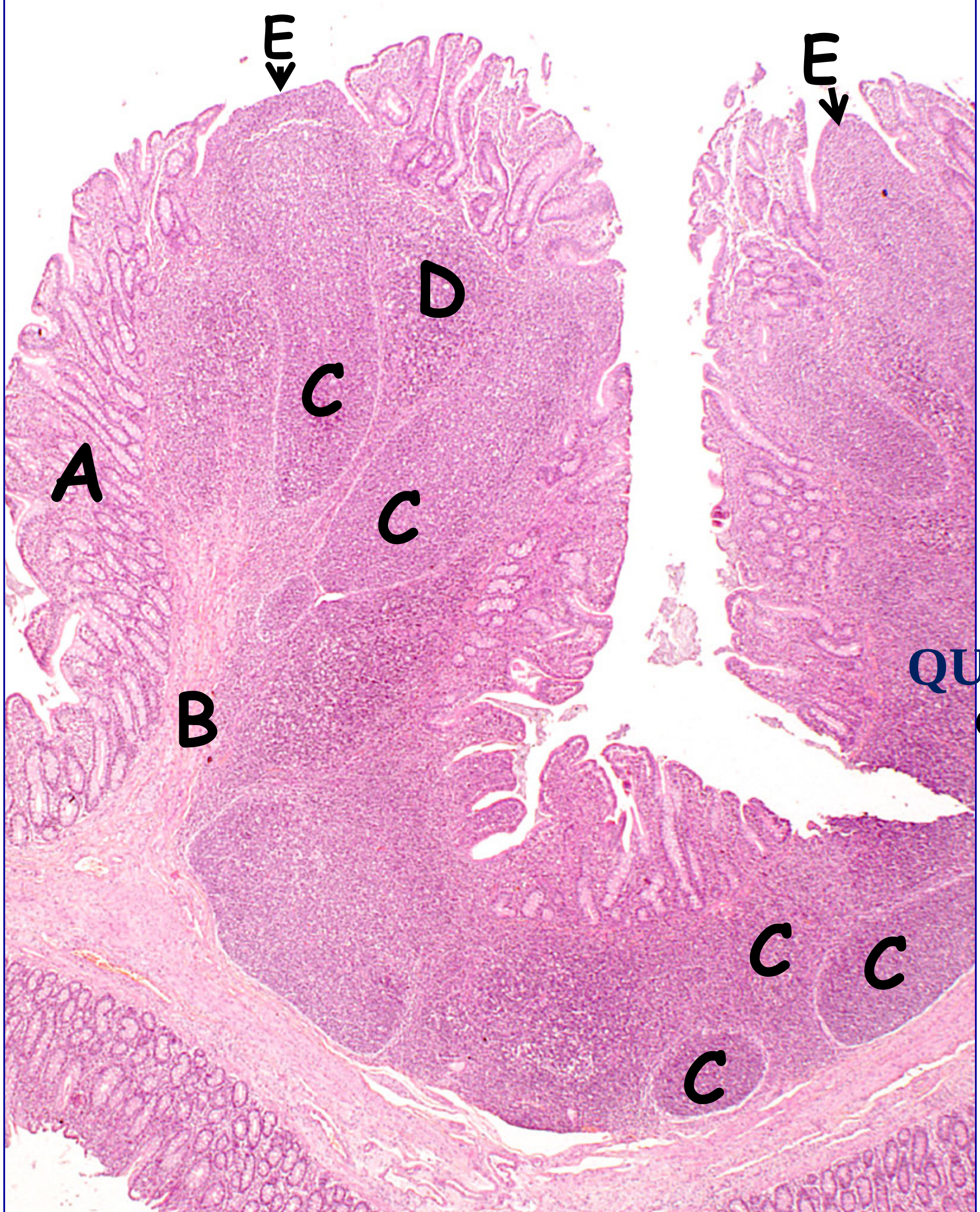

¿Qué ESTRUCTURA está en pared de intestino?

QUESTIONNAIRE II

Citology & Histology

ÓRGANO

QUESTIONNAIRE II

Citology & Histology

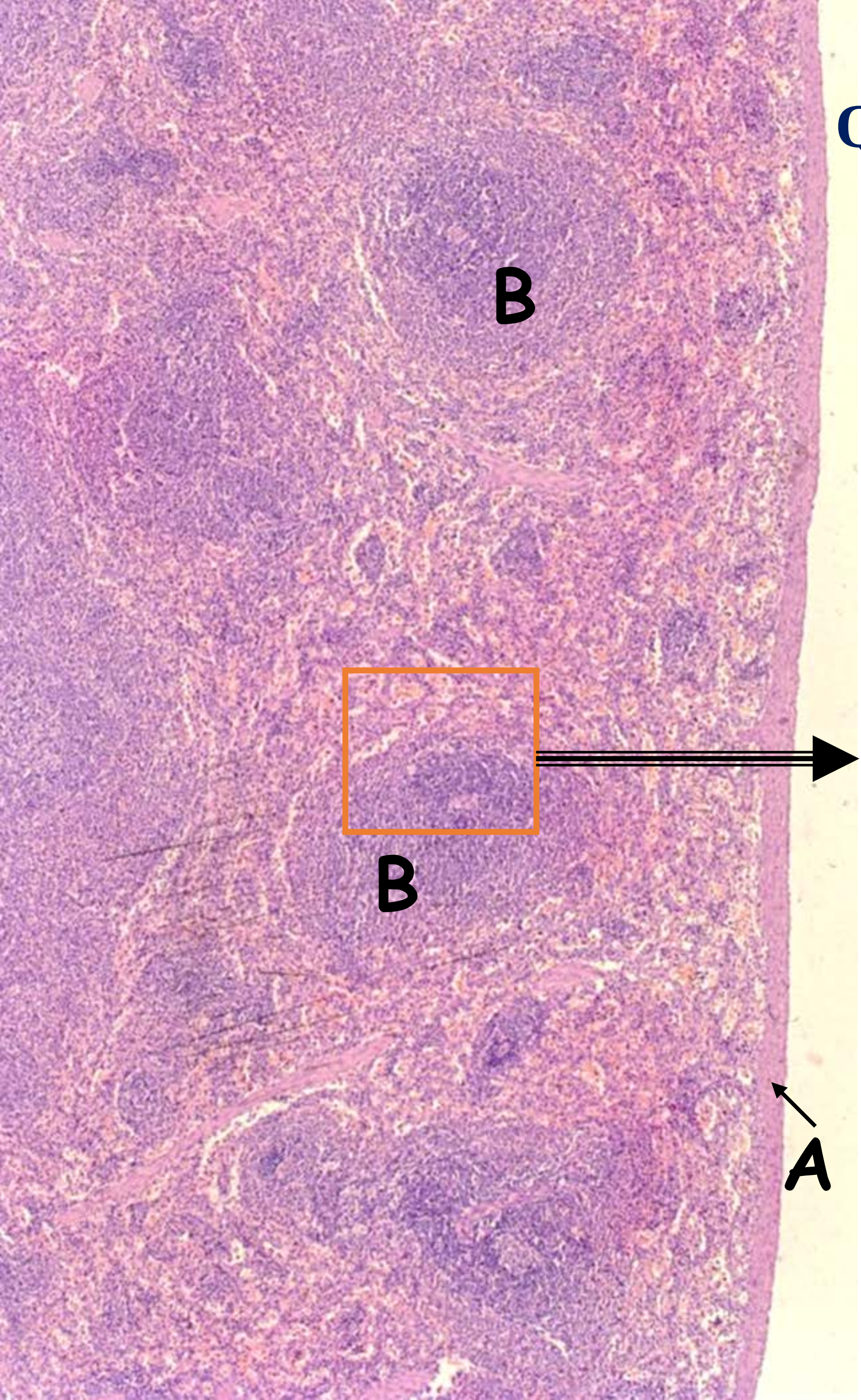

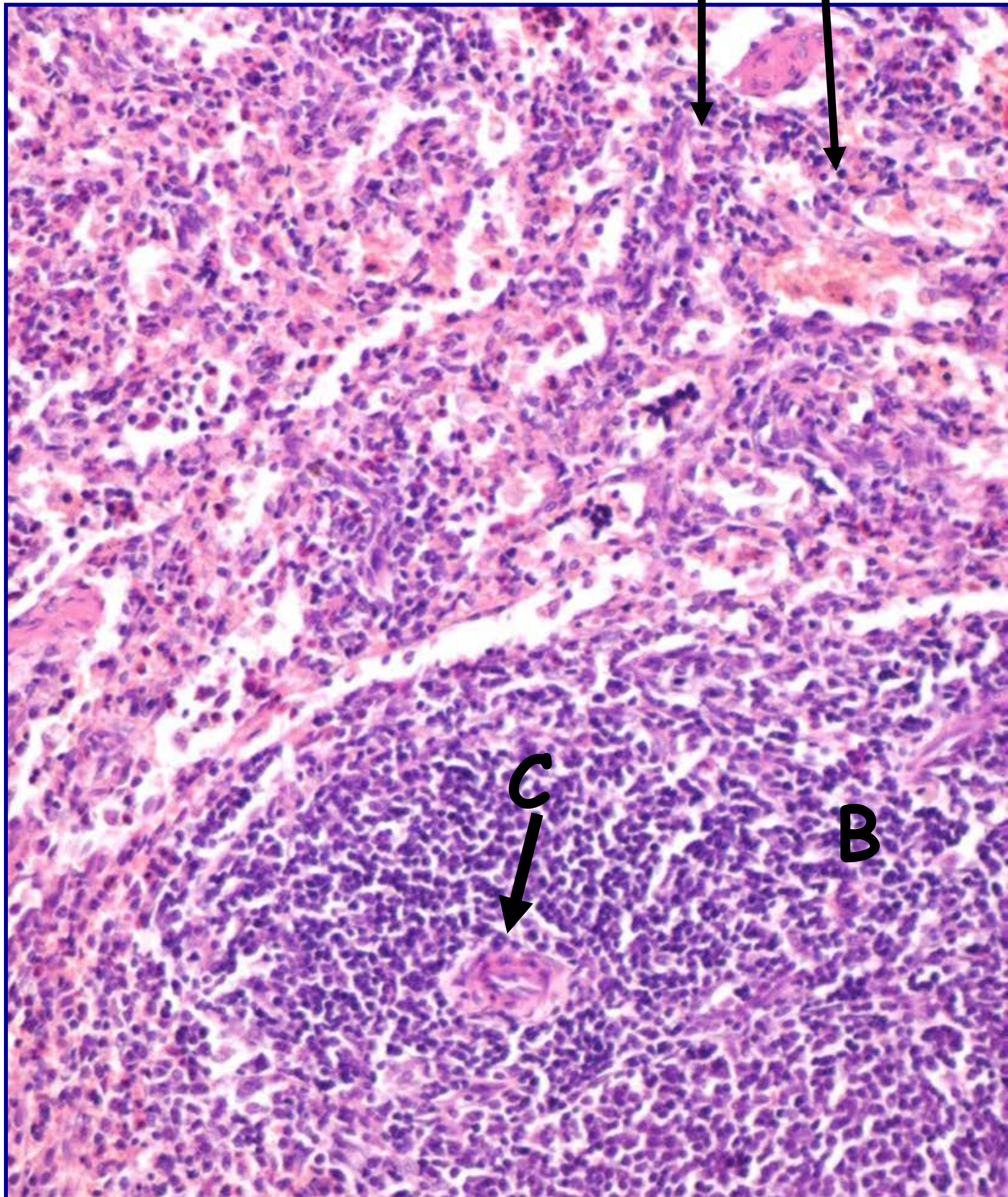

0106005: TUBAVET: material III.

https://agora.unileon.es/mod/quiz/view.php?id=30765

Español - España (es_es)

universidad de león

Área personal ▸ 0106005 ▸ General ▸ TUBAVET: material III.

NAVEGACIÓN

- Área personal
  - Inicio del sitio
  - Páginas del sitio
  - Mi perfil
  - Curso actual
    - 0106005
      - Participantes
      - Insignias
      - General
        - Novedades
        - Foro de Noticias
        - Convocatoria de práctica en laboratorio
        - TUBAVET-Material I
        - Cuestionario I: técnica histológica y citología.
        - **TUBAVET: material III.**
        - Encuesta de satisfacción TUBAVET

TUBAVET: material III.

TUBAVET: Material III

A continuación se presenta una batería de preguntas relacionadas con la segunda parte del temario visto en la asignatura de *Citología e Histología*.

Las preguntas son de diversos tipos (preguntas de emparejamiento o para rellenar huecos). No se penaliza si no se selecciona la respuesta correcta, y no hay límite de tiempo ni de intentos.

No te olvides, por favor, de enviarlo una vez finalizado. Muchas gracias.

Para contestar este cuestionario necesita conocer la contraseña

Método de calificación: Calificación más alta

No se permiten más intentos

Volver al curso

QUESTIONNAIRE III

Citology & Histology

TUBAVET: material III.

https://agora.unileon.es/mod/quiz/attempt.php?attempt=36354

universidad de león

Área personal ▸ 0106005 ▸ General ▸ TUBAVET: material III.

NAVEGACIÓN POR EL CUESTIONARIO

1 2 3 4 5 6 7 8

9 10 11 12 13 14 15 16

17 18 19 20

Terminar intento...

Pregunta 1

Sin responder aún

Puntúa como 1,00

Marcar pregunta

ÓRGANO:

TRAMO:

¿QUÉ ES A y B?

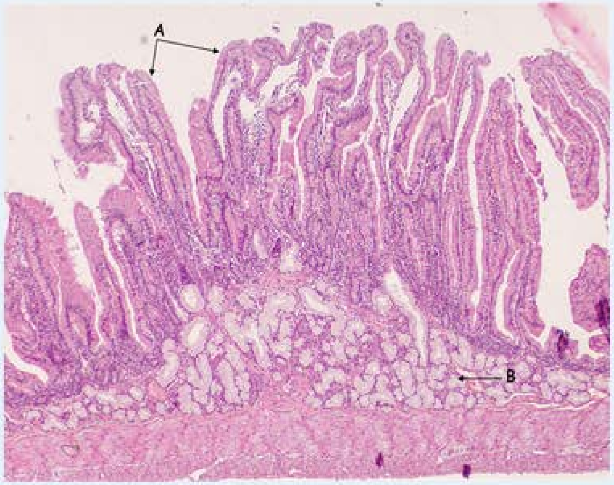

ÓRGANO Elegir...

TRAMO Elegir...

A Elegir...

B Elegir...

QUESTIONNAIRE III

Citology & Histology

NAVEGACIÓN POR EL CUESTIONARIO
1 2 3 4 5 6 7 8
9 10 11 12 13 14 15 16
17 18 19 20
Terminar intento...

QUESTIONNAIRE III

Citology & Histology

Pregunta 2

Sin responder aún

Puntúa como 1,00

Marcar pregunta

ÓRGANO:

¿QUÉ ES A, B, C?

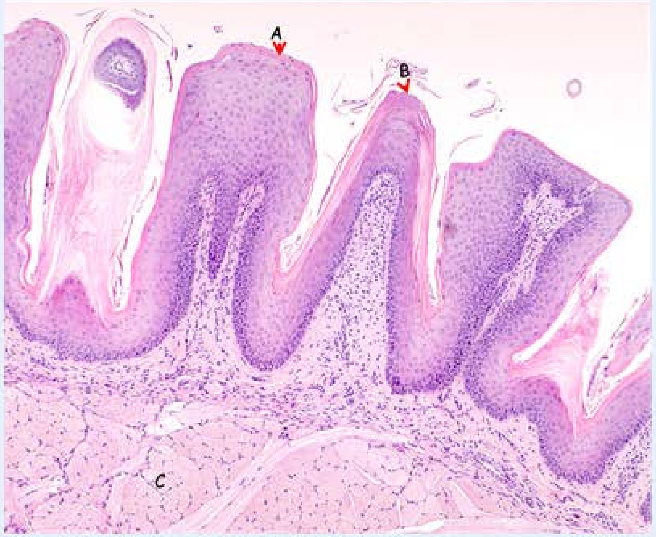

Órgano: Elegir...

A: Papila fungiforme

B: Elegir...

C: Elegir...

TUBAVET: material III.

https://agora.unileon.es/mod/quiz/attempt.php?attempt=36354&page=2

NAVEGACIÓN POR EL CUESTIONARIO

1 2 3 4 5 6 7 8
9 10 11 12 13 14 15 16
17 18 19 20

Terminar intento...

Pregunta 3

Sin responder aún

Puntúa como 1,00

Marcar pregunta

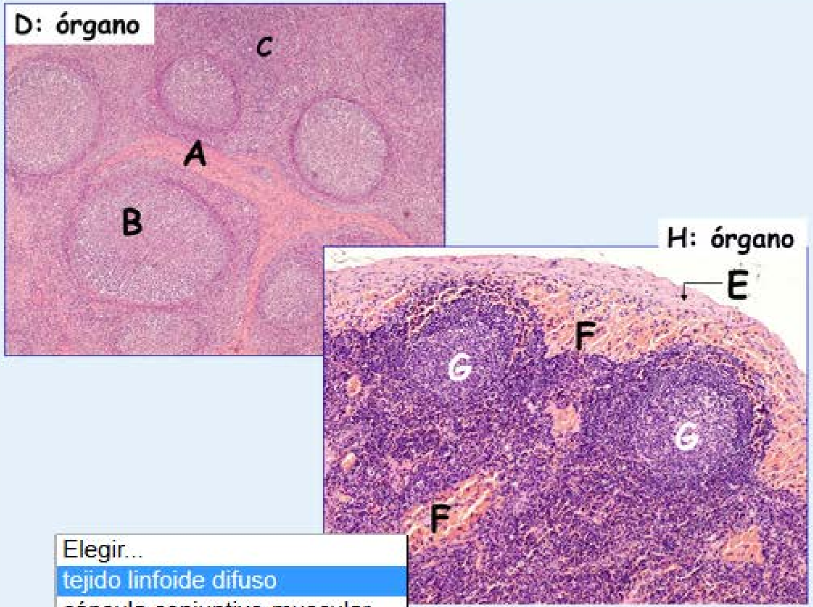

Elegir...
tejido linfoide difuso
cápsula conjuntivo-muscular
Nódulo hemolinfático
senos con sangre
NL porcino
senos con linfa
trabécula conjuntiva
folículo linfoide

A

B

C Elegir...

D Elegir...

E Elegir...

F Elegir...

QUESTIONNAIRE III

Citology & Histology

TUBAVET: material III.

https://agora.unileon.es/mod/quiz/attempt.php?attempt=36354&page=3

Área personal ▸ 0106005 ▸ General ▸ TUBAVET: material III.

NAVEGACIÓN POR EL CUESTIONARIO

1 2 3 4 5 6 7 8
9 10 11 12 13 14 15 16
17 18 19 20

Terminar intento...

Pregunta 4

Sin responder aún

Puntúa como 1,00

Marcar pregunta

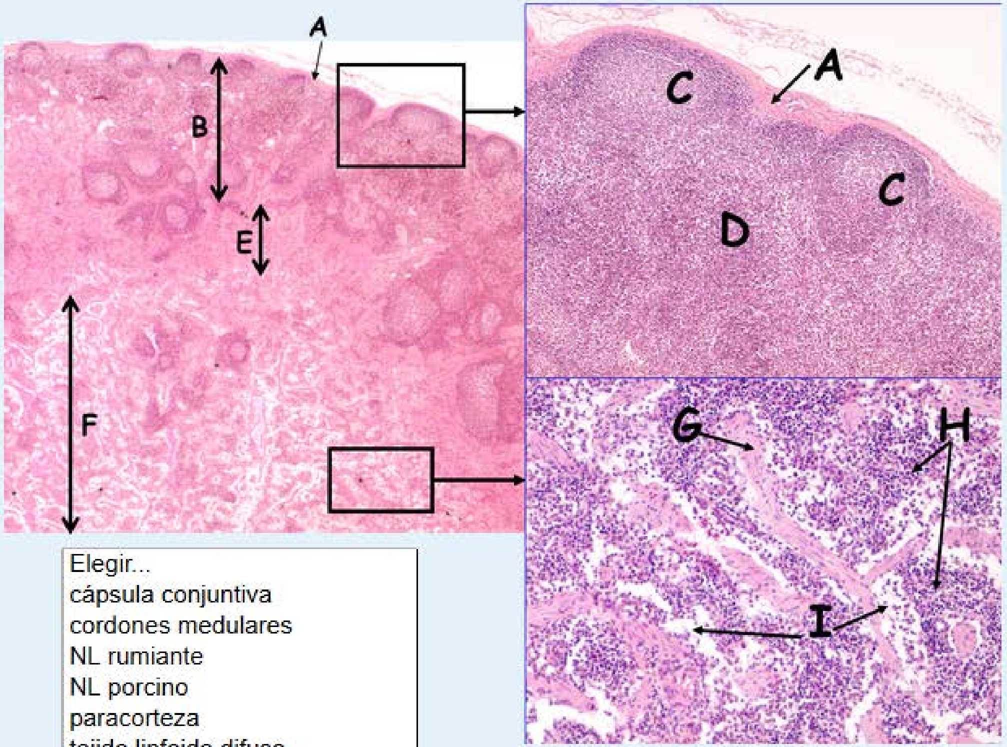

- Elegir...
- cápsula conjuntiva
- cordones medulares
- NL rumiante
- NL porcino
- paracorteza
- tejido linfoide difuso
- corteza
- folículos linfoides
- senos medulares
- trabéculas conjuntivas
- cápsula conjuntivo-muscular
- médula

A

B

C Elegir...

D Elegir...

QUESTIONNAIRE III

Citology & Histology

TUBAVET: material III.

https://agora.unileon.es/mod/quiz/attempt.php?attempt=36354&page=4

Área personal ▸ 0106005 ▸ General ▸ TUBAVET: material III.

NAVEGACIÓN POR EL CUESTIONARIO

1 2 3 4 5 6 7 8
9 10 11 12 13 14 15 16
17 18 19 20

Terminar intento...

Pregunta 5

Sin responder aún

Puntúa como 1,00

Marcar pregunta

ÓRGANO:
¿QUÉ ES A, B y C?

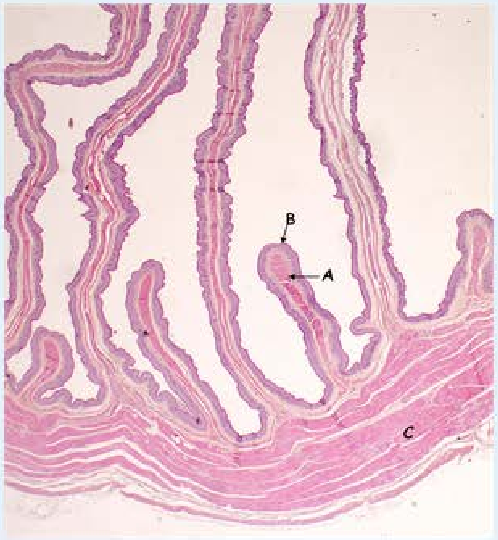

ÓRGANO Elegir...

A Elegir...

B Elegir...

C Elegir...

QUESTIONNAIRE III

Citology & Histology

Siguiente

TUBAVET: material III.

https://agora.unileon.es/mod/quiz/attempt.php?attempt=36354&page=5

Área personal ▸ 0106005 ▸ General ▸ TUBAVET: material III.

NAVEGACIÓN POR EL CUESTIONARIO

1 2 3 4 5 6 7 8
9 10 11 12 13 14 15 16
17 18 19 20

Terminar intento...

Pregunta 6

Sin responder aún

Puntúa como 1,00

Marcar pregunta

F: órgano

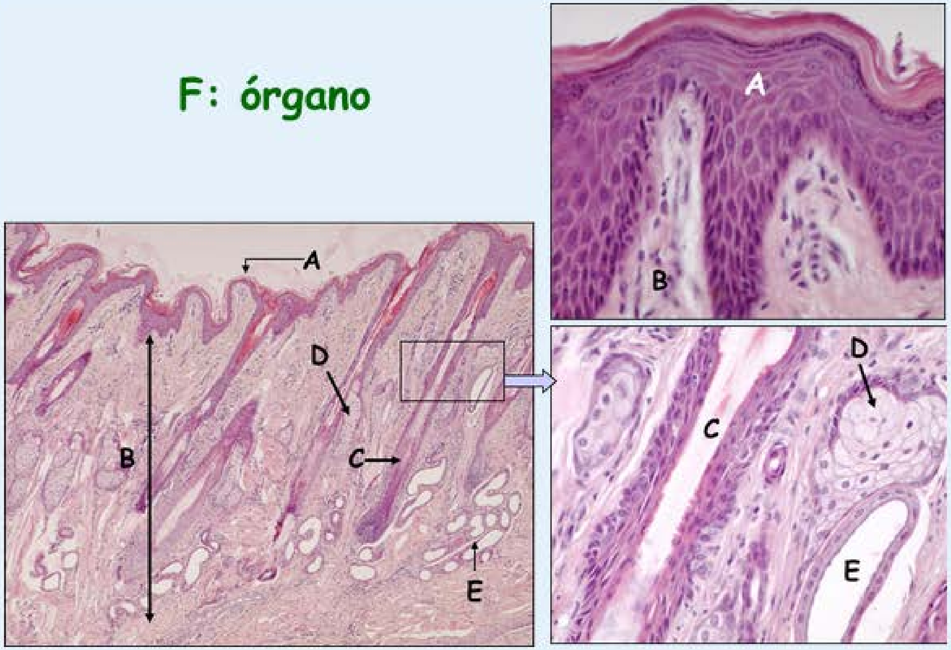

A Elegir...

B Elegir...

C Elegir...

D Elegir...

E Elegir...

QUESTIONNAIRE III

Citology & Histology

TUBAVET: material III.

https://agora.unileon.es/mod/quiz/attempt.php?attempt=36354&page=6

Área personal ▸ 0106005 ▸ General ▸ TUBAVET: material III.

NAVEGACIÓN POR EL CUESTIONARIO

1 2 3 4 5 6 7 8
9 10 11 12 13 14 15 16
17 18 19 20

Terminar intento...

Cuestionario

Pregunta 7

Sin responder aún

Puntúa como 1,00

Marcar pregunta

ÓRGANO:

¿QUÉ ES A y B?

¿COMO SE DENOMINA A LA ESTRUCTURA FORMADA POR A Y B?

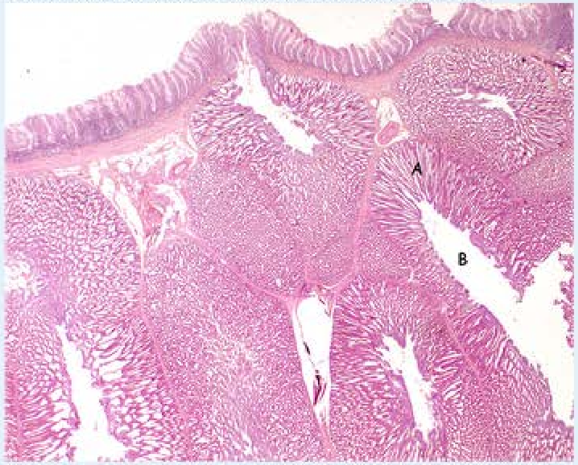

Órgano	Elegir...
A	Elegir...
B	Elegir...
¿Cómo se denomina la estructura formada por A y B?	Elegir...

QUESTIONNAIRE III

Citology & Histology

TUBAVET: material III.

https://agora.unileon.es/mod/quiz/attempt.php?attempt=36354&page=7

Área personal ▸ 0106005 ▸ General ▸ TUBAVET: material III.

NAVEGACIÓN POR EL CUESTIONARIO

1 2 3 4 5 6 7 8
9 10 11 12 13 14 15 16
17 18 19 20

Terminar intento...

Pregunta 8

Sin responder aún

Puntúa como 1,00

Marcar pregunta

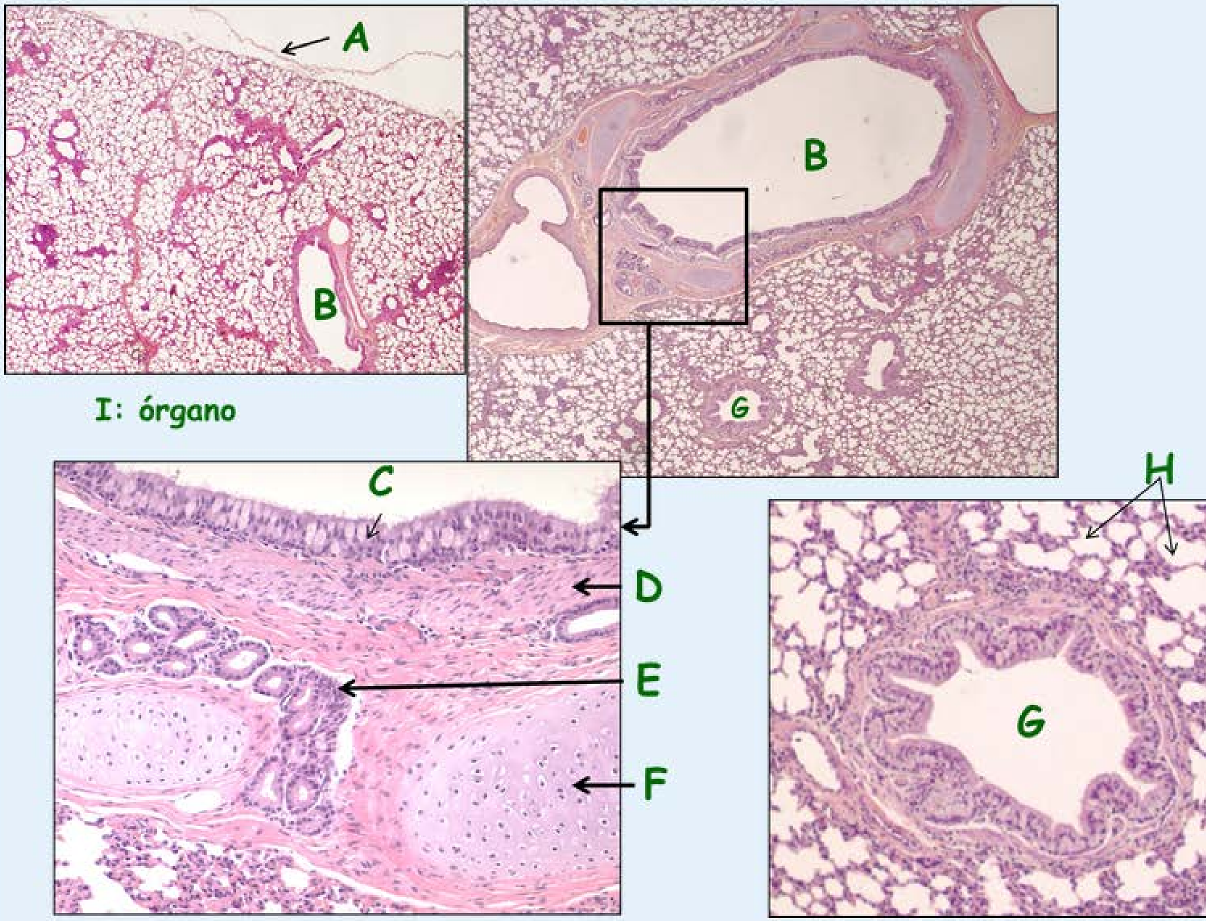

QUESTIONNAIRE III

Citology & Histology

A Elegir...

TUBAVET: material III.

https://agora.unileon.es/mod/quiz/attempt.php?attempt=36354&page=8

Área personal ▸ 0106005 ▸ General ▸ TUBAVET: material III.

NAVEGACIÓN POR EL CUESTIONARIO

1 2 3 4 5 6 7 8
9 10 11 12 13 14 15 16
17 18 19 20

Terminar intento...

Pregunta 9

Sin responder aún

Puntúa como 1,00

Marcar pregunta

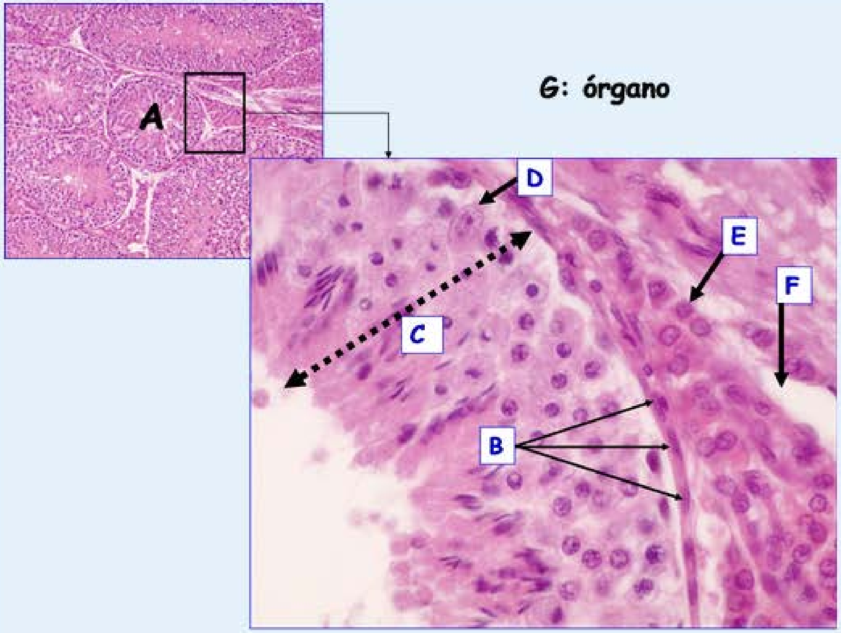

G: órgano

A Elegir...

B Elegir...

C Elegir...

D Elegir...

E Elegir...

QUESTIONNAIRE III

Citology & Histology

TUBAVET: material III.

https://agora.unileon.es/mod/quiz/attempt.php?attempt=36354&page=9

Área personal ▸ 0106005 ▸ General ▸ TUBAVET: material III.

NAVEGACIÓN POR EL CUESTIONARIO

1 2 3 4 5 6 7 8
9 10 11 12 13 14 15 16
17 18 19 20

Terminar intento...

Pregunta 10

Sin responder aún

Puntúa como 1,00

Marcar pregunta

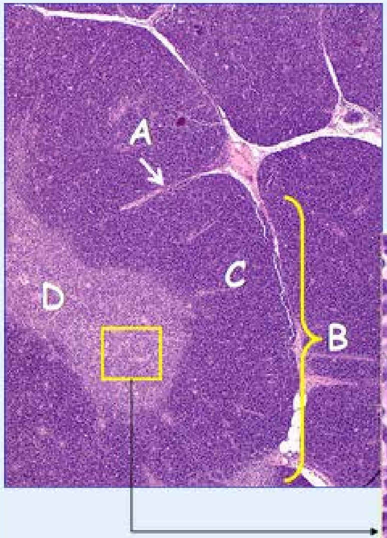

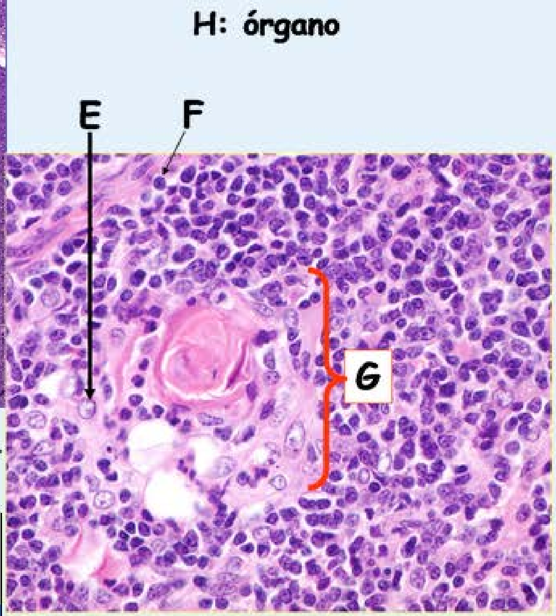

- Elegir...
- células retículoepiteliales
- corpúsculo tímico
- Corteza
- Médula
- Lobulillo incompleto
- linfocitos
- Timo
- Macrófagos
- tabique conjuntivo

A

B

C Elegir...

D Elegir...

QUESTIONNAIRE III

Citology & Histology

TUBAVET: material III.

https://agora.unileon.es/mod/quiz/attempt.php?attempt=36354&page=10

Google

Área personal ▸ 0106005 ▸ General ▸ TUBAVET: material III.

NAVEGACIÓN POR EL CUESTIONARIO

1 2 3 4 5 6 7 8

9 10 11 12 13 14 15 16

17 18 19 20

Terminar intento...

Pregunta 11

Sin responder aún

Puntúa como 1,00

Marcar pregunta

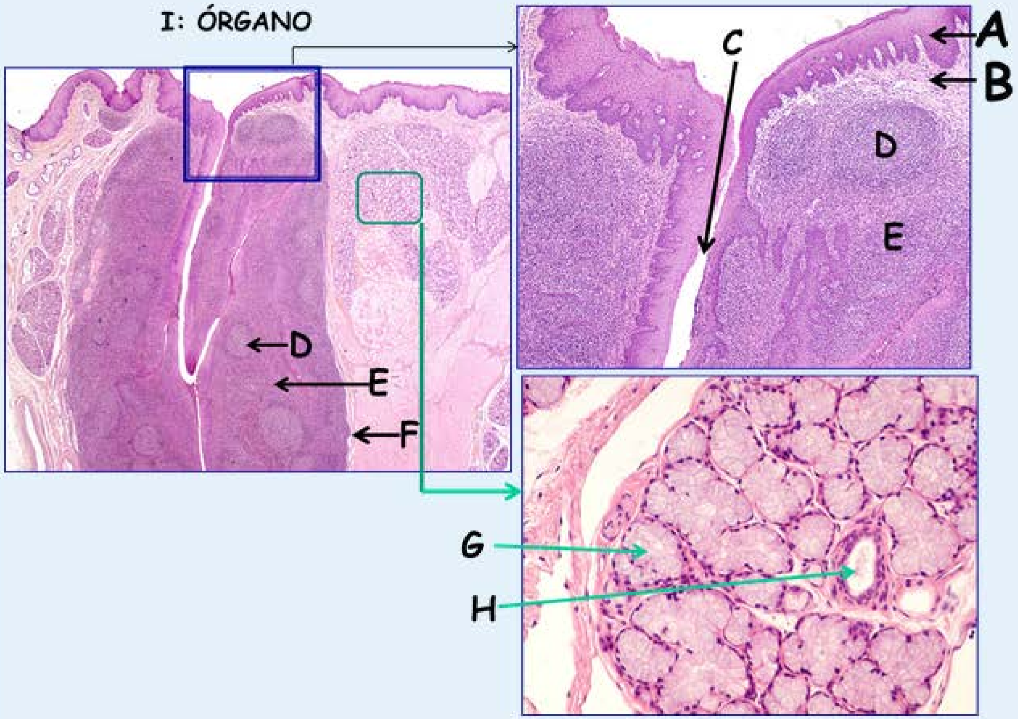

QUESTIONNAIRE III

Citology & Histology

A Elegir...

B Elegir...

C Elegir...

D Elegir...

TUBAVET: material III.

https://agora.unileon.es/mod/quiz/attempt.php?attempt=36354&page=11

Google

Área personal 0106005 General TUBAVET: material III.

NAVEGACIÓN POR EL CUESTIONARIO

1 2 3 4 5 6 7 8
9 10 11 12 13 14 15 16
17 18 19 20

Terminar intento...

Pregunta 12

Sin responder aún

Puntúa como 1,00

Marcar pregunta

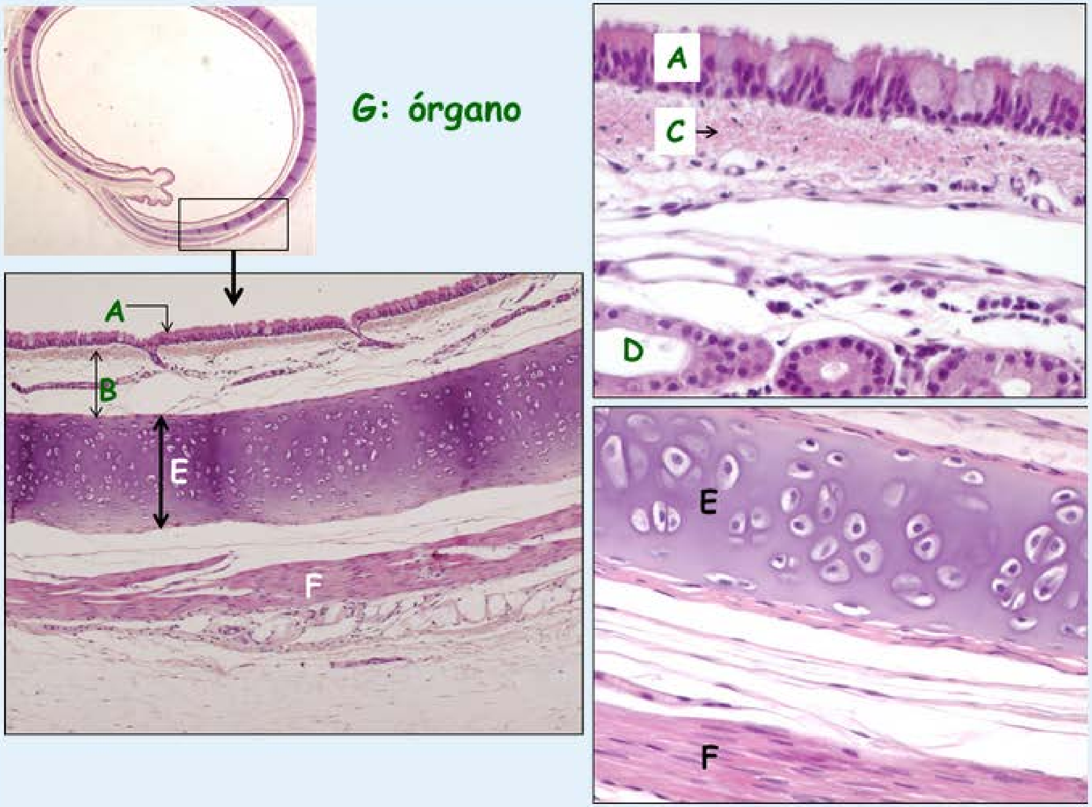

A Elegir...

B Elegir...

C Elegir...

QUESTIONNAIRE III

Citology & Histology

TUBAVET: material III.

https://agora.unileon.es/mod/quiz/attempt.php?attempt=36354&page=12

Área personal ▸ 0106005 ▸ General ▸ TUBAVET: material III.

NAVEGACIÓN POR EL CUESTIONARIO

1 2 3 4 5 6 7 8
9 10 11 12 13 14 15 16
17 18 19 20

Terminar intento...

Pregunta **13**

Sin responder aún

Puntúa como 1,00

Marcar pregunta

ÓRGANO:

¿QUÉ ES A, B y C?

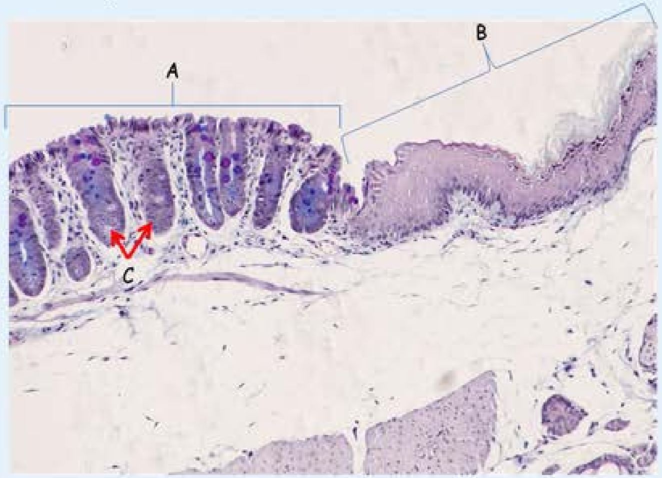

ÓRGANO

A

B

C

Elegir...
Glándulas gástricas
Estómago aglandular
Recto
Región cardial
Glándulas intestinales
Ano
Intestino grueso

Elegir...

QUESTIONNAIRE III

Citology & Histology

Siguiente

TUBAVET: material III.

https://agora.unileon.es/mod/quiz/attempt.php?attempt=36354&page=13

Área personal ▸ 0106005 ▸ General ▸ TUBAVET: material III.

NAVEGACIÓN POR EL CUESTIONARIO

1 2 3 4 5 6 7 8
9 10 11 12 13 14 15 16
17 18 19 20

Terminar intento...

Pregunta **14**

Sin responder aún

Puntúa como 1,00

Marcar pregunta

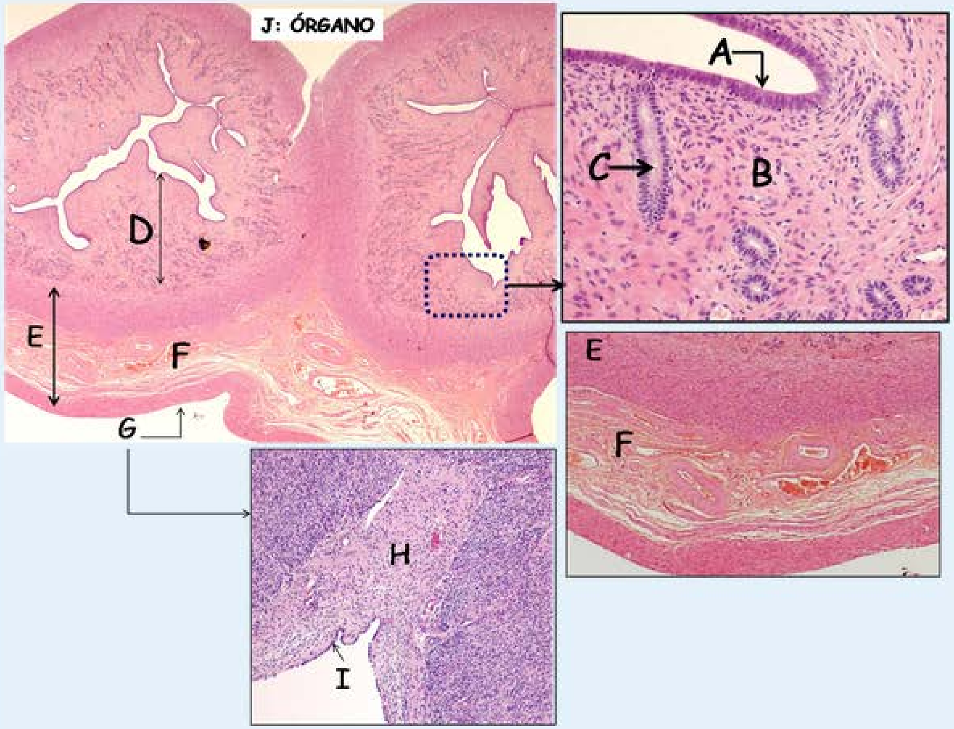

A Elegir...

B Elegir...

C Elegir...

D Elegir...

QUESTIONNAIRE III

Citology & Histology

TUBAVET: material III.

https://agora.unileon.es/mod/quiz/attempt.php?attempt=36354&page=14

Área personal ▸ 0106005 ▸ General ▸ TUBAVET: material III.

NAVEGACIÓN POR EL CUESTIONARIO

1 2 3 4 5 6 7 8

9 10 11 12 13 14 15 16

17 18 19 20

Terminar intento...

Pregunta 15

Sin responder aún

Puntúa como 1,00

Marcar pregunta

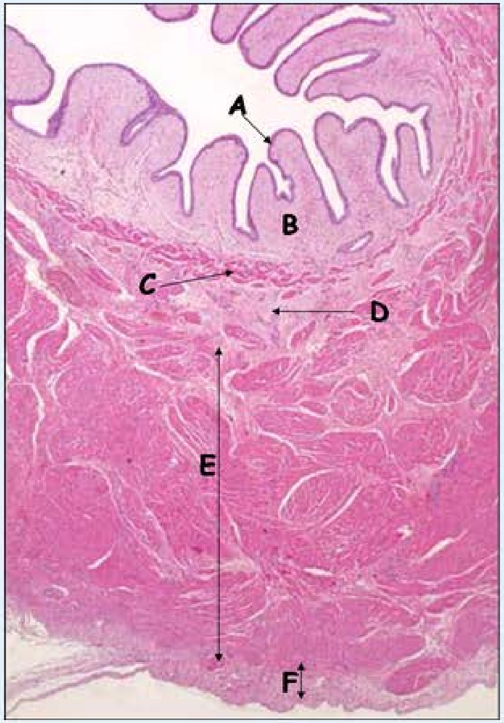

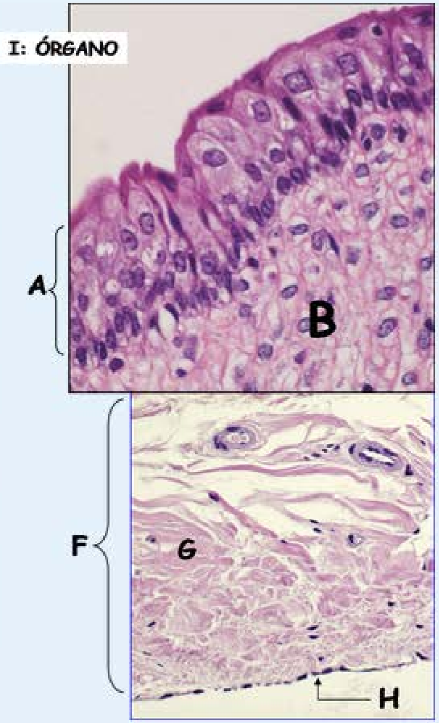

A Elegir...

B Elegir...

C Elegir...

D Elegir...

QUESTIONNAIRE III

Citology & Histology

TUBAVET: material III.

https://agora.unileon.es/mod/quiz/attempt.php?attempt=36354&page=15

Área personal ▸ 0106005 ▸ General ▸ TUBAVET: material III.

NAVEGACIÓN POR EL CUESTIONARIO

1 2 3 4 5 6 7 8
9 10 11 12 13 14 15 16
17 18 19 20

Terminar intento...

Pregunta **16**

Sin responder aún

Puntúa como 1,00

Marcar pregunta

El órgano está rodeado por (A) ______ . En el interior se observa dos partes. Una constituida por (B) ______ y (C) ______ y forma la ______ . La otra está constituida por ______ que rodea a ______ y constituye la (E) ______ . El órgano es ______ .

F: órgano

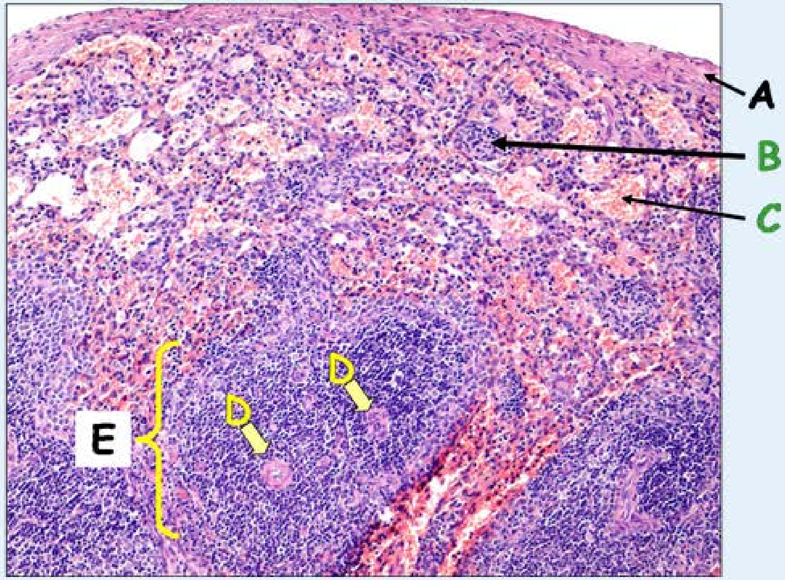

Siguiente

QUESTIONNAIRE III

Citology & Histology

TUBAVET: material III.

https://agora.unileon.es/mod/quiz/attempt.php?attempt=36354&page=16

Área personal ▸ 0106005 ▸ General ▸ TUBAVET: material III.

NAVEGACIÓN POR EL CUESTIONARIO

1 2 3 4 5 6 7 8
9 10 11 12 13 14 15 16
17 18 19 20

Terminar intento...

Pregunta 17

Sin responder aún

Puntúa como 1,00

Marcar pregunta

El órgano está rodeado por una (A) ____ en la que hay (B) ____. En el interior se encuentra tejido (C) ____ con (g) ____ en cuyo seno se encuentran diferentes secciones tubulares del (D) ____. Este D está revestido por un (E) ____ y está rodeado de (F) ____. El órgano es (H) ____ y la tinción es ____.

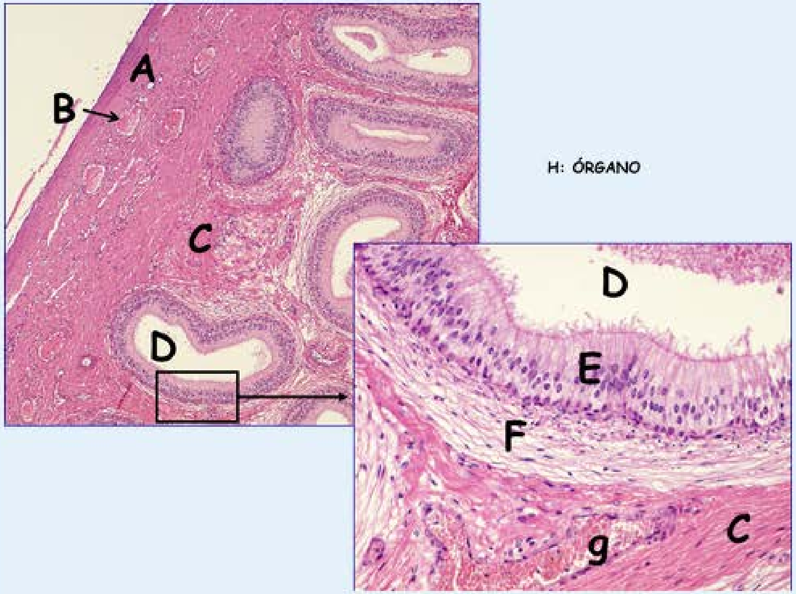

QUESTIONNAIRE III

Citology & Histology

Siguiente

TUBAVET: material III.
https://agora.unileon.es/mod/quiz/attempt.php?attempt=36354&page=17
Google

Pregunta 18

Sin responder aún

Puntúa como 1,00

Marcar pregunta

El órgano está rodeado de (A) ________ . En el interior está dividido en unidades anatómicas (B) ________ que están formados por (C) ________ , (D) ________ dispuestos en ________ y, entre ellas, (E) ________ . En la confluencia de diferentes (B), se encuentran ________ que están constituidos por (G) ________ , (F) ________ y (H) ________ . El órgano es (I) ________ .

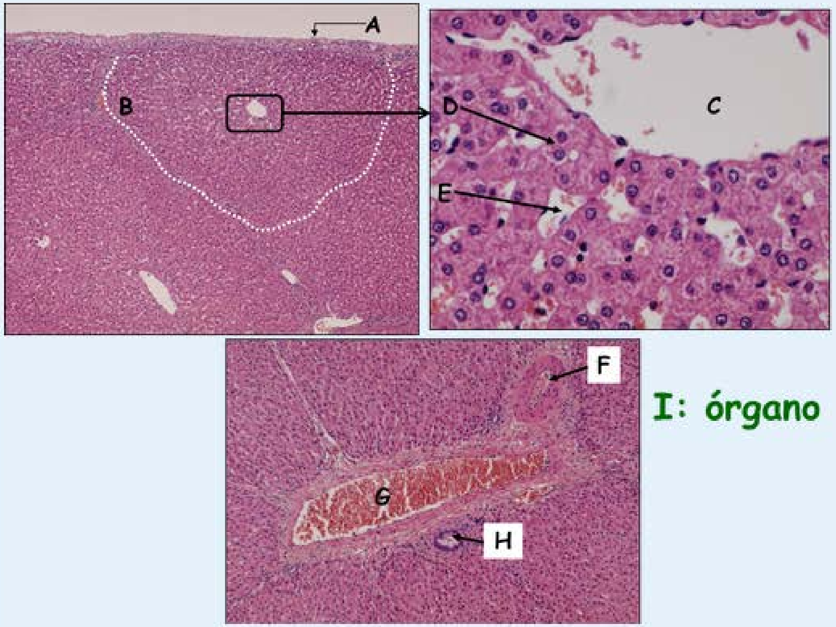

QUESTIONNAIRE III
Citology & Histology

Siguiente

TUBAVET: material III.

https://agora.unileon.es/mod/quiz/attempt.php?attempt=36354&page=18

Área personal ▸ 0106005 ▸ General ▸ TUBAVET: material III.

NAVEGACIÓN POR EL CUESTIONARIO

1 2 3 4 5 6 7 8
9 10 11 12 13 14 15 16
17 18 19 20

Terminar intento...

Pregunta 19

Sin responder aún

Puntúa como 1,00

Marcar pregunta

El órgano está rodeado externamente por (A) [▾] y debajo se encuentra (B) [▾] de tejido [▾] . Se distinguen dos zonas: una externa denominada (C) [▾] y otra central denominada (D) [▾] . En la externa, se encuentran los [▾] entre los que se puede citar (D) [▾] y (E) [▾] . Igualmente, en la (C) se localizan (F) [▾] que es una [▾] formada por (G) [▾] y (H) [▾] . La D se caracteriza por tener muchos [▾] . El órgano es (I) [▾] y su tinción es [▾] .

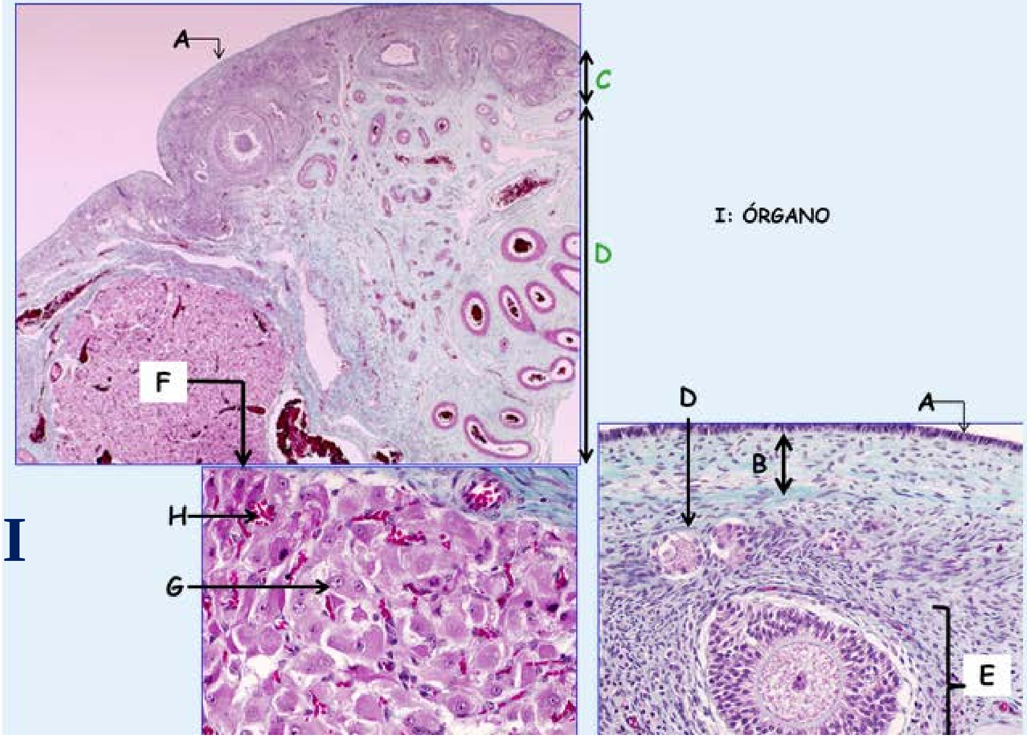

QUESTIONNAIRE III

Citology & Histology

Pregunta **20**

Sin responder aún

Puntúa como 1,00

Marcar pregunta

El órgano está rodeado externamente por (A) ______ . En el mismo se observan dos regiones: una externa (B) denominada ______ y otra interna (C), denominada ______ . En la externa se localizan (D) ______ que están formados por (E) ______ que están rodeados por (F) ______ . Igualmente, en esta (B), se encuentran los (H) ______ y los (G) ______ que en las proximidades de (D) el epitelio se hace más alto y forma (I) ______ . En la zona más interna (C), se encuentran (J) ______ . El órgano es ______ y la tinción es ______ .

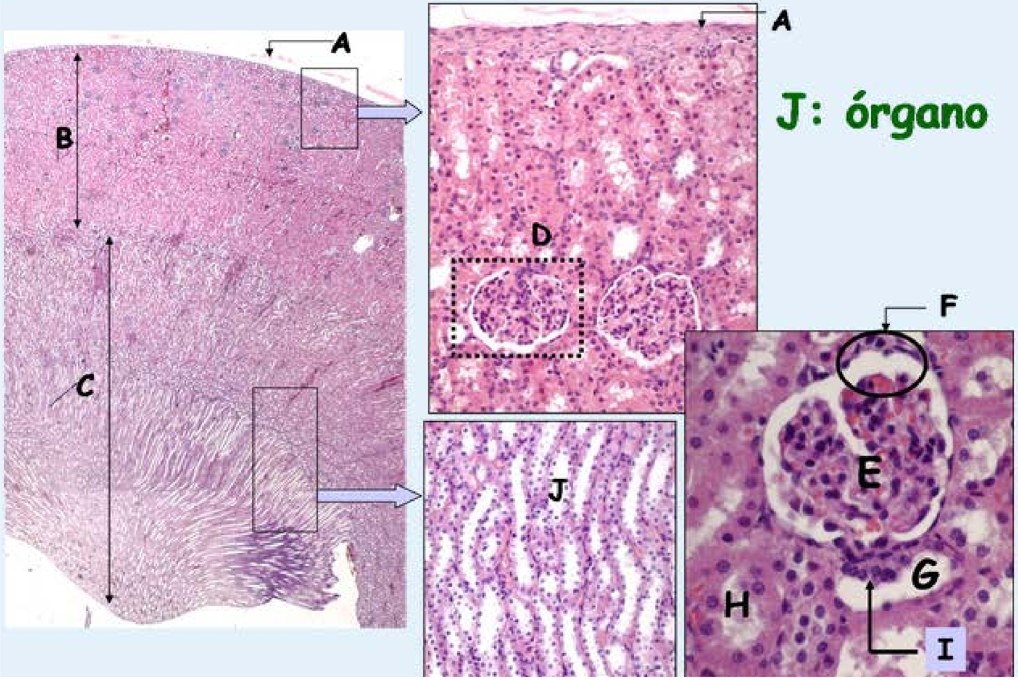

QUESTIONNAIRE III

Citology & Histology

QUESTIONNAIRE I

Veterinary Pharmacology

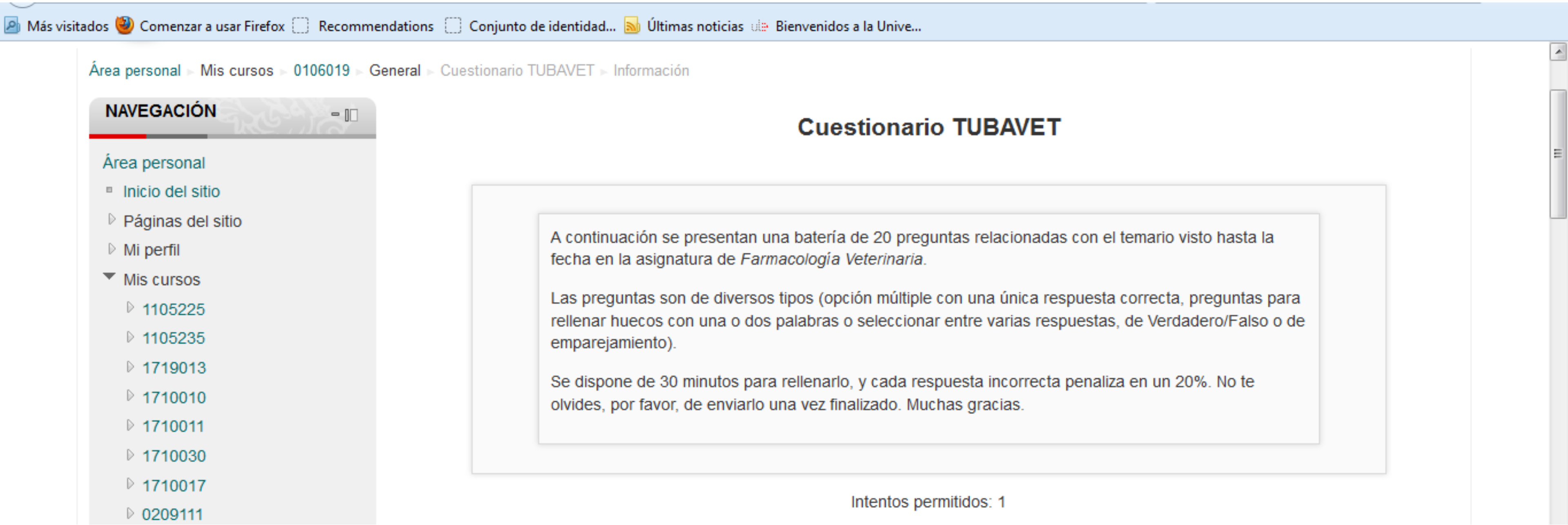

QUESTIONNAIRE I

Veterinary Pharmacology

Más visitados · Comenzar a usar Firefox · Recommendations · Conjunto de identidad... · Últimas noticias · Bienvenidos a la Unive...

Área personal ▸ Mis cursos ▸ 0106019 ▸ General ▸ Cuestionario TUBAVET ▸ Vista previa

NAVEGACIÓN POR EL CUESTIONARIO

1 2 3 4 5 6 7 8
9 10 11 12 13 14 15 16
17 18 19 20

Terminar intento...

Tiempo restante 0:26:27

Comenzar una nueva previsualización

NAVEGACIÓN

Área personal
- Inicio del sitio
- Páginas del sitio
- Mi perfil
- Mis cursos
  - 1105225
  - 1105235
  - 1719013
  - 1710010
  - 1710011
  - 1710030

Pregunta 1
Sin responder aún
Puntúa como 1,00
Marcar pregunta
Editar pregunta

La materia prima de origen natural (animal, vegetal o mineral), así como los principios activos que pueden extraerse de las mismas y que tienen una determinada actividad biológica, se denomina ______

Pregunta 2
Sin responder aún
Puntúa como 1,00
Marcar pregunta
Editar pregunta

Señale la respuesta correcta con relación a la unión a las proteínas plasmáticas:

Seleccione una:
- a. El fármaco unido a las proteínas plasmáticas ni se distribuye ni se elimina
- b. se pueden producir fenómenos de competición entre los fármacos por unirse a las proteínas plasmáticas
- c. el grado de unión puede variar con la especie o la presencia de una insuficiencia hepática
- d. todas son correctas

Pregunta 3
Sin responder aún
Puntúa como 4,00
Marcar pregunta
Editar pregunta

Relacione el tipo de compuesto empleado en la neuroleptoanestesia con la acción desarrollada por éste:

antagonista muscarínico	Elegir...
neuroléptico	Elegir...
relajante muscular	Elegir...
opioide	Elegir...

QUESTIONNAIRE I

Veterinary Pharmacology

Más visitados | Comenzar a usar Firefox | Recommendations | Conjunto de identidad... | Últimas noticias | Bienvenidos a la Unive...

NAVEGACIÓN POR EL CUESTIONARIO

1 2 3 4 5 6 7 8
9 10 11 12 13 14 15 16
17 18 19 20

Terminar intento...

Tiempo restante **0:29:13**

Comenzar una nueva previsualización

NAVEGACIÓN

Área personal
- Inicio del sitio
- Páginas del sitio
- Mi perfil
- Mis cursos
  - 1105225
  - 1105235
  - 1719013
  - 1710010
  - 1710011
  - 1710030
  - 1710017
  - 0209111
  - [illegible]

Pregunta 4
Sin responder aún
Puntúa como 1,00
Marcar pregunta
Editar pregunta

En el caso de la inducción enzimática, relacione el tipo de metabolito formado con la correspondiente consecuencia de esta inducción enzimática:

metabolito activo — Elegir...
metabolito inactivo — Elegir...
metabolito tóxico — Elegir...

Pregunta 5
Sin responder aún
Puntúa como 1,00
Marcar pregunta
Editar pregunta

Los efectos colaterales:

Seleccione una:
- a. son un tipo de sobredosificación
- b. no se consideran reacciones adversas
- c. surgen como consecuencia de la acción farmacológica principal del compuesto
- d. forman parte de la propia acción farmacológica del compuesto, pero su aparición no es deseable

Pregunta 6
Sin responder aún
Puntúa como 1,00
Marcar pregunta
Editar pregunta

En el caso de la potenciación:

Seleccione una:
- a. el efecto que se produce es inferior a la suma de los efectos que se producen por separado
- b. los fármacos se unen al mismo tipo de receptor
- c. el efecto resultante de la administración conjunta de dos fármacos es superior a la suma de los efectos individuales
- d. los fármacos no deben tener la misma acción cualitativa

QUESTIONNAIRE I

Veterinary Pharmacology

Más visitados | Comenzar a usar Firefox | Recommendations | Conjunto de identidad... | Últimas noticias | Bienvenidos a la Unive...

Área personal ▸ Mis cursos ▸ 0106019 ▸ General ▸ Cuestionario TUBAVET ▸ Vista previa

NAVEGACIÓN POR EL CUESTIONARIO

1 2 3 4 5 6 7 8
9 10 11 12 13 14 15 16
17 18 19 20

Terminar intento...

Tiempo restante **0:28:51**

Comenzar una nueva previsualización

NAVEGACIÓN

Área personal
- Inicio del sitio
- Páginas del sitio
- Mi perfil
- Mis cursos
  - 1105225
  - 1105235
  - 1719013
  - 1710010
  - 1710011
  - 1710030

Pregunta 7
Sin responder aún
Puntúa como 1,00
Marcar pregunta
Editar pregunta

La resistencia exagerada a responder a la dosis usual de un fármaco se denomina:

Seleccione una:
- a. tolerancia
- b. alergia
- c. idiosincrasia
- d. intolerancia

Pregunta 8
Sin responder aún
Puntúa como 1,00
Marcar pregunta
Editar pregunta

Indique el nombre de la rama de la Farmacología que se ocupa del estudio de los procesos de absorción, distribución, metabolización y excreción de los fármacos.

Respuesta:

Pregunta 9
Sin responder aún
Puntúa como 1,00
Marcar pregunta
Editar pregunta

En el caso de la excreción renal, los fármacos de carácter ácido débil se reabsorberán en una orina:

Seleccione una:
- a. neutra
- b. no se reabsorben nunca
- c. ácida
- d. básica

QUESTIONNAIRE I

Veterinary Pharmacology

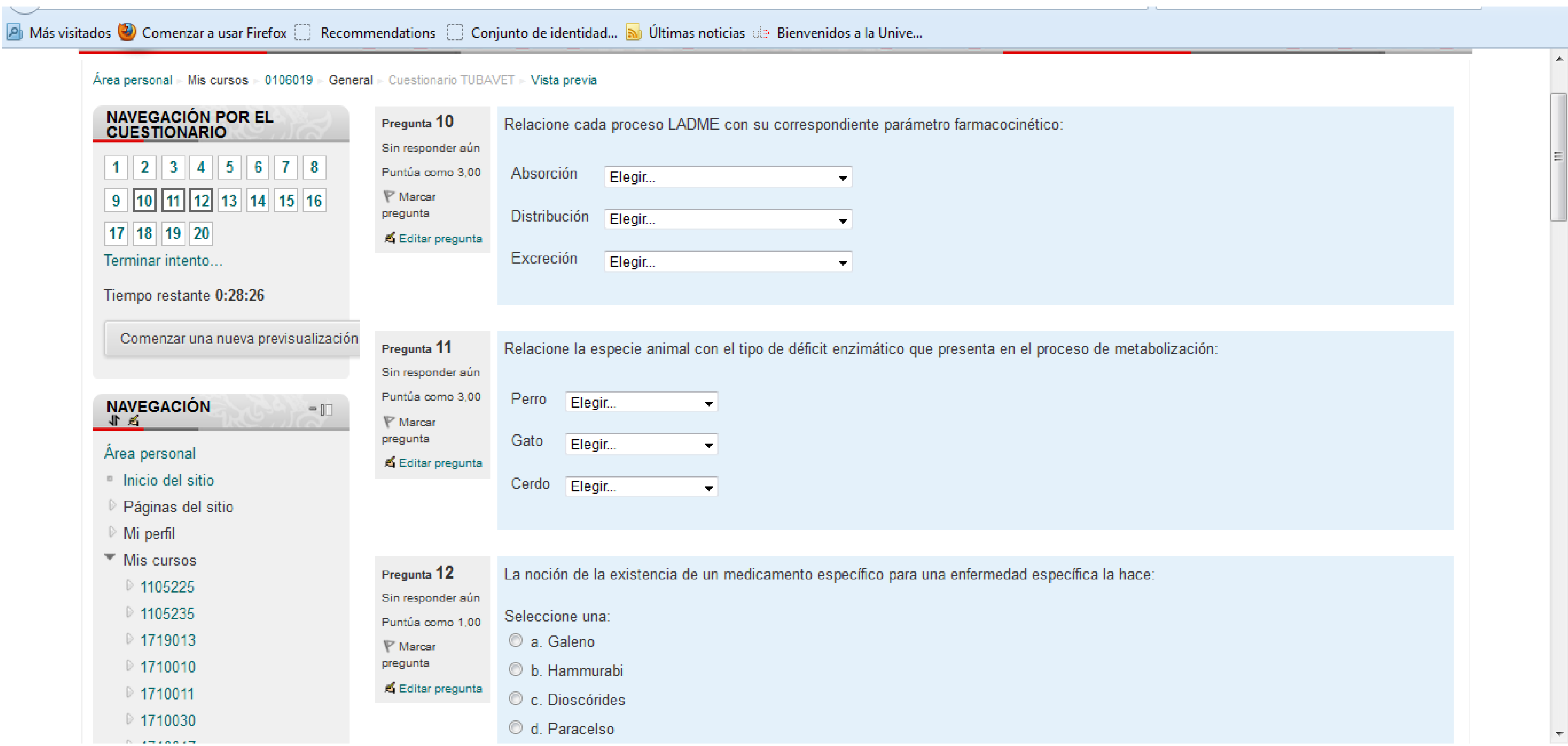

Más visitados | Comenzar a usar Firefox | Recommendations | Conjunto de identidad... | Últimas noticias | Bienvenidos a la Unive...

Área personal ▸ Mis cursos ▸ 0106019 ▸ General ▸ Cuestionario TUBAVET ▸ Vista previa

NAVEGACIÓN POR EL CUESTIONARIO

1 2 3 4 5 6 7 8
9 10 11 12 13 14 15 16
17 18 19 20

Terminar intento...

Tiempo restante **0:28:26**

Comenzar una nueva previsualización

NAVEGACIÓN

Área personal
- Inicio del sitio
- Páginas del sitio
- Mi perfil
- Mis cursos
  - 1105225
  - 1105235
  - 1719013
  - 1710010
  - 1710011
  - 1710030

Pregunta 10
Sin responder aún
Puntúa como 3,00
Marcar pregunta
Editar pregunta

Relacione cada proceso LADME con su correspondiente parámetro farmacocinético:

Absorción: Elegir...
Distribución: Elegir...
Excreción: Elegir...

Pregunta 11
Sin responder aún
Puntúa como 3,00
Marcar pregunta
Editar pregunta

Relacione la especie animal con el tipo de déficit enzimático que presenta en el proceso de metabolización:

Perro: Elegir...
Gato: Elegir...
Cerdo: Elegir...

Pregunta 12
Sin responder aún
Puntúa como 1,00
Marcar pregunta
Editar pregunta

La noción de la existencia de un medicamento específico para una enfermedad específica la hace:

Seleccione una:
- a. Galeno
- b. Hammurabi
- c. Dioscórides
- d. Paracelso

QUESTIONNAIRE I

Veterinary Pharmacology

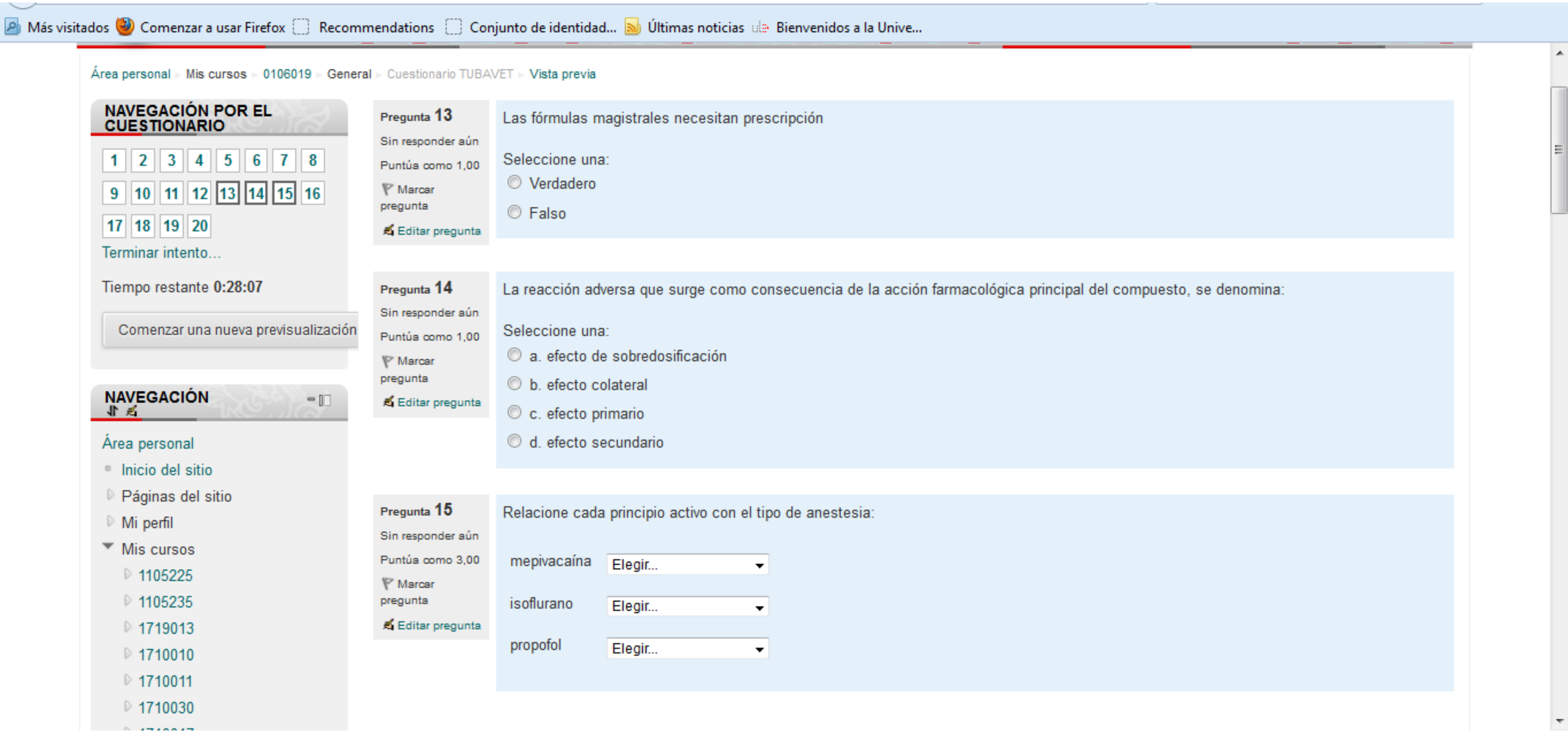

Más visitados | Comenzar a usar Firefox | Recommendations | Conjunto de identidad... | Últimas noticias | Bienvenidos a la Unive...

Área personal ▸ Mis cursos ▸ 0106019 ▸ General ▸ Cuestionario TUBAVET ▸ Vista previa

NAVEGACIÓN POR EL CUESTIONARIO

1 2 3 4 5 6 7 8
9 10 11 12 13 14 15 16
17 18 19 20

Terminar intento...

Tiempo restante **0:28:07**

Comenzar una nueva previsualización

NAVEGACIÓN

Área personal
- Inicio del sitio
- Páginas del sitio
- Mi perfil
- Mis cursos
  - 1105225
  - 1105235
  - 1719013
  - 1710010
  - 1710011
  - 1710030

Pregunta 13
Sin responder aún
Puntúa como 1,00
Marcar pregunta
Editar pregunta

Las fórmulas magistrales necesitan prescripción

Seleccione una:
- Verdadero
- Falso

Pregunta 14
Sin responder aún
Puntúa como 1,00
Marcar pregunta
Editar pregunta

La reacción adversa que surge como consecuencia de la acción farmacológica principal del compuesto, se denomina:

Seleccione una:
- a. efecto de sobredosificación
- b. efecto colateral
- c. efecto primario
- d. efecto secundario

Pregunta 15
Sin responder aún
Puntúa como 3,00
Marcar pregunta
Editar pregunta

Relacione cada principio activo con el tipo de anestesia:

mepivacaína	Elegir...
isoflurano	Elegir...
propofol	Elegir...

QUESTIONNAIRE I

Veterinary Pharmacology

Más visitados | Comenzar a usar Firefox | Recommendations | Conjunto de identidad... | Últimas noticias | Bienvenidos a la Unive...

NAVEGACIÓN POR EL CUESTIONARIO

1 2 3 4 5 6 7 8
9 10 11 12 13 14 15 16
17 18 19 20

Terminar intento...

Tiempo restante **0:27:45**

Comenzar una nueva previsualización

NAVEGACIÓN

Área personal
- Inicio del sitio
- Páginas del sitio
- Mi perfil
- Mis cursos
  - 1105225
  - 1105235
  - 1719013
  - 1710010
  - 1710011
  - 1710030
  - 1710017
  - 0209111

Pregunta 16
Sin responder aún
Puntúa como 1,00
Marcar pregunta
Editar pregunta

Los anestésicos inhalatorios:

Seleccione una:
- a. no deben ser volátiles a temperatura ambiente
- b. deberían producir una buena relajación muscular
- c. la b) y no deberían provocar depresión cardiorrespiratoria
- d. pueden ser irritantes para las vías respiratorias

Pregunta 17
Sin responder aún
Puntúa como 1,00
Marcar pregunta
Editar pregunta

Cuando dos fármacos no se pueden administrar conjuntamente al resultar incompatibles por sus propiedades físicas o químicas, se habla de:

Seleccione una:
- a. interacción farmacéutica
- b. idiosincrasia
- c. tolerancia
- d. antagonismo

Pregunta 18
Sin responder aún
Puntúa como 1,00
Marcar pregunta
Editar pregunta

Señale la respuesta correcta con relación a la absorción rectal:

Seleccione una:
- a. los fármacos son degradados por las enzimas digestivas
- b. en veterinaria se utiliza muchísimo esta vía de administración
- c. Se evita totalmente el efecto de primer paso hepático
- d. la absorcion es irregular

QUESTIONNAIRE I

Veterinary Pharmacology

Área personal ▸ Mis cursos ▸ 0106019 ▸ General ▸ Cuestionario TUBAVET ▸ Vista previa

NAVEGACIÓN POR EL CUESTIONARIO

1 2 3 4 5 6 7 8
9 10 11 12 13 14 15 16
17 18 19 20

Terminar intento...

Tiempo restante 0:27:22

Comenzar una nueva previsualización

NAVEGACIÓN

Área personal
- Inicio del sitio
- Páginas del sitio
- Mi perfil
- Mis cursos
  - 1105225
  - 1105235

Pregunta 19
Sin responder aún
Puntúa como 2,00
Marcar pregunta
Editar pregunta

Entre las reacciones de metabolización en fase I se encuentra la reacción de [▾], mientras que entre las de fase II se incluye la reacción de [▾]

Pregunta 20
Sin responder aún
Puntúa como 1,00
Marcar pregunta
Editar pregunta

En el caso de la administración de fármacos por vía intramuscular:

Seleccione una:
- a. se puede incrementar la absorción aplicando, p.ej., frío
- b. la absorción es mucho más lenta que por vía subcutánea u oral
- c. la administración es muy dolorosa
- d. ninguna es correcta

Siguiente

QUESTIONNAIRE I

Veterinary Pharmacology

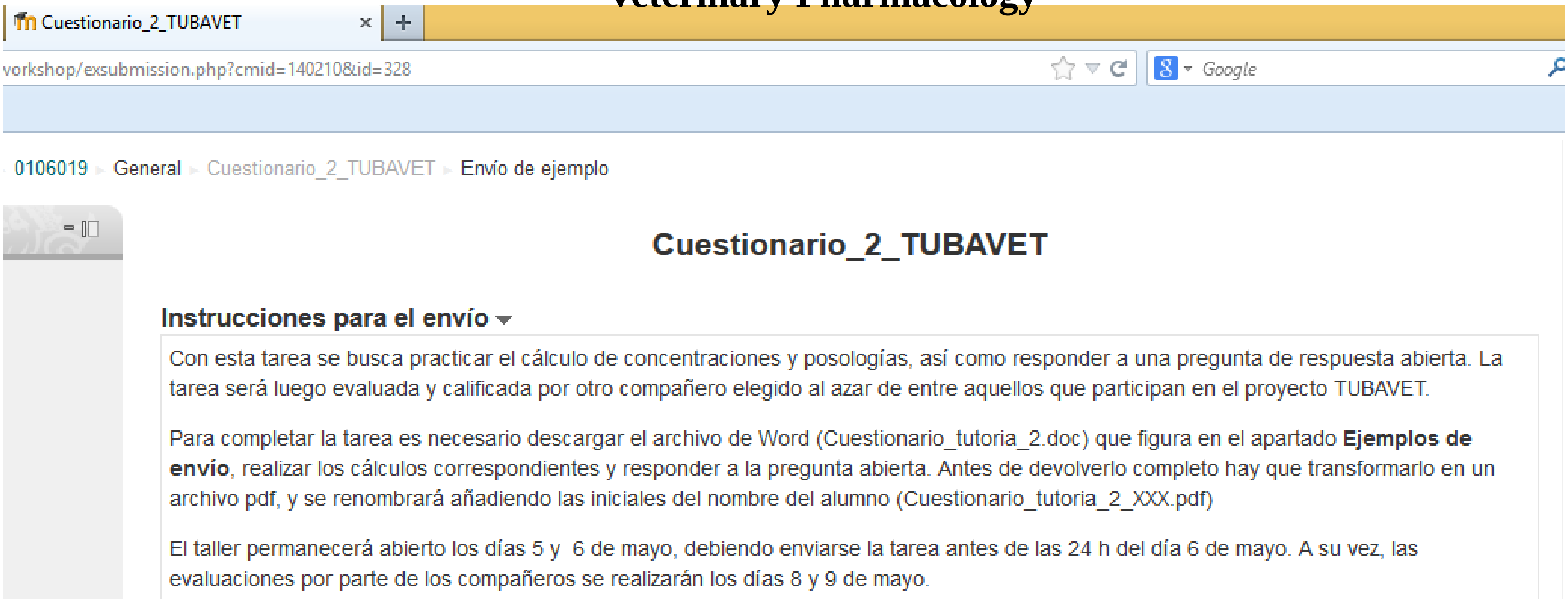

Cuestionario_2_TUBAVET

vorkshop/exsubmission.php?cmid=140210&id=328

0106019 ▸ General ▸ Cuestionario_2_TUBAVET ▸ Envío de ejemplo

Cuestionario_2_TUBAVET

Instrucciones para el envío

Con esta tarea se busca practicar el cálculo de concentraciones y posologías, así como responder a una pregunta de respuesta abierta. La tarea será luego evaluada y calificada por otro compañero elegido al azar de entre aquellos que participan en el proyecto TUBAVET.

Para completar la tarea es necesario descargar el archivo de Word (Cuestionario_tutoria_2.doc) que figura en el apartado **Ejemplos de envío**, realizar los cálculos correspondientes y responder a la pregunta abierta. Antes de devolverlo completo hay que transformarlo en un archivo pdf, y se renombrará añadiendo las iniciales del nombre del alumno (Cuestionario_tutoria_2_XXX.pdf)

El taller permanecerá abierto los días 5 y 6 de mayo, debiendo enviarse la tarea antes de las 24 h del día 6 de mayo. A su vez, las evaluaciones por parte de los compañeros se realizarán los días 8 y 9 de mayo.

QUESTIONNAIRE II

Veterinary Pharmacology

1. El veterinario indica la administración de 2 mL de salicilato de sodio. ¿Cuántos mg se administran si el vial contiene 120 mg/5 cm^3?

2. Una presentación de cefalotina contiene el compuesto a una concentración del 0,45% (m/v). Calcule, en cg, la cantidad de fármaco administrada en: a) 3 mL; b) 1,25 cL.

3. Se ha administrado un compuesto por vía intravenosa a una dosis de 225 mg, distribuyéndose en el organismo siguiendo un modelo monocompartimental. La constante de eliminación del compuesto es 0,15 h^{-1}. Calcule la vida media ($t_{1/2}$) del compuesto en min.

4. Debe tratarse una vaca Frisona de 445 kg con danofloxacino. La concentración de la solución inyectable utilizada es del 2,5% (m/v). La dosis recomendada es 1,25 mg/kg, que se administraría 3 veces a intervalos de 24 h. Calcule, en mg, qué cantidad de principio activo hay que administrar en cada administración y en todo el tratamiento. Describa también cómo realizaría la administración por vía intramuscular, sabiendo que no se pueden administrar más de 20 mL en un mismo punto.

5. Describa el mecanismo de acción de los AINE, así como su acción farmacológica a nivel gastrointestinal y renal.

QUESTIONNAIRE III

Veterinary Pharmacology

0106019 ▸ General ▸ Cuestionario_3_TUBAVET ▸ Información

Cuestionario_3_TUBAVET

A continuación se presenta una batería de 20 preguntas relacionadas con la segunda parte del temario visto en la asignatura de *Farmacología Veterinaria*.

Como en la ocasión anterior, las preguntas son de diversos tipos (opción múltiple con una única respuesta correcta, preguntas de emparejamiento o para rellenar huecos).

Se dispone de 25 minutos para rellenarlo, y cada respuesta incorrecta penaliza en un 20%. No te olvides, por favor, de enviarlo una vez finalizado. Muchas gracias.

Intentos permitidos: 1

Límite de tiempo: 25 minutos

QUESTIONNAIRE III

Veterinary Pharmacology

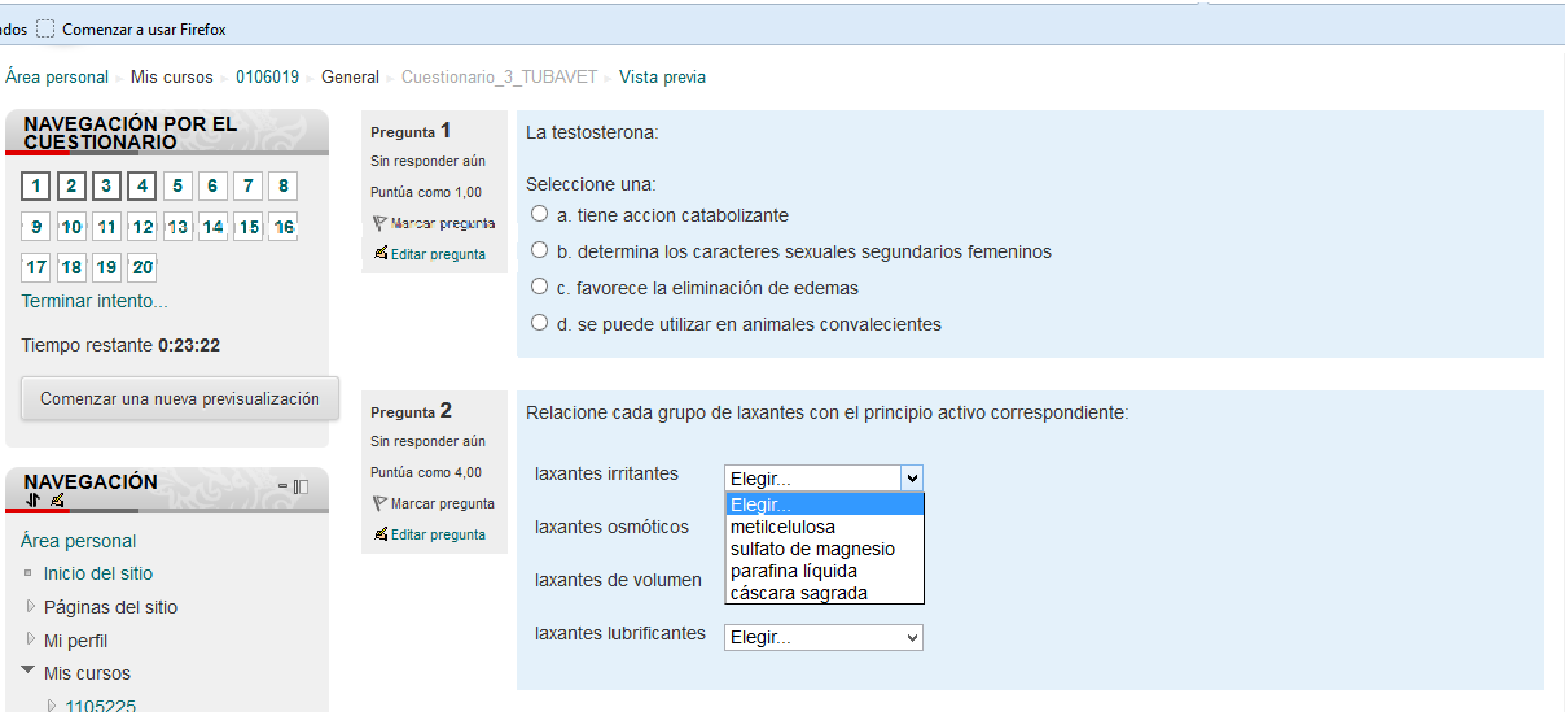

QUESTIONNAIRE III

Veterinary Pharmacology

?attempt=180886

Google

Pregunta 3

Sin responder aún

Puntúa como 1,00

Marcar pregunta

Editar pregunta

La loperamida está indicada en el tratamiento de:

Seleccione una:

- a. estreñimiento
- b. fiebre
- c. tiña
- d. diarreas

Pregunta 4

Sin responder aún

Puntúa como 1,00

Marcar pregunta

Editar pregunta

De los siguientes microorganismos, indique aquellos que sean los más sensibles a la acción de los antisépticos/desinfectantes:

Seleccione una:

- a. bacterias Gram (-)
- b. virus
- c. bacterias Gram (+)
- d. esporas

Siguiente

QUESTIONNAIRE III

Veterinary Pharmacology

Más visitados Comenzar a usar Firefox

NAVEGACIÓN POR EL CUESTIONARIO

1 2 3 4 5 6 7 8
9 10 11 12 13 14 15 16
17 18 19 20

Terminar intento...

Tiempo restante **0:20:59**

Comenzar una nueva previsualización

NAVEGACIÓN

Área personal
- Inicio del sitio
- Páginas del sitio
- Mi perfil
- Mis cursos
  - 1105225
  - 1105235
  - 1719013
  - 1710010
  - 1710011

Pregunta 5
Sin responder aún
Puntúa como 1,00
Marcar pregunta
Editar pregunta

La trimetoprima se asocia con:

Seleccione una:
- a. cefalosporinas
- b. cisplatino
- c. sulfamidas
- d. quinolonas

Pregunta 6
Sin responder aún
Puntúa como 1,00
Marcar pregunta
Editar pregunta

De los siguientes compuestos, indique aquel que actúe inhibiendo la síntesis de la pared celular:

Seleccione una:
- a. cefoperazona
- b. sulfadimidina
- c. tilmicosina
- d. oxitetraciclina

Pregunta 7
Sin responder aún
Puntúa como 1,00
Marcar pregunta
Editar pregunta

El compuesto que modifica las características de la secreción traqueo-bronquial, de forma que la expectoración se vuelve más fluida y cómoda, se denomina

- demulcente
- mucolítico
- fibrinolítico
- antitusivo

QUESTIONNAIRE III

Veterinary Pharmacology

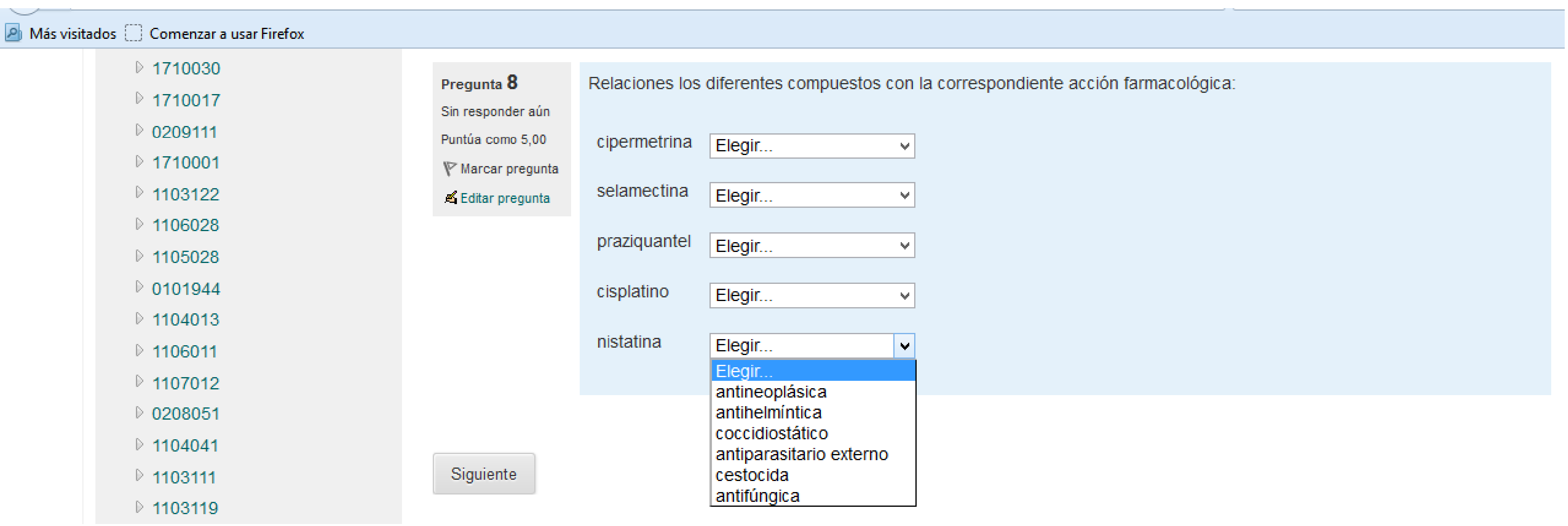

QUESTIONNAIRE III

Veterinary Pharmacology

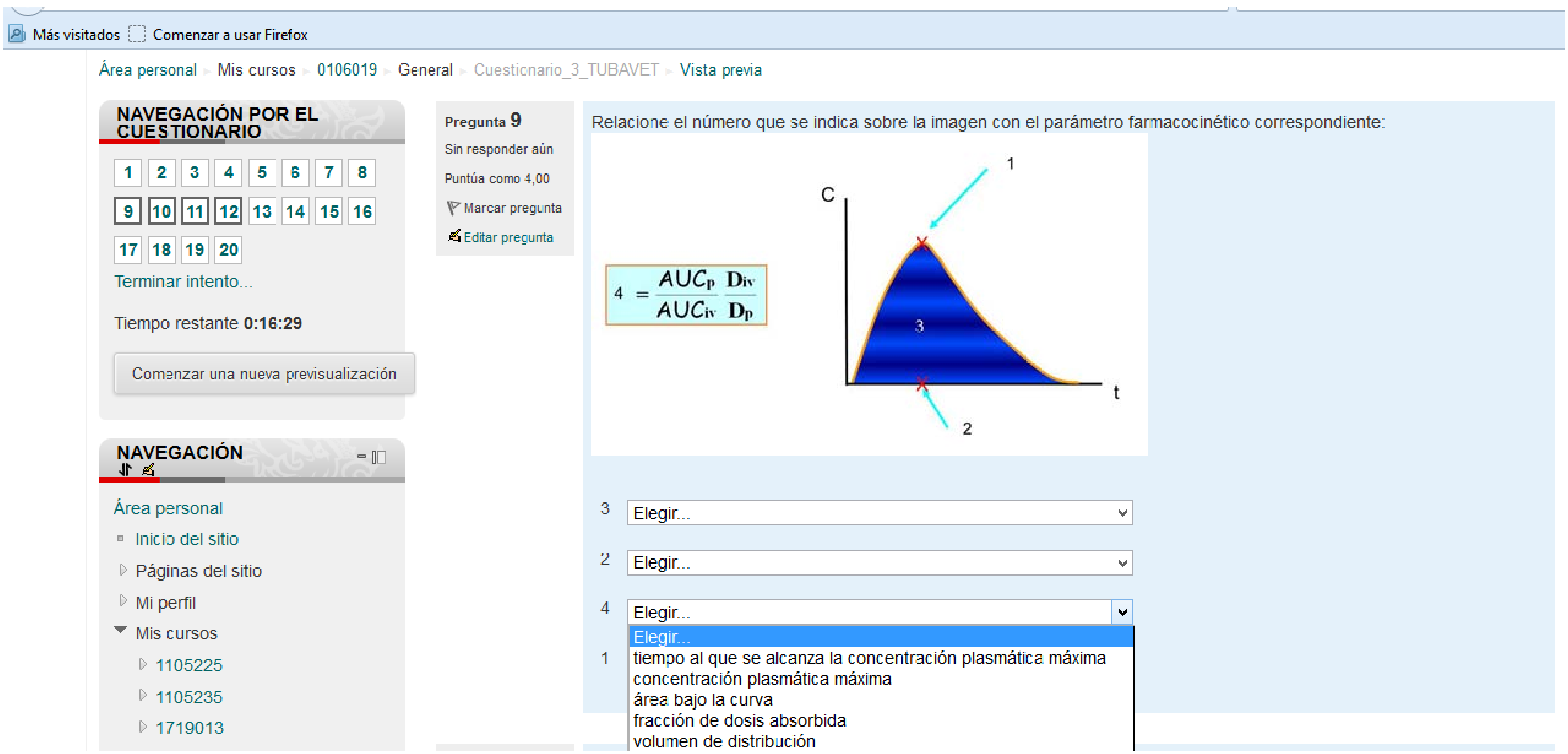

QUESTIONNAIRE III

Veterinary Pharmacology

t.php?attempt=180886&page=2

Google

Pregunta 10

Sin responder aún

Puntúa como 1,00

Marcar pregunta

Editar pregunta

La insulina:

Seleccione una:

- a. promueve la utilización de glucosa por los tejidos
- b. produce lipolisis
- c. produce hiperglucemia
- d. inhibe la secreción gástrica

Pregunta 11

Sin responder aún

Puntúa como 1,00

Marcar pregunta

Editar pregunta

Entre las reacciones adversas de los corticoides se incluyen:

Seleccione una:

- a. curación de las úlceras pépticas
- b. hipercalcificación ósea
- c. deshidratación intensa
- d. retraso en la cicatrización de las heridas

Pregunta 12

Sin responder aún

Puntúa como 1,00

Marcar pregunta

Editar pregunta

Las hormonas tiroideas:

Seleccione una:

- a. reducen el metabolismo basal
- b. reducen la actividad cardíaca
- c. aumentan el consumo de O_2 en los tejidos
- d. inhiben el crecimiento en todos los tejidos del organismo

QUESTIONNAIRE III

Veterinary Pharmacology

NAVEGACIÓN POR EL CUESTIONARIO

1 2 3 4 5 6 7 8
9 10 11 12 13 14 15 16
17 18 19 20

Terminar intento...

Tiempo restante 0:13:19

Comenzar una nueva previsualización

NAVEGACIÓN

Área personal
- Inicio del sitio
- Páginas del sitio
- Mi perfil
- Mis cursos
  - 1105225
  - 1105235
  - 1719013
  - 1710010
  - 1710011
  - 1710030

Pregunta 13
Sin responder aún
Puntúa como 1,00
Marcar pregunta
Editar pregunta

En un timpanismo espumoso estaría indicado el uso de:

Seleccione una:
- a. plantago
- b. no hay posibilidad de tratamiento
- c. ácido cólico
- d. poloxaleno

Pregunta 14
Sin responder aún
Puntúa como 1,00
Marcar pregunta
Editar pregunta

La valnemulina:

Seleccione una:
- a. inhibe la síntesis de ácido fólico en las bacterias
- b. está relacionada con los antibióticos beta-lactámicos
- c. está indicada en el tratamiento y prevención de la disentería porcina
- d. tiene acción bactericida

Pregunta 15
Sin responder aún
Puntúa como 1,00
Marcar pregunta
Editar pregunta

De las siguientes acciones, indique la que corresponda a la prostaglandina F_{2alfa} (PGF_{2alfa}):

Seleccione una:
- a. relajación miometrial
- b. feminización
- c. bajada de la leche
- d. luteolítica

QUESTIONNAIRE III

Veterinary Pharmacology

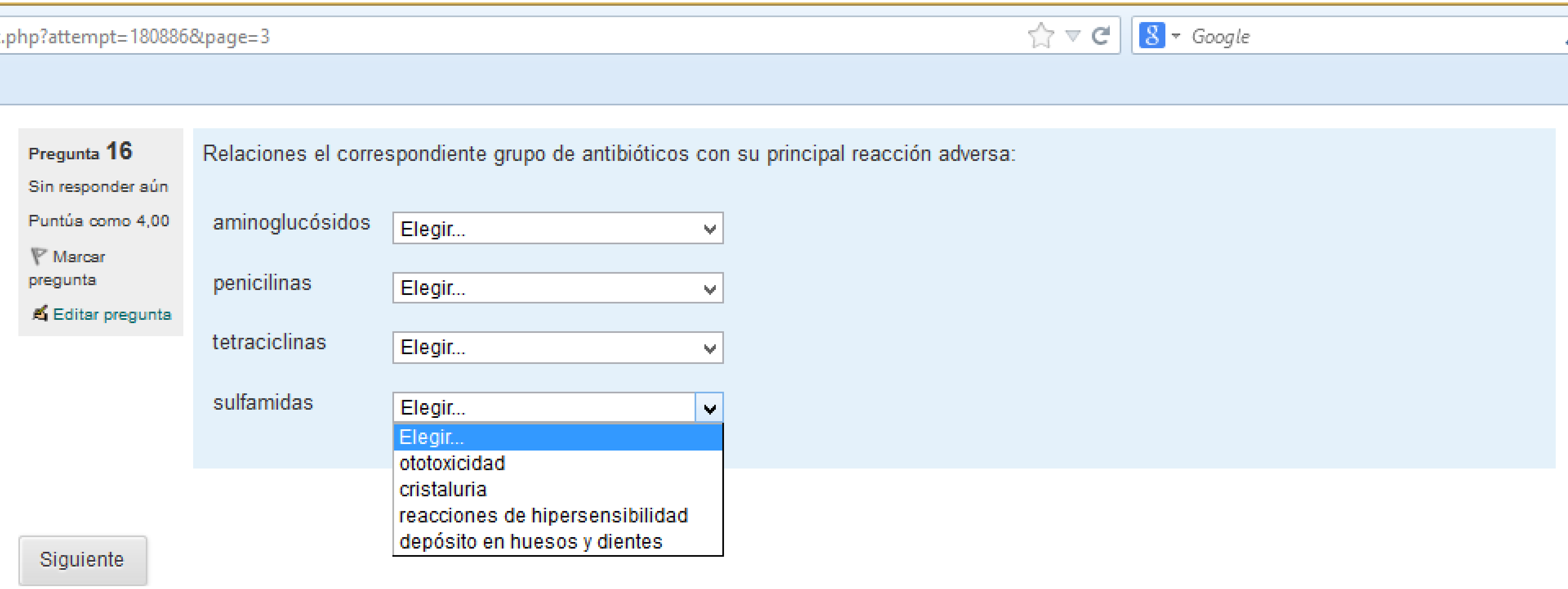

QUESTIONNAIRE III

Veterinary Pharmacology

Más visitados Comenzar a usar Firefox

Área personal ▸ Mis cursos ▸ 0106019 ▸ General ▸ Cuestionario_3_TUBAVET ▸ Vista previa

NAVEGACIÓN POR EL CUESTIONARIO

1 2 3 4 5 6 7 8
9 10 11 12 13 14 15 16
17 18 19 20

Terminar intento...

Tiempo restante **0:10:50**

Comenzar una nueva previsualización

NAVEGACIÓN

Área personal
- Inicio del sitio
- Páginas del sitio
- Mi perfil
- Mis cursos
  - 1105225
  - 1105235
  - 1719013
  - 1710010
  - 1710011
  - 1710030
  - [illegible]

Pregunta **17**
Sin responder aún
Puntúa como 1,00
Marcar pregunta
Editar pregunta

Señale la respuesta correcta con relación a la ivermectina:

Seleccione una:
- a. no necesita fijar tiempo de espera en la leche
- b. actúa estimulando la acción del glutamato en los nematodos
- c. los perros Collie presentan una especial susceptibilidad a este compuesto
- d. se emplea en el tratamiento de cestodosis

Pregunta **18**
Sin responder aún
Puntúa como 1,00
Marcar pregunta
Editar pregunta

Señale la respuesta correcta con relación al cloranfenicol:

Seleccione una:
- a. se utiliza sobre todo en animales destinados al consumo humano
- b. es un compuesto muy seguro, que no produce prácticamente reacciones adversas
- c. actúa a nivel de los ácidos nucleicos de la bacteria
- d. es un antibiótico de amplio espectro

Pregunta **19**
Sin responder aún
Puntúa como 1,00
Marcar pregunta
Editar pregunta

La heparina:

Seleccione una:
- a. estimula la síntesis de diversos factores de coagulación
- b. tiene acción agregante
- c. atraviesa la barrera fetoplacentaria
- d. se puede utilizar tanto *in vivo* como *in vitro*

CUESTIONARIO III
Farmacología Veterinaria

Pregunta 20
Sin responder aún
Puntúa como 1,00
Marcar pregunta
Editar pregunta

Aquel agente que inhibe el crecimiento o destruye microorganismos sobre tejido inanimado, se denomina

- desinfectante
- antiséptico
- esterilizante

Siguiente
